# Interventions to improve social circumstances of people with mental health conditions: a rapid evidence synthesis

**DOI:** 10.1186/s12888-022-03864-9

**Published:** 2022-04-28

**Authors:** Phoebe Barnett, Thomas Steare, Zainab Dedat, Stephen Pilling, Paul McCrone, Martin Knapp, Eleanor Cooke, Daphne Lamirel, Sarah Dawson, Peter Goldblatt, Stephani Hatch, Claire Henderson, Rachel Jenkins, T K, Karen Machin, Alan Simpson, Prisha Shah, Martin Stevens, Martin Webber, Sonia Johnson, Brynmor Lloyd-Evans

**Affiliations:** 1 Centre for Outcomes Research and Effectiveness, Research Department of Clinical, Educational and Health Psychology, University College London, 1-19 Torrington Place, London, WC1E 7HB UK; 2 Department of Psychiatry, Mental Health Policy Research Unit, University College London, London, UK; 3 National Collaborating Centre for Mental Health, Royal College of Psychiatrists, London, UK; 4 Camden and Islington NHS Foundation Trust, London, UK; 5 Institute of Lifecourse Development, University of Greenwich, London, UK; 6 Care Policy and Evaluation Centre, Department of Health Policy, London School of Economics and Political Science, London, UK; 7 Camden and Islington NHS Foundation Trust and MH Policy Research Unit, London, UK; 8 Department of Population Health Sciences, Bristol Medical School, University of Bristol, Bristol, England; 9 Department of Epidemiology & Public Health, Institute of Health Equity, University College London, London, UK; 10 Institute of Psychiatry, Psychology and Neuroscience, Department of Psychological Medicine, Kings College London, London, UK; 11 ESRC Centre for Society and Mental Health, Kings College London, London, UK; 12 Institute of Psychiatry, Psychology and Neuroscience, Health Services and Population Research Department, Kings College London, London, UK; 13 South London and Maudsley NHS Foundation Trust, London, UK; 14 Institute of Psychiatry, Psychology and Neurology, Kings College London, London, UK; 15 Mental Health Policy Research Unit Lived Experience Working Group, Department of Psychiatry, University College London, London, UK; 16 Florence Nightingale Faculty of Nursing, Kings College London, Midwifery & Palliative care, London, UK; 17 NIHR Policy Research Unit On Health and Social Care Workforce Research Unit, King’s College London, London, UK; 18 International Centre for Mental Health Social Research, Department of Social Policy and Social Work, University of York, York, England

**Keywords:** Review, Social circumstances, Mental health conditions, Intervention

## Abstract

**Background:**

Poor social circumstances can induce, exacerbate and prolong symptoms of mental health conditions, while having a mental health condition can also lead to worse social outcomes. Many people with mental health conditions prioritise improvement in social and functional outcomes over reduction in clinical symptoms. Interventions that improve social circumstances in this population should thus be considered a priority for research and policy.

**Methods:**

This rapid evidence synthesis reports on randomised controlled trials of interventions to improve social circumstances across eight social domains (Housing and homelessness; money and basic needs; work and education; social isolation and connectedness; family, intimate and caring relationships; victimisation and exploitation; offending; and rights, inclusion and citizenship) in people with mental health conditions. Economic evaluations were also identified. A comprehensive, stepped search approach of the Cochrane library, MEDLINE, Embase, PsycINFO, Web of Science and Scopus was conducted.

**Results:**

One systematic review and 102 randomised controlled trials were included. We did not find RCT evidence for interventions to improve family, intimate and caring relationships and only one or two trials for each of improving money and basic needs, victimisation and exploitation, and rights, inclusion and citizenship. Evidence from successful interventions in improving homelessness (Housing First) and employment (Individual Placement and Support) suggests that high-intensity interventions which focus on the desired social outcome and provide comprehensive multidisciplinary support could influence positive change in social circumstances of people with mental health conditions. Objective social isolation could be improved using a range of approaches such as supported socialisation and social skills training but interventions to reduce offending showed few benefits. Studies with cost and cost-effectiveness components were generally supportive of interventions to improve housing and vocational outcomes. More research is needed to ensure that social circumstances accompanied by high risks of further exacerbation of mental health conditions are adequately addressed.

**Conclusions:**

Although there is a large body of literature examining how to support some aspects of life for people with mental health conditions, more high-quality evidence is required in other social domains. Integration into mental health services of interventions targeting social circumstances could significantly improve a number of social outcomes.

**Supplementary Information:**

The online version contains supplementary material available at 10.1186/s12888-022-03864-9.

## Introduction

Social circumstances, including lack of or difficulties with social relationships, social adversity, and socio-economic factors, have a bi-directional association with mental health [1], being both influential determinants and consequences of mental health problems. Identifying effective interventions that improve the social circumstances of people with mental health conditions (disorders which persistently affect emotion, thinking and behaviour [2]) is therefore a priority for several reasons. First, many mental health service users prioritise social and functional outcomes over clinical outcomes [3], and there are calls for mental health services to increase their emphasis on social issues including social inclusion, rights and community participation [4, 5], and for professionals to orient their practice towards recovery, focusing on the goals that matter to service users, which are often social [6]. People with mental health conditions, especially those whose difficulties are relatively severe and long-term, have specific and additional needs compared to the general population. They may find generally available support accessible and helpful in many areas of life, but in some social domains, such as employment, tailored approaches may achieve better outcomes [7].

Second, the prevalence of adverse social circumstances is high in people with mental health conditions, at high personal and societal cost [1]. People with mental health conditions are more likely to be unemployed despite most service users wishing to work [8–10], and thus miss out on associated opportunities for financial security, personal development, social contact and status within society [11]. Having poor mental health also places people at increased risk of crime or violence [12–14], difficulties with family roles such as parenting [15], loneliness, and discrimination [16–18]. People’s needs for support, and therefore the burden on families of people with mental health conditions is also extremely high as a result of the adverse social circumstances they face [19]. There is a clear case for increased action to reduce the social adversity that compounds difficulties accompanying mental illness for many.

Third, the bi-directional association between social circumstances and mental health signifies that the alleviation of social adversity could also have benefits on clinical outcomes. Mental health appears to follow a socio-economic gradient, such that the risk of poor mental health increases in line with greater social adversity [20, 21]. Although the relationship is complex, social circumstances can have a role in both the onset and the continuation of mental disorders [22] and can also be a significant barrier to accessing effective treatment [23]. It has been argued that despite some advances in mental health treatments, there is little evidence that this has led to major improvements in prognosis or quality of life for people with longer-term mental health conditions [1], suggesting the need for additional treatment targets and support aimed at alleviating social adversity.

Fourth, economic and social adversities resulting from the coronavirus pandemic are likely to have disproportionate impacts on people with pre-existing mental health conditions [24], especially those who belong to Black, Asian and Minority Ethnic groups affected especially severely by the COVID-19 pandemic [25–27] or who have been confined in poor quality homes, lack social support or live with others with whom they have problematic relationships. National policy initiatives will be a major driver of economic recovery and population mental health, including for people with mental health conditions, but individual level social interventions may be important in alleviating the additional burdens experienced by people with severe mental health conditions.

It is therefore important to collate available evidence about interventions aimed at improving the social circumstances of people with mental health conditions, to identify effective ways of supporting this group that warrant further investigation and/or wider implementation, and to identify evidence gaps and priorities for further research. We conducted a rapid evidence synthesis [28] of systematic reviews and randomised controlled trials regarding the effectiveness of socially-focused interventions for individuals with mental health conditions. To the best of our knowledge this is the first evidence synthesis to collate the available evidence within a single review about interventions across a broad range of areas of people’s lives: We focus on the following eight social domains: housing and homelessness; money and basic needs; work and education; social isolation and connectedness; family, intimate and caring relationships; victimisation and exploitation; offending; and rights, inclusion and citizenship.

## Methods

This review was conducted by the National Institute of Health Research (NIHR) Mental Health Policy Research Unit, intended to inform national and international service planning and policy making. An initial decision was made to focus on eight life-domains which relate to people’s social circumstances, were considered relevant to quality of life and health outcomes, and were identified as priorities by policy makers from the Department of Health and Social Care (DHSC) and Public Health England (PHE). Research questions and definitions for each domain were refined through consultation in a stakeholder working group including people with relevant expertise such as mental health lived experience, health and social care practitioners, national policy makers, and academics. The following review questions were formulated through this consultation process:

- **Housing and homelessness:** What are the effects of interventions for people with mental health conditions aimed at improving housing and reducing homelessness on: a) achieving/sustaining independent living? b) quality/acceptability of housing?
- **Money and basic needs:** What are the effects of interventions for people with mental health conditions aimed at alleviating poverty and debt on a) reducing poverty/increasing income? b) reducing financial barriers to meeting basic needs (e.g. food, fuel and transport)? c) reducing or managing debt?
- **Work and education:** What are the effects of interventions for people with mental health conditions aimed at improving work and education on a) **finding** paid employment? b) retention of paid employment? c) length of sickness absence for mental health conditions from paid employment? d) access to/completion of educational courses or qualifications? e) engagement in meaningful activity (apart from paid work)?
- **Social isolation and connectedness: w**hat are the effects of interventions for people with mental health conditions aimed at preventing or reducing social isolation and improving connectedness on a) **subjective** social isolation (loneliness and perceived lack of social support)? b) objective social isolation (number of social contacts)? c) social capital (access to social resources within a social network)?
- **Family, intimate and caring relationships:** What are the effects of interventions for people with mental health conditions aimed at improving **family** and caring relationships on: a) achieving and sustaining roles in inter-personal relationships (including as an intimate partner, a parent or family member)? b) maintenance of informal caring roles (e.g., caring for an unwell or infirm relative)?
- **Victimisation and exploitation:** What are the effects of interventions for people with mental health conditions aimed at reducing victimisation and exploitation on a) prevention of victimisation or repeat victimisation as a result of crime (in general, and specifically sexual assault, domestic violence or coercive control)? b) reduction and prevention of exploitation or harassment?
- **Offending:** What are the effects of interventions for people with mental health conditions who are also offenders aimed at reducing offending on a) offending and reoffending? b) successful community living following criminal conviction or time in prison?
- **Rights, inclusion and citizenship:** What are the effects of interventions for people with mental health conditions aimed at improving rights, **inclusion** and citizenship on a) increasing social inclusion or participation? b) improving access to rights and public services? c) addressing lack of privacy or dignity resulting from social circumstances?

The protocol was prospectively registered on PROSPERO (CRD42020191780) and we adhered to PRISMA guidelines [29].

### Search strategy

A stepped, iterative approach was taken to searching, first for systematic reviews then for randomised controlled trials (RCTs), in order to efficiently capture the extensive range of relevant literature across all domains of social circumstances included in this review while limiting duplication of work. A combination of keyword and subject heading searches were used. In addition, experts in the fields of social domains were contacted and asked to recommend relevant systematic review literature. Searching was conducted in six electronic databases by an experienced information scientist (SD) with expertise in mental health literature: the Cochrane Library (Cochrane Database of Systematic Reviews (CDSR) (inception-February 2020) an Cochrane Central Register of Controlled Trials (2000-August 2020); Ovid MEDLINE (inception-February 2020 Systematic reviews (SR) only); Ovid Embase (inception February 2020 SR only); Ovid PsycINFO (inception-February 2020 SR and 2000-August 2020 RCT); Web of Science Social Citations Index (SSCI) and Science Citation Index (SCI) (inception-February 2020 SR only); SCOPUS (inception-February 2020 SR only). An additional search during the systematic review phase was conducted on the Ovid platform, to correct a spelling mistake and to incorporate additional terms for loneliness/social isolation. A Pragmatic decision was taken not to search MEDLINE or Embase for RCTs as we considered Cochrane’s centralised search process to adequately capture RCT records from these databases [30] for this rapid evidence synthesis.

RCT searches were conducted with a date limit of 2000-August 2020 except where an identified high-quality systematic review (searches in at least three databases, quality appraisal conducted, inclusion of RCTs) already covered part of this period, which applied for the following domains:

- Employment: Searches were conducted separately for severe mental illness (SMI; Psychosis, schizophrenia, bipolar disorder or similar) and common mental disorder (CMD; depression, anxiety, post-traumatic stress disorder or similar) populations, with SMI trials limited to studies published after 2017, to supplement the high quality Cochrane review of interventions to improve employment in people with SMI found at the systematic review level [7]. Trials of interventions aimed at patients with CMDs were retrieved from 2000, as for other searches.
- Social isolation and loneliness: Terms for social isolation and loneliness were searched from 2017 onwards, due to finding a review [31] with a search conducted in 2017 which encompassed (and extended) our inclusion criteria for interventions to reduce loneliness in people with mental health conditions. Interventions meeting our inclusion criteria in this review (K = 9) were extracted and summarised along with additional RCTs found, as the review included an additional 21 studies which did not meet our inclusion criteria, and therefore conclusions from the review were not considered entirely relevant to our research questions. Trials of interventions to improve social participation and social capital were retrieved from 2000, as for other searches.

These searches used refined search terms based on included papers from the systematic review stage. A full overview of the search strategy is available in Additional File 1.

### Selection criteria

We included studies (at both the systematic review and RCT stage) from high-income countries (defined as the 38 countries within the Organisation for Economic Cooperation and Development (OECD); [32]). Systematic reviews published at any time were included. Although RCT searches were limited to the year 2000 onwards, there was no date restriction for included RCTs found through systematic reviews. Studies additionally had to meet the following criteria:

#### Participants

Adults aged 18 + with any mental health condition or a diagnosis of personality disorder (other than specified excluded conditions), established through clinical diagnosis, meeting threshold criteria on an established diagnostic screening tool or symptom severity measure or users of specialist mental health services (minimum 80% of sample).

##### Exclusions

Intellectual/learning disability, dementia or other organic mental disorder, neurodevelopmental disorder or acquired cognitive impairment, anti-social personality disorder, adjustment disorder, substance use disorder (in the absence of any mental illness or personality disorder).

#### Interventions

Non-pharmacological interventions designed to improve social circumstances in any of the included life domains (Table 1) where this was the primary outcome or otherwise described in the paper as an explicit, direct focus of the intervention.

**Table 1:** Life domains included in the review

Life domain	Relevant social circumstances
Housing and homelessness	Homelessness Housing instability (achieving and sustaining tenancies) Housing quality (individual housing and neighbourhoods)
Money and basic needs	Poverty/income Financial barriers to essential resources (including food and fuel poverty and availability, access to transport) Debt Money management
Work and education	Unemployment (achieving and sustaining paid employment – including. open market and sheltered work) Precarious work Lack of access to or completion of educational goals Lack of meaningful activity (including voluntary work) Length of illness absence/time to return to work from sick leave due to mental health conditions
Social isolation and connectedness	Subjective social isolation/loneliness Objective social isolation/social network Social capital
Family, intimate and caring relationships	Difficulties with: Partner/sexual relationships (achieving or sustaining a relationship) Maintaining parenting roles or contact with children Maintaining contact or cohabitation with family members Caring responsibilities (maintaining caring role)
Victimisation and exploitation	Victim of crime (general) Sexual or physical assault Domestic violence and coercive control Exploitation, harassment and safeguarding concerns
Offending	Risk of offending (prevention/diversion from offending) Transition from prison to community Reoffending
Rights, inclusion and citizenship	Social exclusion/difficulties with social participation (including digital exclusion) Difficulties with access to public services Immigration status (resolution of status, access to support) Lack of privacy or dignity resulting from social circumstances

Interventions designed to improve more than one life domain, e.g. through helping people access available services, groups or community resources were also included where improving overall social circumstances was the primary aim of the programme.

#### Exclusions

In social isolation and family, intimate and caring relationships domains, we limited included outcomes for individual relationships to the maintenance or gain of social roles (e.g. retention of partner relationship, parental contact, carer role) and so excluded subjective outcomes relating to the perceived quality of individual social relationships. Following discussion with the stakeholder working group, this was operationalised as excluding outcomes relating to: i) individual perceived relationship quality including parent–child attachment and partner relationship or parenting quality, ii) family relationship quality including expressed emotion, and iii) experienced or self-stigma.

#### Comparator

Comparators of routine care, no support or an active intervention were all included.

#### Outcomes

Included studies needed to report at least one outcome specifically relating to the social circumstances listed in Table 1.

#### Study design

English-language Systematic reviews and RCTs, for each stage of searching, were included. Feasibility and pilot trials were also included; however, it is acknowledged that non-significance in these trials does not necessarily imply that the intervention was ineffective, and we considered this in our synthesis.

### Study selection

During the first stage of the search, all titles and abstracts of systematic reviews were screened by one of five reviewers (PB, TS, DL, EC, ZD) using the Rayyan application [33]. Systematic reviews not meeting inclusion criteria were excluded. Full texts of reviews were examined for relevance to our research questions: We considered ‘fully relevant’ reviews those in which at least 80% of included studies would also be included in the current review, and which searched at least three databases. Partially relevant reviews included some RCTs which would meet inclusion in the current review but had additional inclusion criteria meaning that conclusions drawn may not be directly relevant to our research questions. Those considered fully relevant, and of sufficient quality were included in the review and those considered partially relevant were retained and included studies within them were screened.

**Fig. 1:**
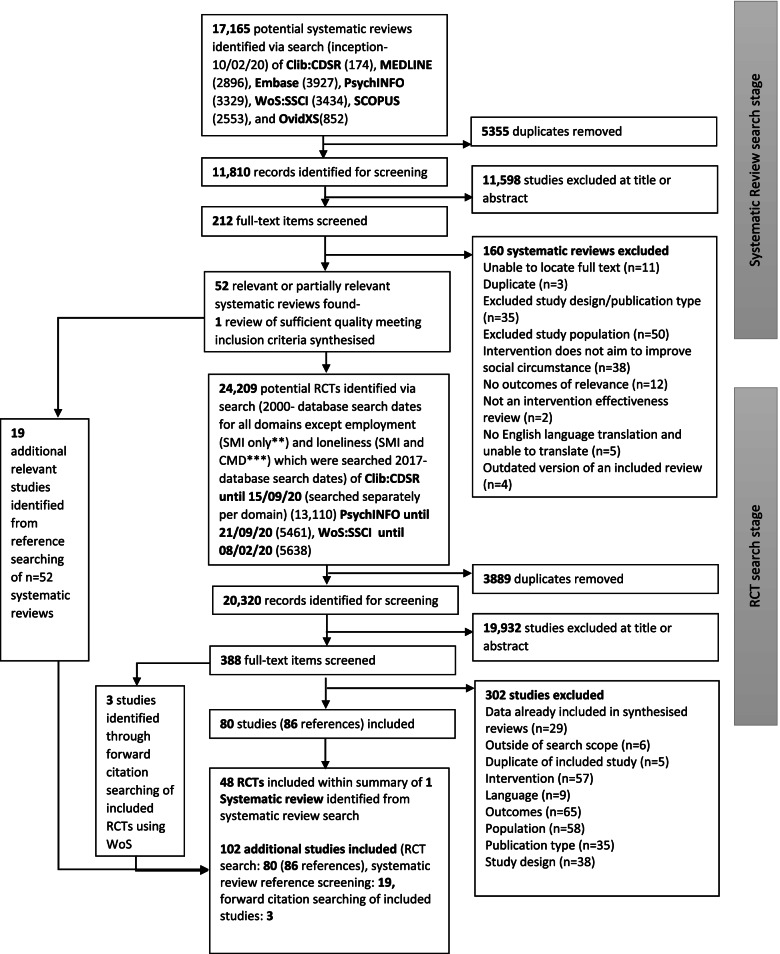
PRISMA diagram

During the second stage of the search, all titles and abstracts of RCTs were also independently screened by one of five reviewers (PB, TS, DL, EC, ZD). Studies not meeting inclusion criteria were excluded. Full-text articles were subsequently reviewed by one of two reviewers (TS, PB). A third senior reviewer (BLE) resolved all unclear cases through discussion with PB and TS. The full search and screening process is depicted in Fig. 1.

### Data extraction

Six reviewers (PB, TS, DL, EC, NL, ZD) extracted the data from RCTs using an excel form. Data extracted included: Demographic and clinical sample characteristics, intervention detail, following the TIDIER [34] checklist and methodological characteristics of the study, including study variables such as setting and sample size, to inform quality assessment. For our primary outcomes (measures of social need) we extracted one outcome per paper to answer each research question/study objective stated within the protocol. We use the study’s primary outcome where relevant and stated; otherwise we followed a hierarchy of preference for the most relevant outcomes, which we developed with authors with expertise in the area (SJ and SP) (see Additional File 2). Secondary outcomes extracted included: Mental health symptoms, quality of life, and costs. For each secondary outcome, a similar hierarchy of preference was followed, resulting in one measure per outcome being extracted (see Additional File 2). Outcome timepoints were measured from baseline due to the large proportion of studies reporting interventions without a specific end-point, and were classed as short term (< 6 months), medium-term (6–12 months), and long term (12 + months). Where multiple intervention arms were reported, we extracted all interventions which were sufficiently distinct. In studies reporting additional comparison interventions or less intensive, non-distinct variations of an intervention these were not extracted. Systematic reviews deemed of sufficient relevance and quality (*N* = 1) to include were narratively summarised. Ten percent of all extraction conducted by each reviewer was double checked by a second reviewer. All disagreement was resolved through discussion with a senior reviewer (BLE).

### Quality assessment

Six reviewers (PB, TS, DL, EC, NL, ZD) assessed the methodological quality of included studies. The Cochrane Risk of Bias tool [35] was used to assess the quality of RCTs. Selection, performance, detection, attrition, and reporting bias were classified as unclear, low or high risk for each study. The AMSTAR tool [36] was used to assess the quality of the included systematic review.

### Data analysis

We synthesised the results using narrative synthesis [37]. We organised studies around their targeted social domain, target population (severe mental illness (SMI), common mental disorder (CMD) or mixed or unspecified mental health conditions), and treatment type. To achieve a feasible means of categorising study populations, we considered SMI as bipolar disorder schizophrenia and other psychotic disorders and CMD as depression of all severities, anxiety disorders, and post-traumatic stress disorder.. We produced summary tables for each social outcome, and secondary outcome. We did not carry out any meta-analysis due to the heterogeneity of populations, interventions, and intervention intensities, however, where possible we converted reported statistics for each study into standardised mean difference (SMD, continuous outcomes) and odds ratios (OR, dichotomous events data) to ease interpretation. For social isolation outcomes, we classed interventions according to the typology proposed by Mann and colleagues [38] and used in a recent systematic review [31] (See Table 2). For employment gain and retention, we classed interventions according to the typology outlined by Suijkerbuijk et al. [7]. For remaining social domains, we classed each intervention as containing (or not containing) different types of care identified as important by the review team and our stakeholder working group. As interventions were complex, they often contained multiple components. The results reported from trials of specific interventions are reported in detail in Tables 2, 3, 4, 5, 6, 7.

**Table 2:** Social isolation and connectedness outcomes

Mental health diagnoses	Intervention type	Author	Intervention vs Control	Outcomes
*Subjective social isolation*				
CMD	Changing cognitions	Conoley 1985 (USA, *N* = 38) [39]	Reframing vs Waitlist control	Measures of loneliness did not differ between groups at 1 month (Hedges g = 0.11, 95% CI: -0.53, 0.75),
	Psychoeducation	Haslam 2019 (Australia, *N* = 120) [40]	Groups 4 Health social identity intervention vs TAU	The intervention group reported significantly reduced loneliness compared to the control group 2 months after baseline (Odds of reduced loneliness = 3.84, 95% CI: 1.50, 9.81)
	Supported socialisation	Lloyd-Evans 2020 (UK, *N* = 40) [41]	Community navigator programme + routine care vs Routine care	[Feasibility trial] Loneliness in the intervention group fell from a median De Jong Gierveld Scale score of 11 at baseline to 9 at follow up, and from 10.5 to 10 for the control group participants. This change was not significant although numbers were small
SMI	Changing cognitions	Hasson-Ohayon 2014 (Israel, *N* = 55) [42]	Social cognition and interaction training (SCIT) + social mentoring vs social mentoring only	Participants who completed SCIT showed significant improvement between baseline and post assessment in mean scores for social engagement compared with participants in the control group, whose scores decreased. Hedges g at end of treatment (6 months): 1.44, 95% CI: 0.84, 2.05)
	Psychoeducation	Silverman 2014 (USA, *N* = 45) [43]	Live music therapy and Education vs Education only	Perceived social support did not differ significantly between the live music therapy and education and the education only groups after the session (Hedges g = 0.47, 95% CI: -0.13, 1.07)
	Supported socialisation	Gelkopf 1994 (Israel, *N* = 34) [44]	Watching comedy films with others vs Watching a variety of film genres	There was no significant difference in satisfaction with social support at 4-months follow-up (control 10.66 vs comedy 15.19, *F* = 1.90, not significant)
		Terzian 2013 (Italy, *N* = 357) [45]	social network intervention + TAU vs TAU only	A higher overall social network improvement—including an improvement in intimate or working relationships—was reported at year 1 for the experimental treatment patients (OR = 1.8, 95% CI 1.12, 2.80). The results were still statistically significant at year 2 ( OR = 1.84, 95% CI 1.18, 2.90)
		Davidson 2004 (USA, *N* = 260) [46]	Matched with a volunteer partner who had a personal history of psychiatric disability vs Matched with a volunteer partner who had no history of psychiatric disabilities vs Not matched with a volunteer partner	When considering all participants, there were no significant improvements in self-reported socialisation scores between groups at end of treatment (volunteer partner with no history of psychiatric problems vs control at end of treatment (9 months): Hedges g = 0.05, 95% CI: -0.34, 0.43, volunteer partner with a history of psychiatric problems vs control at end of treatment (9 months): Hedges g: -0.11, 95% CI: -0.49, 0.28)
		Sheridan 2015 (Ireland, *N* = 107) [47]	Supported socialisation vs monetary support	There was no group (*F* = 0·78, *p* = 0.38), or group x time effect (*F* = 1.33, *p* = 0.36) for social loneliness (Social and Emotional Loneliness Scale for Adults)
	Mixed approach- supported socialisation and psychoeducation	Boevink 2016 (Netherlands, *N* = 163) [48]	Recovery and self-help training course “TREE Recovery programme” + TAU vs TAU	After 1 year, the patients in the TREE recovery programme did not have significantly lower loneliness scores compared to treatment as usual. (Hedges g = -0.11,95% CI: -0.44, 0.23)
		Castelein 2008 (Netherlands, *N* = 106) [49]	Guided peer support VS TAU	There was no significant difference between groups in the extent of discrepancies between desired and received in social support at 8 months (Hedges g adjusted for baseline values: = -0.09, 95% CI: -0.29, 0.47)
Mixed mental health conditions	Supported socialisation	Rivera 2007 (USA, *N* = 203) [50]	Peer-assisted case management vs Standard case management	There was no significant difference between peer assisted case management and standard case management in the subjective quality of social relations at either 6 months post baseline (Hedges g = -0.08, 95% CI: -0.43, 0.26) or 12 months post baseline (Hedges g = -0.09, 95% CI: -0.43, 0.25)
*Social capital*				
CMD	Supported socialisation	Lloyd-Evans 2020 (UK, *N* = 40) [41]	Community navigator programme + routine care vs Routine care	[Feasibility trial] Median perceived social capital (social network resourcefulness) changed from 7.0 to 7.5 in the intervention group, and 11.5 to 11.0 in the control group
*Objective social isolation*				
SMI	Changing cognitions	Hasson-Ohayon 2014 (Isreal, *N* = 55) [42]	Social cognition and interaction training + social mentoring vs social mentoring only	There was no significant difference between the two groups on interpersonal communication at end of treatment (6 months). Hedges g = -0.06, 95% CI: -0.60, 0.48
		Pos 2019 (Netherlands, *N* = 99) [51]	CBT for social activation vs TAU	There were no significant between group differences in social withdrawal at either 3 months post baseline (Hedges g = -0.04, 95% CI: -0.43, 0.36) or 9 months post baseline (Hedges g = 0.03, 95% CI: -0.36, 0.43)
		Pot-Kolder 2018 (Netherlands, *N* = 116) [52]	Virtual reality CBT vs TAU	Differences between groups in the amount of time spent with others at end of treatment or 6 months post baseline were not significant Hedges g at end of treatment (3 months) = 0.31, 95% CI: -0.06, 0.67, Hedges g at follow up (6 months) = 0.29, 95% CI: -0.08, 0.65
		Roberts 2014 (USA, *N* = 66) [53]	Social cognition and interaction training vs TAU	The Global social functioning scale did not exhibit a Treatment group x Time interaction but did show a statistically significant main effect for treatment group, *F*(1, 56) = 5.65, *P* < .05. Follow-up analyses revealed that SCIT participants received higher global functioning ratings than TAU participants when controlling for baseline scores at 6 months (*P* < .05). Accordingly, the SCIT group showed a small to medium effect size advantage over TAU at follow-up (d = .43)
	Social skills training	Glynn 2004 (USA, *N* = 63) [54]	Skills training + generalization vs skills training only	Participation in clinic-based plus in vivo amplified skills training was associated with significantly greater improvements compared to clinic based only in overall adjustment (condition-by-time interaction) (*F* = 4.88 (1, 40), *P* = .04) as assessed with the Social Adjustment Scale-II at 12 months post baseline
		Marder 1996 (USA, *N* = 80) [55]	Social skills training vs supportive group therapy	There were significant effects favouring social skills training over supportive group therapy on total social functioning at 24 months (*F* = 6.05, df = 1, 94, *P* = 0.02) when considering all patients
	Supported socialisation	Gelkopf 1994 (Isreal, *N* = 34) [44]	Comedy films vs variety of film genres	The comedy Intervention group had significantly more distinct network members than the control group at 4 months follow-up (control 2.87 vs comedy 5.22, *F* = 4.87, *P* < 0.05)
		Priebe 2020 (UK, *N* = 124) [56]	Matched with a volunteer partner who had no history of psychiatric disabilities vs not matched with a volunteer partner	Patients in the intervention group had significantly more social contacts after treatment, when controlling for baseline scores (adjusted difference = 0.52, 95% CI: 0.04, 0.99, *P* = 0.03) and the analyses comparing the groups at the 6-month follow-up showed that patients in the intervention group still had significantly more social contacts (baseline-adjusted difference = 0.73, 95% CI: 0.05, 1.40, *P* = 0.04)
	Mixed approach- supported socialisation and psychoeducation	Castelein 2008 (Netherlands, *N* = 106) [49]	Guided peer support vs waitlist	A higher proportion of participants in the intervention group had a significant increase in contact with peers outside of the sessions at end of treatment (8 months) in comparison with the waitlist control condition (OR = 2.83, 95% CI: 1.59, 5.06)
	Mixed approach-changing cognitions and social skills training	Granholm 2005 (USA, *N* = 76) [57]	Cognitive behavioural social skills training vs TAU	The treatment group reported significantly more mean social activities on the social adjustment scale compared to treatment as usual at 6 months (Hedges g = 0.60, 95% CI: 0.14, 1.06)
Mixed mental health conditions	Supported socialisation	Rivera 2007 (USA, *N* = 203) [50]	Peer-assisted case management vs Standard case management	Only clients receiving peer-assisted care showed a significant increase in the number of contacts from baseline to 12 months (simple effect: *F* = 7.25, df = 2 and 118, *P* < .01, η2 = .11). However, follow-up analyses revealed that this effect was due to increased contact with peer assistants and professional staff, not with family and outside friends When considering total network size without these staff, there was no significant difference in social network size between the two groups at either end of treatment (6 months): Hedges g = 0.187, 95% CI: -0.17, 0.54 or 12 months post baseline (Hedges g = 0.22, 95% CI: -0.14, 0.58)

*N *Number of participants*, SMI *Severe mental illness*, CMD *Common mental disorder*, TAU *Treatment as usual*, CBT *Cognitive behavioural training*,* *OR* Odds ratio*, CI *Confidence interval

**Table 3:** Housing and homelessness outcomes

Mental health diagnoses	Author	Intervention vs Control	Badged as 'Housing First'	Independent tenancy	Staff on site/supported housing	multi-disciplinary team mental health support (e.g. ACT, ICM)	Housing support worker practical support (no multi-disciplinary team support)	Specified psychological therapy offered	Outcomes
	*Achieving/Sustaining Housing*								
SMI	Aubry 2016 (Canada, *N* = 950) [58]	Housing First + ACT vs TAU	Y	Y	N	Y	N	N	Housing First participants spent a significantly higher percentage of time in stable housing compare to the control group at 6 months (Hedges g = 1.41, 95% CI: 1.26, 1.55), 12 months (Hedges g = 1.14, 95% CI: 1.00, 1.27) and 24 months (Hedges g = 0.59, 95% CI: 0.46, 0.72) post baseline
	Tinland 2020 (France, *N* = 703) [59]	Housing First + ACT vs treatment as usual	Y	Y	N	Y	N	N	The Housing First + ACT group had significantly more mean days stably housed at 6 months from baseline (Hedges g = 2.22, 95% CI: 2.04, 2.41), 12 months from baseline (Hedges g = 1.81, 95% CI: 1.64, 1.99) and 24 months from baseline (Hedges g = 1.38, 95% CI: 1.21, 1.54)
	Stergiopoulos 2015 (Canada, *N* = 1198) [60]	Housing First plus integrated case management vs treatment as usual	Y	Y	N	Y	N	N	The Housing First plus integrated case management group spent a significantly higher mean number of days stably housed than the treatment as usual group at 24 months (Hedges g = 0.85, 95% CI: 0.73, 0.97)
	Tsemberis 2004 (USA, *N* = 206) [61]	Pathways Housing First vs continuum of care	Y	Y	N	Y	N	N	a repeated measures ANOVA showed a time x group status effect such that participants in the experimental condition had significantly faster decreases in homelessness (*F*(4, 137) = 10.1, *P* < .001) and increases in stable housing (*F*(4, 137) = 27.7, *P* < .001) relative to control participants
	Burnam 1996 (USA, *N* = 276) [62]	a) Social model residential treatment programme b) Social model non-residential treatment programme vs no intervention	a) N b) N	a) N b) N	a) Y b) N	a) Y b) Y	a) N b) N	a) N b) N	There were no significant differences between residential and non-residential housing interventions, except that non-residential housing participants were more likely to have increased the amount of time they spent in independent housing at 3 months following baseline. this is expected because residential housing participants were by definition not stably housed
	Ellison 2020 (USA, *N* = 166) [63]	Manualized treatment model for co-occurring mental illness and substance use disorders (MISSION-Vet) vs TAU	N	N	N	N	N	Y	Treated veterans did not spend more days in housing compared with control veterans during any part of the study at 95% level of confidence
	Fletcher 2008 (USA, *N* = 161) [64]	Integrated ACT vs standard care	N	N	N	Y	N	Y	There was no significant difference between the integrated Assertive Community Treatment and the control group in the mean number of days stably housed (15 months: Hedges g = 0.25, 95% CI: -0.10, 0.61, 30 months: Hedges g = 0.31, 95% CI: -0.04, 0.66) However, the following variables mediated the IACT vs. SC effect on stable housing: programme contacts, substance abuse contacts, help with activities of daily living, transportation assistance, and, help with medication
	Goldfinger 1999 (USA, *N* = 118) [65]	Group housing vs independent housing	N	N	Y	Y	N	N	The intervention group were not significantly more likely to be in stable housing than independent housing control group at the end of 18-month follow-up period (OR = 1.09, 95% CI: 0.45, 2.63)
	Herman 2011 (USA, *N* = 150) [66]	Critical time intervention + usual care vs usual care	N	N	N	Y	N	N	At 18 months, the OR of experiencing homelessness in the intervention group compared to the control group during the final three follow up intervals was 0.22 (95% CI:0.06, 0.88) when controlling for baseline homelessness, indicating that the intervention group were less likely to experience homelessness
	Hurlburt 1996 (USA, *N* = 361) [67]	a) Sect. 8 rent subsidy certificate vs no Sect. 8 certificate b) Comprehensive housing services vs traditional housing services	a) N b) N	a) N b) N	a) N b) N	a) N b) Y	a) Y b) N	a) N b) N	a) a very strong relationship appeared between Sect. 8 housing condition and housing outcomes. Most of this effect was on type of community housing obtained- of those who achieved stable housing, those on Sect. 8 were 4.87 times likely to achieve stability in independent housing than those in the non-Sect. 8 condition. However, when categories of independent housing and other consistent housing were combined, Sect. 8 clients were only 1.21 times more likely to achieve some type of consistent housing than non-Sect. 8 clients Section 8 certificates were strongly associated with obtaining independent housing, regardless of substance abuse diagnosis b) There was no difference in housing stability when traditional services were compared to comprehensive services
	Korr & Joseph 1995 (USA, *N* = 95) [68]	Case management vs routine care	N	N	Y	N	Y	N	At 6 months post baseline, over twice as many experimental clients as control clients were housed (OR = 6.4, 95% CI: 2.61, 15.68)
	Lehman 1997 (USA, *N* = 152) [69]	ACT vs usual community services	N	N	N	Y	N	N	The ACT group spent significantly more days in the 12-month follow-up housed (210.2 vs 160.1, Hedges g = 0.46, 95% CI: 0.14, 0.78)
	Lipton 1988 (USA, *N* = 49) [70]	Residential treatment vs standard care	N	N	N	Y	N	N	More participants in the intervention group were in permanent housing at 12 months than the control group (OR = 5.19, 95% CI: 2.84, 9.48)
	McHugo 2004 (USA, *N* = 121) [71]	Integrated housing vs Parallel housing	N	N	Y	Y	N	N	The integrated housing condition did not spend a significantly higher mean number of days in stable housing at 6 months (Hedges g = 0.20, 95% CI: -0.16, 0.55, *P* = 0.287), however, they spent significant more days in stable housing at both 12 months (Hedges g = 0.50, 95% CI: 0.14, 0.86) and 18 months (Hedges g = 0.50, 95% CI: 0.134, 0.86)
	Morse 1992 (USA, *N* = 116) [72]	Continuous treatment team vs outpatient mental health services	N	N	N	Y	N	N	A significant treatment-by-time interaction was found for days homeless (*F* = 4.23, df = 2,97, *P* = .017). Post hoc analyses indicated that the clients in the continuous treatment team programme were less likely to be homeless at 12 months than those in the outpatient programme, however, endpoint analyses at 12 months did not show a significant group effect (Hedges g = 0.29, 95% CI: -0.17, 0.75)
	Morse 2006 (USA, *N* = NR) [73]	Integrated ACT vs standard care	N	N	N	Y	N	Y	The main effect of treatment on stable housing was statistically significant at 24 months follow up, *F*(2, 145) = 3.76, *p* = .03. Post-hoc analyses indicated that clients in IACT condition had significantly more days in stable housing than control clients. There was no significant treatment by time interaction, *F*(6, 440) = 1.93, *p* = .07
	Shern 2000 (USA, *N* = 168) [74]	Community outreach (Choices) vs Treatment as usual	N	N	Y	N	Y	N	Participants in the community outreach programme reported a 23% increase in the proportion of time spent in shelters (t = -5.73, *P* < .001) compared to the control group at 24 months
	Susser 1997 (USA, *N* = 96) [75]	Critical time intervention vs usual services	N	N	N	N	Y	N	During the last month of the 18- month follow-up, 4 (8%) of the men in the CTI group and 11 (23%) of the men in the USO group were homeless, a significant difference (OR of not being homeless = 3.46, 95% CI: 1.01, 11.80, *P* = 0.047)
Mixed mental health conditions	Morse 1997 (USA, 165) [76]	a) Broker case management vs ACT b) ACT with community workers vs ACT	a) N b) N	a) N b) N	a) N b) N	a) Y b) Y	a) N b) N	a) N b) N	A significant treatment group effect was found on days in stable housing (*F* = 3.54, df = 2,129, *P* < 0.032), such that Assertive Community Treatment only participants averaged more days in stable housing at 18 months than clients in both broker case management and Assertive Community Treatment with community workers
	*Housing Quality*								
SMI	Aubry 2016 (Canada, *N* = 950) [58]	Housing First + ACT vs TAU	Y	Y	N	Y	N	N	Compared with treatment-as-usual participants, Housing First participants rated their housing as being of significantly better quality at 6 months (Hedges g = 0.60, 95% CI: 0.47, 0.73), 12 months (Hedges g = 0.57, 95% CI: 0.44, 0.70) and 24 months from baseline (Hedges g = 0.22, 95% CI: 0.09, 0.34)
	Lehman 1997 (USA, *N* = 152) [69]	ACT vs usual community services	N	N	N	Y	N	N	The ACT programme subjects were significantly more satisfied with their housing at the 6-month follow-up (Hedges g = 0.37, 95% CI: 0.05, 0.68), but not at 12-month follow-up (Hedges g = 0.09, 95% CI: -0.26, 0.44)
	Lipton 1988 (USA, *N* = 49) [70]	Residential treatment vs standard care	N	N	N	Y	N	N	The experimental group, with a mean score of 1.63 at 12 months, indicated that on average they were satisfied with and committed to their housing arrangements. At 12 months the controls’ mean rating of their living arrangements was 2.87, indicating that on average they perceived inadequacies and desired an alternative (Hedges g = 1.25, 95% CI: 0.50, 2.00)

*N* Number of participants*, SMI* Severe mental illness*, TAU* Treatment as usual*, CBT* Cognitive behavioural training*, ACT* Assertive Community Treatment*, OR* Odds ratio*, CI* Confidence interval*, Y* Yes*, N* No

**Table 4:** Work and education outcomes for gaining and retaining paid employment, and education enrolment

Mental health diagnoses	Category	Author	Intervention vs Control	Outcomes
*Finding paid employment*				
CMD	Transitional employment + psychiatric care	Beutel 2005 (Sweden, *N* = 63) [77]	Occupational training integrated into psychodynamic treatment vs TAU	At 12 months post baseline, there was no significant difference between the intervention and control groups in the likelihood of being employed (OR = 1.11, 95% CI: 0.63, 1.96), however, at 24 months, the occupational training and psychodynamic treatment intervention group were significantly more likely to be unemployed than the treatment as usual group (OR = 2.71, 95% CI: 1.51, 4.84)
	Prevocational training (Job related skills training)	Schene 2007 (Netherlands, *N* = 62) [78]	Adjuvant occupational therapy + TAU vs TAU	While after 6 months, there was no difference between the intervention and control group in terms of the proportion of participants engaged in part time work (16 + hours), OR of part time work = 0.80, 95% CI: 0.32, 2.02, the adjuvant occupational therapy intervention group resulted in significantly more participants being employed part time at both 12 months (OR = 3.62, 95% CI: 1.83, 7.15) and 24 months post baseline (OR = 1.84, 95% CI: 1.05, 3.24)
	Supported employment (Low fidelity/not IPS)	Hellstrom 2017 (Denmark, *N* = 326) [79]	IPS modified for people with mood and anxiety disorders vs TAU	There was no significant difference between the IPS and TAU groups in the number of participants returning to competitive work at either 12 months (OR = 1.18, 95% CI: 0.73, 1.90) or 24 months (OR = 1.32, 95% CI: 0.85, 2.05)
	Supported employment (High fidelity IPS)	Davis 2012 (USA, *N* = 85) [80]	IPS vs TAU	IPS participants were significantly more likely to gain competitive employment by 12 months than the TAU control group (OR = 8.27, 95% CI: 3.12, 21.89)
		Davis 2018 (USA, *N* = 541) [81]	IPS vs transitional work	IPS participants were significantly more likely to be competitively employed at 6 months (OR = 2.4, 95% CI: 1.74, 3.49), 12 months (OR = 2.19, 95% CI: 1.55, 3.10) and 18 months (OR = 1.65, 95% CI: 1.16, 2.34)
SMI	Transitional employment	Bell 1993 (USA, *N* = 100) [82]	Paid work vs unpaid work	Significantly more participants in the pay condition accepted work (35% vs 5%, OR of accepting work = 10.23, 95% CI: 3.81, 27.50)
	Transitional employment + Cognitive skills training	Bell 2005 (USA, *N* = 145) [83]	Work therapy + neurocognitive enhancement vs work therapy	The NET + WT condition had a higher percentage of patients having competitive-wage employment at 12 months post baseline, however, this difference was not significant (OR of competitive employment = 1.51, 95% CI: 0.73, 3.14, 0.275)
		Bell 2018 (USA, *N* = 77) [84]	Vocational rehabilitation + cognition remediation vs vocational rehabilitation + cognitive games	Rate of competitive employment at 12 months did not differ between the cognitive remediation and cognitive games control group (OR = 1.11, 95% CI: 0.38, 3.21)
		McGurk 2016 (USA, *N* = 54) [85]	Enhanced vocational services + cognitive remediation (thinking skills for work) vs enhanced vocational services only	Although more participants in the thinking skills for work intervention obtained competitive work at 36 month follow up (57% vs. 48%), these differences were not statistically significant (OR = 1,56, 95% CI: 0.53, 4.56)
		Mervis 2017 (USA, *N* = 64) [86]	Indianapolis vocational rehabilitation programme vs supportive therapy	Nearly half of the intervention group went on to secure supported employment by the 12-month follow-up (14 out of 29; 48%), compared to 17% of those in the SG condition (6 out of 35, 17%) (OR of supported employment = 4.51, 95% CI: 1.44, 14.13)
		Vauth 2005 (Germany, *N* = 93) [87]	Computer assisted cognitive strategy training + Vocational rehabilitation vs vocational rehabilitation	The rate of successful job placement (more than 3 months of half- or full-time employment, or at least sheltered workshop jobs) was higher in the computer assisted cognitive strategy training intervention group than the vocational rehabilitation only control at 12 months (OR of successful job placement = 2.96, 95% CI: 1.24, 7.07)
	Prevocational training with cognitive therapy	Fowler 2019 (UK, *N* = 77) [88]	Social recovery CBT + TAU vs TAU	In the combined sample of individuals with affective and non-affective psychosis, more individuals in the social recovery + TAU group had engaged in paid work over the 15 months since the end of the intervention period compared to the TAU alone group (31.0% vs. 16%), however this difference was not significant (OR of paid work = 2.33, 95% CI: 0.72, 7.54)
	Prevocational training (cognitive skills training)	Lindenmayer 2008 (USA, *N* = 85) [89]	Cognitive remediation vs computerized control	Among the 37 unemployed patients at baseline in the cognitive remediation group, 51% obtained a job during the 12-month follow-up, compared with 35% of 31 initially unemployed patients in the control group, which was not a statistically significant difference (OR = 1,92, 95% CI: 0.72, 5.10)
		Russinova 2018 (USA, *N* = 51) [90]	Vocational empowerment photovoice vs wait-list	The VEP intervention group did not have significantly different rates of engagement in employment services at the post 10-week core VEP curriculum assessment (OR of engagement in employment services = 2.8, 95% CI: 0.71, 11.03), the posttreatment assessment following the last booster session (OR = 2.4, 95% CI: 0.65, 8.86) or the 3-month follow-up assessment point (OR = 0.59, 95% CI: 0.18, 1.96)
	Prevocational training (job related skills training)	Gutman 2009 [education focused] (USA, *N* = 38) [91]	BRDGE supported education programme vs TAU	Only 1 intervention group participant obtained paid employment, while no control group participants obtained paid employment (non-significant difference)
		Rogers 2006 (USA, *N* = 135) [92]	Psychiatric vocational rehabilitation vs enhanced state vocational rehabilitation	There was no significant difference between the intervention and control group at either 9 months (OR = 2.42, 95% CI: 0.40, 2.60), or 24 months (OR = 1.03, 95% CI: 0.40, 2.60) in the number of participants with competitive work
	Supported employment (Low fidelity/not IPS)	Cook 2008 (USA, *N* = 1273) [93]	Supported employment vs TAU	Participants in the supported employment group had significantly higher rates of competitive employment than the control group at 24 months (OR = 3.79, b = 1.33 (SE: 0.12), *P* < 0.001), controlling for gender, race, education, drug/alcohol use, intellectual disability, disability beneficiary status, prior work history, age, months worked in prior 5 years, physical health, work motivation, age at first hospitalization, lifetime months hospitalised, psychotic symptoms, study site and attrition
	Supported employment (High fidelity IPS)	Erickson 2020 (Canada, *N* = 109) [94]	IPS + TAU vs TAU	There was no significant difference in the proportion of participants employed in the past 6 months at either 6 months (OR = 0.88, 95% CI:0.42, 1.88) or 12 months (OR = 1.73, 95% CI: 0.81, 3.70)
		Killackey 2019 (Australia, *N* = 106) [95]	IPS vs TAU	At the end of the intervention (6 months), the IPS group had a significantly higher rate of having been employed (71.2%, 47/66) than the TAU group (48%, 29/60), OR = 3.40 (95% CI 1.17, 9.91, *P* = 0.025, controlling for employment at baseline).; however, no significant between-group differences in odds of employment were seen at 6–12 and 12–18 months (*P* = 0.288 and *P* = 0.594, respectively)
	Augmented supported employment (SE + cognitive skills training)	Bell 2008 (USA, *N* = 77) [96]	Neurocognitive enhancement + vocational rehabilitation vs vocational rehabilitation only	no significant difference in odds of being employed at 12 months (end of intervention) or 24 months post baseline (12 months OR = 1.08, 95% CI:0.60, 1.95, 24 months OR = 1.57, 95% CI: 0.82, 3.00)
		McGurk 2007 (USA, *N* = 44) [97]	Supported employment + cognitive training vs supported employment	Over the first year, significantly more clients in supported employment with cognitive training worked (69.6%) than those in the supported employment only programme (4.8%). Similarly, at 24 months, significantly more patients worked in the supported employment with cognitive training programme than the supported employment alone programme (OR = 13.71, 95% CI: 3.03, 62.14)
		McGurk 2015 (USA, *N* = 107) [98]	Enhanced supported employment + cognitive remediation (thinking skills for work) vs enhanced supported employment only	Significantly more participants in Thinking skills for work group obtained competitive employment at 24 months (60% vs 36%, OR = 2.63, 95% CI: 1.20, 5.75)
		Rodriguez Pulido 2019 (Spain, *N* = 57) [99]	IPS plus cognitive remediation vs IPS	The IPS plus cognitive remediation group had higher odds of being employed in the past 6 months at both 12 months (OR = 2.65, 95% CI: 1.48, 4.74) and 16 months from baseline (OR = 2.60, 95% CI: 1.47, 4.59)
		Twamley 2019 (USA, *N* = 153) [100]	Compensatory cognitive training vs enhanced supported employment	Compensatory cognitive training did not result in significantly more participants attaining competitive employment compared to supported employment at 24 months (OR = 0.58, 95% CI: 0.33, 1.01)
	Augmented supported employment (SE + cognitive therapy)	Lecompte 2019 (Canada, *N* = 164)	CBT for supported employment vs supported employment	Those who received CBT-SE were significantly more likely to obtain at least one job (OR = 3.36, 95% CI: 1.75, 6.46) after 1 year than the supported employment only control group
	Augmented supported employment (SE + job related skills training)	Glynn 2017 (USA, *N* = 67) [101]	IPS plus work skills training vs IPS only	Over 24 month follow up, there was no difference between the IPS plus work skills training group and the IPS only group in the time to first job (Hedges g = 0.22, 95% CI: -0.27, 0.70)
		Kern 2018 (USA, *N* = 58) [102]	IPS + errorless learning vs IPS	at 12 months, 40.7% of the errorless learning plus supported employment group (11/27) were still continuously working compared to 13.8% of the supported employment alone group (4/29) which was statistically significant (OR = 4.29, 95% CI: 2.14, 8.58)
		Nuechterlein 2019 (USA, *N* = 69) [103]	IPS + Workplace fundamentals module vs brokered vocational rehabilitation + social skills training	The IPS + workplace fundamentals intervention group did not show significant differences in the number in competitive employment at 6 months (OR = 0.87, 95% CI: 0.48, 1.59), however, over the following 12 months, this group were significantly more likely to competitively employed than the comparison group (OR = 4.52, 95% CI: 2.49, 8.19)
Mixed mental health conditions	Prevocational training (Job related skills training)	Henderson 2013 (UK, *N* = 80) [104]	Use of a decision aid + TAU vs TAU	[Feasibility trial] More of the intervention group than controls were in full-time employment at follow-up (*P* = 0.03)
	Supported employment (Low fidelity/not IPS)	Okpaku 1997 (USA, *N* = 152) [105]	Employment-orientated case management vs TAU	37 of the 73 participants (51%) in the intervention group got a job of any type by the end of the study (between 3 and 18 months from baseline), while 28 of the 79 control group participants (35%) got a job. This difference was not significant (OR = 1.87, 95% CI = 0.98, 3.59)
	Supported employment (High fidelity IPS)	Reme 2019 (Norway, *N* = 410) [106]	IPS vs TAU	Significantly more IPS participants were employed at 12 months compared to the control group (OR = 1.55, 95% CI: 1.02, 2.37), as well as at 18 months (OR = 1.61, 95% CI: 1.06, 2.46)
		Rossler 2020 (Switzerland, *N* = 78) [107]	IPS with 55 h placement budget vs IPS with 25 h placement budget	According to the cox regression analysis, participants in the 25 h group (control) were more successful at getting their first employment relative to the 55 h group (Hazard ratio = 1.75, 95% CI: 0.86, 3.49). The cumulative proportion of participants who obtained a competitive employment was 33.3% in the intervention (55 h) group and 51.3% in the control (25 h) group
	Augmented supported employment (SE + cognitive skills training)	Bejerholm 2017 (Sweden, *N* = 63) [108]	Individual enabling and support vs TAU	At 6-month follow-up, 12.1% of participants in the individual enabling and support condition were competitively employed, while 14.8% of control participants reached their working goal, a non-significant difference (3% difference in favour of control, OR of employment = 0.79, 95% CI: 0.18, 3.52) At 12-month follow-up, 42.4% of participants in the individual enabling and support condition were competitively employed, while 4% of control participants reached their working goal (38% difference in favour of the intervention group; OR of employment = 17.68, 95% CI: 2.13, 146.77)
		Christensen 2019 (Denmark, *N* = 477) [109]	IPS with cognitive training vs TAU	At 18 months, the IPS intervention group was significantly more likely to be employed or enrolled in education compared to the TAU group (OR = 1.49, 95% CI: 1.03, 2.17)
		Yamaguchi 2017 (Japan, *N* = 92) [110]	Cognitive remediation + supported employment vs usual employment services	The employment rate during the 12 month follow up [number of people working (n) = 28, 62.2%] was significantly higher in the CR + SE group than in the TVS group (*n* = 9, 19.1%, OR = 11.06, 95% CI = 3.53, 34.62, *P* < 0.001 after controlling for site and baseline GAF score)
*Retaining paid employment*				
CMD	Prevocational training (Job related skills training)	Schene 2007 (Netherlands, *N* = 62) [78]	Adjuvant occupational therapy + TAU vs TAU	Patients in the TAU + occupational therapy group worked significantly more median hours in the last 6 months at 6 months (Hedges g = 0.60, 95% CI: 0.08, 1.12, *P* = 0.024)) and 12 months (Hedges g = 0.53, 95% CI: 0.01, 1.04, *P* = 0.044). However, the groups did not differ in the last 6 months at 24 months (Hedges g = 0.30, 95% CI: -0.20, 0.81)
	Supported employment (Low fidelity/not IPS)	Hellstrom 2017 (Denmark, *N* = 326) [79]	IPS modified for people with mood and anxiety disorders vs TAU	There was no significant difference between groups in the mean number of weeks worked at both 12 months post baseline (Hedges g = 0.10, 95% CI: -0.12, 0.32) and 24 months (Hedges g = 0.16, 95% CI:-0.10, 0.38)
	Supported employment (High fidelity IPS)	Davis 2012 (USA, *N* = 85) [80]	IPS vs TAU	IPS participants worked significantly more weeks in competitive employment 21.6 vs 6.8 during the 12 months from baseline (Hedges g = -093, 95% CI: 0.48, 1.37)
		Davis 2018 (USA, *N* = 541) [81]	IPS vs transitional work	IPS participants spent significantly longer employed in a competitive job, on average, than the transitional work control group (Hedges g = 0.32, 95% CI: 0.15, 0.49) at 18 months follow up
	Augmented supported employment (SE + cognitive therapy)	Overland 2018 (Norway, *N* = 1193) [111]	Work directed CBT and job support programme (At work and Coping) vs TAU	In the full sample, the average (median) number of months with work (and receiving no benefits) were 18.5 (15) for the control group and 20.3 (21) for the intervention group. For the subgroup on long-term benefits, the corresponding numbers were 6.0 (0) and 8.8 (0), respectively
SMI	Transitional employment	Bell 1993 (USA, *N* *=* 100) [82]	Paid work vs unpaid work	The paid work group were significantly more likely to still be in employment at the 6 month follow up (OR = 14.81, 95% CI: 4.11, 53.46)
	Transitional employment + Cognitive skills training	Bell 2018 (USA, *N* = 77) [84]	Vocational rehabilitation + cognition remediation vs vocational rehabilitation + cognitive games	There was no significant difference between the cognitive remediation intervention group and the control group in the number of hours of competitive work at 12 months from baseline (Hedges g = -0.24, 95% CI: -0.73, 0.24)
		McGurk 2016 (USA, *N* = 54) [85]	Enhanced vocational services cognitive remediation (thinking skills for work) vs enhanced vocational services only	There was no significant difference between groups in the mean number of weeks worked after 3 years (Hedges g = -0.04, 95% CI: -0.57, 0.50)
	Transitional employment + cognitive therapy	Kukla 2018 (USA, *N* = 50) [112]	CBT + cognitive remediation vs vocational support	At 6 months post baseline, the CBT + cognitive remediation group did not work significantly more hours across the 6-month intervention phase than the vocational support control (Hedges g = 0.18, 95% CI: -0.38, 0.73)
		Lysaker 2005 (USA, *N* = 50) [113]	Vocational CBT programme vs TAU	Participants in the vocational CBT group worked significantly more weeks than those in the standard support group after 12 months. Hedges g = 0.70, 95% CI: 0.11, 1.29)
	Transitional employment + Social Skills training	Sanches 2020 (Netherlands, *N* = 188) [114]	Boston University approach to psychiatric rehabilitation vs active control condition	During the study period, total hours of participation in employment increased significantly (t-ratio = 2.84, df = 241, *p* = 0.005), with no difference between the conditions (t-ratio = 0.649, df = 97, *P* = 0.518). There were significant effects for fewer baseline psychiatric symptoms (t ratio = − 3.55, df = 97, *P* < 0.001); previous paid employment (t-ratio = 3.54, df = 97, *P* < 0.001); and having received additional support (t-ratio = 2.77, df = 97, *P* = 0.007)
	Prevocational training (Job related skills training)	Bell 2003 (USA, *N* = 63) [115]	Paid work plus behavioural intervention vs paid work only	Participants in the behavioural intervention condition worked significantly more weeks at 6 months from baseline than participants who received usual services (Hedges g = 0.53, 95% CI: 0.03, 1.03)
	Prevocational training (Symptom related, Mindfulness)	Davis 2015 (USA, *N* = 34) [116]	Mindfulness based stress reduction (Mirrors) vs Intensive support control	[feasibility trial] The number of weeks worked at the 24 months (end of intervention) were similar in the mindfulness based stress reduction and intensive support control
	Prevocational training (cognitive skills training)	Lindenmayer 2008 (USA, *N* = 85) [89]	Cognitive remediation vs computerized control	Patients who received cognitive remediation worked significantly more weeks after 12 months than patients in the control group (mean 9.2 vs 3.7, Hedges g = 0.49, 95% CI: 0.00, 0.97)
	Supported employment (High fidelity IPS)	Erickson 2020 (Canada, *N* = 109) [94]	IPS + TAU vs TAU	There were no significant differences between groups at either 6 months (Hedges g = 0.02, 95% CI: -0.36, 0.39) or 12 months (Hedges g = 0.21, 95% CI: -0.16, 0.59) post baseline in the number of days worked
		Killackey 2019 (Australia, *N* = 146) [95]	IPS vs TAU	At 18 months post baseline, the interaction between treatment group and time was not significant, *F*(2, 148.4) = 0.95, *P* = 0.390. Furthermore, the main effects for time, *F*(2, 148.4) = 0.50, *P* = 0.608 and for group, *F*(1,112.9) = 0.20, *P* = 0.652, were not significant, suggesting that IPS did not improve the number of hours worked
	Augmented supported employment (SE + job related skills training)	Glynn 2017 (USA, *N* = 67) [101]	IPS plus work skills training vs IPS only	Over the 24 months of follow up, the IPS + work skills training group did not work significantly more weeks than the IPS only group (Hedges g = -0.30, 95% CI: -0.78, 0.19)
		Kern 2018 (USA, *N* = 58) [102]	IPS + errorless learning vs IPS	Though the IPS + errorless learning group worked more weeks on average, the difference was not significant at 12 months (Hedges g = 0.37, 95% CI: -0.16, 0.89)
		Mueser 2005 (USA, *N* = 35) [117]	Supported employment skills training programme vs TAU	There was no significant difference in the number of days worked in the first job obtained by the participants in the intervention vs the control group (Hedges g = 0.15, 95% CI: -0.51, 0.82)
		Nuechterlein 2019 (USA, *N* = 69) [103]	IPS + Workplace fundamentals module vs brokered vocational rehabilitation + social skills training	At 18 months post baseline, the mean number of weeks in competitive employment did not differ significantly between the two groups (Hedges g = 0.30, 95% CI: -0.22, 0.82)
	Augmented supported employment (SE + symptom related skills training)	Harris 2017 (Australia, *N* = 86) [118]	Cognitive remediation and supported employment vs internet information	At 6-months, those participants randomized to CogRem had worked significantly more hours (*P* = .01) than those participants randomized to the WebInfo control condition. (no additional data)
		McGurk 2007 (USA, *N* = 44) [97]	Supported employment + cognitive training vs supported employment	Significantly more hours over the past 12 months were worked by the cognitive training group compared to the supported employment only group at both 12 months (Hedges g = 1.08, 95% CI: 0.44, 1.71) and 24 months (Hedges g = 0.72, 95% CI: 0.11, 1.33)
		McGurk 2015 (USA, *N* = 107) [98]	Enhanced supported employment + cognitive remediation (thinking skills for work) vs enhanced supported employment only	Participants in the thinking skills for work programme worked significantly more hours over the past 12 months after 24 months follow up (Hedges g = 0.55, 95% CI: 0.16, 0.94)
		Rodriguez Pulido 2019 (Spain, *N* = 57) [99]	IPS plus cognitive remediation vs IPS	There was no significant difference in the average number of weekly hours worked at 12 months post baseline (Hedges g = 0.10, 95% CI: -0.48, 0.67) however at 16 months post baseline, the cognitive remediation group worked significantly more weekly hours (Hedges g = 1.26, 95% CI: 0.63, 1.89)
		Twamley 2019 (USA, *N* = 153) [100]	Compensatory cognitive training vs enhanced supported employment	There was no significant difference between the groups in the weeks worked over the two year follow up period (Hedges g = -0.22, 95% CI: -0.54, 0.10)
		Bell 2008 (USA, *N* = 77) [96]	Neurocognitive enhancement + vocational rehabilitation vs vocational rehabilitation only	There was no significant difference between the neurocognitive enhancement intervention and control vocational rehabilitation only group in the number of hours of competitive work at 12 months from baseline (Hedges g = -0.06, 95% CI: -0.52, 0.40)
	Augmented supported employment (SE + cognitive therapy)	Lecompte 2019 (Canada, *N* = 164)	CBT for supported employment vs supported employment	There were no significant differences in the number of weeks worked at 13 months from baseline (Hedges g = 0.05, 95% CI: -0.26, 0.36)
Mixed mental health conditions	Prevocational training (Cognitive training)	Himle 2014 (USA, *N* = 58) [119]	Work-related CBT + vocational services vs vocational services only	[pilot trial] The CBT group worked more hours at both end of treatment (1 month) and 4 months post baseline
	Augmented supported employment (SE + cognitive skills training)	Bejerholm 2017 (Sweden, *N* = 63) [108]	Individual enabling and support vs TAU	The individual enabling and support group worked significantly more weeks than the TAU control group by the 12 month follow up (Hedges g = 0.68, 95% CI: 0.15, 1.22)
		Christensen 2019 (Denmark, *N* = 477) [109]	IPS with cognitive training vs TAU	Participants in the IPS group spent significantly more hours in competitive employment or education compared to the TAU control group at 18 months post baseline (Hedges g = 0.22, 95% CI: 0.04, 0.40)
		Yamaguchi 2017 (Japan, *N* = 92) [110]	Cognitive remediation + supported employment vs usual employment services	The cognitive remediation + supported employment intervention group worked significantly more average total days at 12 months compared to the control group (Hedges g = 0.68, 95% CI: 0.26, 1.10)
*Engagement in education*				
SMI	Prevocational training (with cognitive therapy)	Fowler 2019 (UK, *N* = 77) [88]	Social recovery CBT + TAU vs TAU	Only 38% of the CBT group vs 51% of the TAU group engaged in education. This difference was not significant (OR = 0.58, 95% CI 0.22, 1.56)
	Prevocational training (job related skills training)	Gutman 2009 [Education focused] (USA, *N* = 38) [91]	BRDGE supported education programme vs TAU	The Bridge participants were significantly more likely to successfully enrol in education at 6 months from baseline compared to the TAU group- 43% vs 6%, OR = 12, 95% CI: 1.33, 108.02)
		Rogers 2006 (USA, *N* = 135) [92]	Psychiatric vocational rehabilitation vs enhanced state vocational rehabilitation	The psychiatric vocational rehabilitation group did not show significantly higher odds of partaking in education at either 9 months (OR = 0.45, 95% CI: 0.08, 2.54) nor 24 months (OR = 1.45, 95% CI: 0.49, 4.33)
	Supported employment (High fidelity IPS)	Killackey 2019 (Australia, *N* = 146) [95]	IPS vs TAU	There was a significant interaction between group and time with respect to studying status, (OR = 0.87, 95% CI 0.77–0.97), after controlling for baseline study status. The odds ratio comparing studying status between the IPS and TAU groups at the 0–6-month time interval was significant, (OR = 3.04, 95% CI 1.01–9.17). No between-group differences were observed at 6–12 and 12–18 months
	Augmented supported employment (SE + job related skills training)	Nuechterlein 2019 (USA, *N* = 69)	IPS + Workplace fundamentals module vs brokered vocational rehabilitation + social skills training	IPS–WFM patients had a substantially greater likelihood of returning to school during the initial 6 months than brokered vocational rehabilitation patients (OR = 4.62, 95% CI: 1.52, 14.04), However, at 18 months there was no significant difference in the mean number of total weeks in education (Hedges g = 0.49, 95% CI: -0.04, 1.01)
Mixed mental health conditions	Augmented supported employment (SE + cognitive skills training)	Bejerholm 2017 (Sweden, *N* = 63) [108]	Individual enabling and support vs TAU	At 12-month follow-up, 15.2% of individual enabling and support participants were taking part in education, while 4% of TAU participants were (19% difference; non-significant- OR = 4.29, 95% CI:0.47, 39.27)

*N* Number of participants*, SMI* Severe mental illness*, CMD* Common mental disorder*, IPS* Individual placement and support*, TAU* Treatment as usual*, CBT* Cognitive behavioural training*, OR* Odds ratio*, CI* Confidence interval

**Table 5:** Work and education outcomes for reducing length of sickness absence in employees

Mental health diagnoses	Author	Intervention vs Control	Employer-run scheme	Individualised assessment	Self-management programme or psychological therapy offered	Graded return to work	Outcomes
*Length of sickness absence*							
CMD	Beutel 2005 (Germany, *N* = 329) [77]	Occupational training integrated into psychodynamic treatment vs TAU	Y	Y	Y	Y	The intervention group had the lowest rate of work disability both one- and two-years following discharge. The mean duration of sick leave differed significantly between the intervention group and the control group 2 years post discharge *F* = 3.08, *P* < .01
	de Weerd 2016 (Netherlands, *N* = 60) [120]	Work-focused CBT with convergence dialogue training vs work-focused CBT only	N	Y	N	N	Full return to work took longer, but not significantly so, after the end of the intervention in the convergence dialogue training intervention group compared to the control group (Hedges g = 0.64, 95% CI: -1.33, 0.05)
	Hees 2013 (Netherlands, *N* = 117) [121]	Adjuvant occupational therapy vs TAU	Y	Y	N	Y	There were no significant differences between groups in length of sickness absence at 6 months (Hedges g = 0.06, 95% CI: -0.33, 0.44), 12 months (Hedges g = 0.23, 95% CI: -0.15, 0.62) or 18 months (Hedges g = 0.12, 95% CI: -0.26, 0.50) post baseline
	Noordik 2013 (Netherlands, *N* = 160) [122]	Exposure based return to work intervention vs TAU	N	N	Y	Y	The hazard ratio at 12 months for full return to work of the intervention group compared to that of the TAU group was 0.55 (95% CI: 0.33, 0.89), indicating that they had a lower likelihood of reaching full return to work (n not reported)
	Rebergen 2009 (Netherlands, *N* = 240) [123]	Guideline based care (an activating approach, time contingent process evaluation, and cognitive behavioural principles) vs TAU	Y	N	Y	Y	At 12 months no clear effects of the intervention were found on the time to full return to work HR = 0.96, 95% CI:0.73, 1.27, *P* < .05)
	van Beurden 2017 (Netherlands, *N* = 3379) [124]	Occupational physicians intervention vs TAU	Y	Y	Y	N	At 12 months no significant differences occurred in time to full return to work between intervention and control group (HR = 0.96 95% CI: 0.85, 1.15) nor for first return to work (HR = 0.96, 95% CI:0.90, 1.15)
	Vlasveld 2013 (Netherlands, *N* = 126) [125]	Collaborative care for major depression vs TAU	Y	N	Y	N	Within 1-year follow-up, 64.6% of the collaborative care participants and 59.0% of the usual care participants had achieved lasting, full RTW (non-significant, OR = 1.27, 95% CI: 0.72, 2.25)
	Volker 2015 (Netherlands, *N* = 220) [126]	E-Health cognitive web intervention vs TAU	Y	N	Y	N	After 1 year, there was no significant difference between the E-Health and the TAU groups in the number of participants reaching full or partial return to work (OR = 1.39, 0.64, 3.01)
Mixed mental health conditions	Milligan-Saville 2017 (Australia, *N* = 1966) [127]	RESPECT manager mental health awareness training vs waitlist	N	N	N	N	There was no significant difference between the employees whose manager had received the RESPECT training and the employees whose manager was on a waitlist for the training in the proportion of employee planned hours that were spent on sick leave (b = 0.169, *p* = 0.73)

*N* Number of participants*, CMD* Common mental disorder*, TAU* Treatment as usual*,* CBT Cognitive behavioural training, *HR* Hazard ratio*, OR* Odds ratio*,* *CI* Confidence interval*, Y* Yes*, N* No

**Table 6:** Offending outcomes

Mental health diagnoses	Author (Country, included sample size)	Intervention vs Control	Court-ordered treatment	multi-disciplinary team mental health support (e.g. case management, ACT, ICM)	Specified drug and alcohol programme offered	Specified psychological therapy offered	therapeutic community (residential or daily allowance)	Outcomes
*Offending/reoffending*								
SMI	Cosden 2005 (USA, *N* = 235) [128]	Mental health treatment court vs TAU	Y	Y	Y	N	N	At 12 month follow up, a similar proportion of clients in each condition had been arrested at least once and spent some time in jail (76% in the treatment group and 72% in the treatment as usual group, OR of any convictions = 1.23, 95% CI: 0.65, 2.32) At 24 month follow up, the average number of convictions in the months since entering treatment were also not significantly different between groups (Hedges g = 0.09, 95% CI: -0.17, 0.35)
	Cusack 2010 (USA, *N* = 134) [129]	Forensic ACT vs TAU	N	Y	Y	N	N	In the first 12 months of the study, there was no difference in convictions (Hedges g = -0.14, 95% CI: -0.48, 0.20). Between 13 and 24 months into the study, the remained no significant difference in mean number of convictions (Hedges g = -0.21, 95% CI: -0.58, 0.16)
	Chandler 2006 (USA, *N* = 182) [130]	Integrated dual diagnosis treatment post-custody vs usual post-custody services	N	Y	Y	N	N	Accounting for baseline convictions, time at risk and other covariates, the difference between the percentage of control and experimental participants having any convictions was not significantly different at 18–30 month follow up when estimated in a logistic regression model (mean of 0.6 per person year vs. 0.7 per person year, z = .01, *p* = 0.989)
	Lamberti 2017 (USA, *N* = 70) [131]	Forensic ACT vs Enhanced TAU	Y	Y	N	N	N	Those patients receiving the forensic Assertive Community Treatment intervention showed fewer mean convictions than the control group after the 12-month intervention (Hedges g = 0.47, 95% CI: -0.00, 0.95, *P* = 0.05)
	Rowe 2007 (USA, *N* = 134) [132]	Group/peer support vs standard services	N	N	Y	N	Y	The intervention showed no differences to the control group for mean total charges in the past 6 months (6 months Hedges g = -0.24, 95% CI: -0.62, 0.15, 12 months Hedges g = -0.30, 95% CI:-0.68, 0.09)
	Sacks 2004 (USA, *N* = 184) [133]	Prison Modified Therapeutic Community vs Mental Health Treatment programme	N	N	N	Y	Y	12 months post-prison release, there was no significant difference in new criminal activity (47% for MTC vs 67% for MH OR = 0.70, 95% CI: 0.44, 1.12), when controlling for the outcome variable at baseline, age, age at first incarceration, employment during the year prior to baseline interview, and number of residences during the year prior to the baseline interview
	Sacks 2012 (USA, *N* = 127) [134]	Prison Modified Therapeutic Community vs Standard care	N	N	N	Y	Y	The intervention group had significantly fewer participants reincarcerated (19% vs 38%, OR = 0.39, 95% CI: 0.16, 0.97) than control participants at 12 months
Mixed mental health conditions	Kingston 2018 (Canada, *N* = 102) [135]	Reasoning and rehabilitation2 + TAU vs TAU	N	N	N	Y	N	There was no significant difference between groups in the odds of not being convicted or arrested in the 18 months since baseline (OR of no recidivism = 0.55, 95% CI: 0.25, 1.21)

*N* Number of participants, *SMI* Severe mental illness, *TAU* Treatment as usual, *CBT* Cognitive behavioural training, *ACT* Assertive Community Treatment, *OR* Odds ratio, *CI* Confidence interval, *Y* Yes, *N* No

**Table 7:** Rights, inclusion and citizenship outcomes

Mental health diagnoses	Author	Intervention vs Control	Outcomes
*Inclusion*			
Mixed mental health conditions	Salzer 2016 (USA, *N* = 100) [136]	Peer-delivered core services of Centres for Independent Living (CILs) vs TAU	There were no differences between the CIL and control condition over time on total number of participation days F (92,172) = 1.86, *P* = .16)
SMI	Segal 2010 (USA, *N* = 505) [137]	Self-help agencies and community mental health agency services, vs community mental health agency services (CMHA) only	Combined self-help agencies and community mental health services were significantly better able to promote recovery of client-members than CMHA services alone. The combined intervention sample showed greater improvements in independent social integration (social presence, access, participation, production, employment and consumption behaviours) (*F* = 12.13, df = 3 and 491, *P* < .001)
*Difficulties with access to services*			
Mixed mental health conditions	Salzer 2016 (USA, *N* = 100) [136]	Peer-delivered core services of Centres for Independent Living (CILs) vs TAU	Time x Group interactions in repeated measures ANOVA showed no significant differences between the intervention and control condition over time on total number of unmet needs (*F*(2, 172) = 1.60, *P* = 0.21)

*N* Number of participants*, TAU* Treatment as usual

### Patient and public involvement

Co-authors TK, PS and KM, researchers with relevant lived experience of using mental health services and/or supporting others who do so, were members of the review working group and contributed to review design, interpretation of results and writing the paper. They have also provided a commentary which highlights key issues arising from the review from a perspective of people with lived experience of mental health conditions, which accompanies this paper.

## Results

The systematic review search returned a total of 11,810 records, from which 212 potentially relevant full text systematic reviews were identified. From this search, we included one fully relevant review [7] in the employment domain, and searched the inclusion lists of 51 partially relevant systematic reviews for RCTs meeting our inclusion criteria. We carried forward an additional 19 RCTs through this process. The RCT search across all eight social domains returned a total of 20,320 records. From this, 388 potentially relevant full-text articles were identified. Of these, 80 RCTs were included. A forward citation search of all included RCTs retrieved an additional three RCTs for inclusion. The wholly relevant systematic review [7] also included 48 RCTs- these were not extracted individually and instead the results of the review were summarised alongside additional RCTs in the same domain. This review included 8743 participants with severe mental illness.

In addition to this review, we included a total of 102 RCTs with 32,497 participants. Seventy-one trials focused on patients with SMI, and 16 on patients with CMD. Fifteen did not specify the diagnoses of their participants and/or included mixed diagnoses. Characteristics of included studies are shown in Table 8.

**Table 8:** Study characteristics

Study ID	Domain (s)	Publication type	Number of Groups	Control Type	Total Sample Size	Population diagnosis	Country	Mean Sample Age (range)	Sample Gender (% female)	Sample Ethnicities	Intervention Setting	Additional publications
Siuijkerbuijk 2017 [7]	Employment	Systematic review	NA	Treatment as usual, active control	8743	Severe mental illness (100%)	Multiple	36 (NR)	36%	NR	NA	
Aubry 2016 [58]	Housing	RCT	2	Treatment as usual	950	Major depressive episode (43%), Mania or hypomanic episode (16%), Mood disorder with psychotic features (20%), Post-traumatic stress disorder (27%), Panic disorder (21%), Psychotic disorder (52%), Substance-related problems (73%)	Canada	39.4 (NR)	32%	Member of racial or ethnic minority group (21%), Aboriginal (19%)	Residential	Aubry 2015 [138]
Bejerholm 2017 [108]	Employment	RCT	2	Treatment as usual	63	Depression (69%), Bipolar (31%)	Sweden	41 (NR)	72%	Native (92%), Immigrant (8%)	Outpatient	
Bell 1993 [82]	Employment	RCT	2	Active	100	Schizophrenia (100%)	USA	40 (NR)	6%	White (76%), Black (21%), Hispanic (3%)	Veterans affairs medical centre	
Bell 2003 [115]	Employment	RCT	2	Active	63	Schizophrenia (100%)	USA	44 (NR)	0	Caucasian (62%), African American (30%), Hispanic (8%)	Veterans affairs medical centre	
Bell 2005 [83]	Employment	RCT	2	Active	145	Schizophrenia (100%)	USA	42.8 (NR)	41%	Caucasian (46%), African American (23%), Hispanic (2%), Asian (2%)	Veterans affairs medical centre	
Bell 2008 [96]	Employment	RCT	2	Active	77	Schizophrenia (100%)	USA	40 (NR)	46%	Caucasian (47%), African American (23%), Hispanic (4%), Asian (1%)	Community	
Bell 2018 [84]	Employment	RCT	2	Active	77	Schizoaffective disorder (13.2%), Schizophrenia (36.8%), Other disorder (50%)	USA	51.2 (NR)	10.40%	Not Hispanic or Latino (93.5%), Hispanic/Latino (4%), Unknown or NR (2.5%)	Cognitive training lab	
Beutel 2005 [77]	Employment	RCT	2	Treatment as usual	329	Affective disorders (36%) Adjustment disorders (19%), Anxiety disorders (11%), Other disorders (16%), Other neurotic (3%), Somatoform disorders (12%)	Germany	38 (19–50)	58.30%	NR	Inpatient	
Boevink 2016 [48]	Social Isolation	RCT	2	Treatment as usual	163	Affective disorder (11.7%), Non-affective psychotic disorder (40.5%), Personality disorder (14.7%), Other (33.1%)	Netherlands	43.9 (NR)	49.10%	NR	Community and residential	
Burnam 1996 [62]	Housing	RCT	3	No intervention	276	Comorbid schizophrenia + major affective disorder (38%), Major affective disorder (55%), Schizophrenia disorder (7%)	USA	37 (NR)	16%	White (58%), Black (28%), Other (14%)	Community and residential	
Castelein 2008 [49]	Social Isolation	RCT	2	Treatment as usual	106	Other psychotic disorders (25.5%), Schizophrenia (79%)	Netherlands	38.6 (NR)	31.10%	NR	Community	
Chandler 2006 [130]	Offending	RCT	2	Treatment as usual	182	Major depressive or other depressive disorder (28.8%), Schizophrenia (22%), Schizoaffective disorder (5.5%), Bipolar disorder (10.4%), Psychotic disorder NOS (28%), Other including PTSD and other anxiety disorders (8.2%)	USA	NR	19.20%	African American (66.4%), White (21.4%), Hispanic (3.3%), Other (3.3%)	Community	
Christensen 2019 [109]	Employment	RCT	3 (2 included)	Treatment as usual	477	Schizophrenia spectrum disorders (77%), Bipolar disorder (11.5%), Recurrent depression (11.5%)	Denmark	32.9 (NR)	38.16%	NR	Outpatient	
Conoley 1985 [39]	Social Isolation	RCT	3 (2 included)	Wait-list	38	Depression (100%)	USA	NR	100%	NR	Community	
Cook 2008 [93]	Employment	RCT	2	Treatment as usual	1273	Schizophrenia (33%), Schizoaffective disorder (18%), Major depression (24%), Bipolar disorder (16%), Dysthymia (3%)	USA	38.5 (NR)	53%	White (50%), Other (50%)	Community and residential	
Cosden 2005 [128]	Offending	RCT	2	Treatment as usual	235	Mood disorder (35.5%), Schizophrenia (32.5%), Bipolar Disorder (22.2%), Other (10.3%)—Dual-diagnosis (83.3%)	USA	NR	50.60%	European-American (70.6%), Hispanic (17.4%), African American (7.7%), Other (4.3%)	Community	Cosden 2003 [139]
Cusack 2010 [129]	Offending	RCT	2	Treatment as usual	134	Psychotic disorder (65%), Schizoaffective disorder (26.9%)	USA	36.8 (NR)	41%	Caucasian (63%), Hispanic (21.6%), African American (8.2%)	Community	
Davidson 2004 [46]	Social Isolation	RCT	3	No intervention	260	Psychotic disorder (50%), Affective disorder (34%), Anxiety disorder (2%), Other Axis I disorder (1%), Unknown (12%), Co-occuring substance use disorder (44%)	USA	NR	57%	White (82%), African American (11%), Hispanic/Latino (2%), Asian/Pacific Islander (1%)	Community	
Davis 2012 [80]	Employment	RCT	2	Active	85	Post-traumatic stress disorder (100%)	USA	40.2 (NR)	12%	African American (71.5%), Caucasian (27%), Native American (1%)	Community	
Davis 2015 [116]	Employment	Feasibility RCT	2	Active	34	Schizophrenia (58.8%), Schizoaffective (41.2%)	USA	51.7 (NR)	3%	African American (62.3%), White (37.7%)	Outpatient	
Davis 2018 [81]	Employment	RCT	2	Active	541	Post-traumatic stress disorder (100%)	USA	42.2 (NR)	49.50%	Hispanic (66.5%), White (50.7%), African American (41.6%), Other (33.3%)	Outpatient	
De Waal 2019 [140]	Victimisation	RCT	2	Treatment as usual	250	Psychotic disorder (38%), Mood disorder (21.6%), Post-traumatic stress disorder (13.2%), Anxiety disorder (8%), Attention-deficit/hyperactivity disorder (8%), Personality disorder (36%), Intellectual disability (12.4%), Other disorder (11.2%)	Netherlands	42.1 (NR)	29.60%	Dutch (72.3%), Other (7.6%), Surinamese (6.4%), European (6%), Moroccan (4.4%), Dutch Antilles (2.4%)	Outpatient and inpatient	
De Weerd 2016 [120]	Employment	RCT	2	Active	60	Somatoform disorder (56.7%), Depressive disorder (23.3%), Anxiety Disorder (20%)	Netherlands	39.9 (NR)	46.67%	NR	Outpatient	
Elbogen 2016 [141]	Money	RCT	2	Treatment as usual	184	Mixed (100%)	USA	NR (18–65)	NR	NR	NR	
Ellison 2020 [63]	Housing	RCT	2	Treatment as usual	166	Dual-diagnosis serious mental disorder + substance abuse (100%)	USA	52.8 (NR)	7.23%	White (50%), African American (50%)	Community and residential	
Erickson 2020 [94]	Employment	RCT	2	Treatment as usual	109	Schizophrenia spectrum (50.5%), Bipolar (14.7%), Major depression (9.2%), Psychosis NOS (15.6%), Other (6.4%)	Canada	23.5 (NR)	17.40%	NR	Outpatient	
Fletcher 2008 [64]	Housing	RCT	3 (2 included)	Treatment as usual	191	Dual-diagnosis serious mental disorder + substance abuse (100%)	USA	NR (18–66)	NR	NR	Community	
Fowler 2019 [88]	Employment	RCT	2	Treatment as usual	77	Psychosis (100%)	United Kingdom	29 (18–52)	29%	White (85.7%)	Community	
Gelkopf 1994 [44]	Social Isolation	RCT	2	Active	34	Schizophrenia (100%)	Israel	45.4 (NR)	17.60%	NR	Inpatient	
Glynn 2004 [54]	Social Isolation	RCT	2	Active	63	Schizophrenia (100%)	USA	43.5 (18–60)	8%	Caucasian (44%), African American (40%), Hispanic (13%), Asian (3%)	Outpatient	
Glynn 2017 [101]	Employment	RCT	2	Active	67	Schizophrenia (100%)	USA	41 (18–65)	16%	White (76%), Black (15%), Other (4%), Asian (3%), Latino (1%)	Community	
Goldfinger 1999 [65]	Housing	RCT	2	Treatment as usual	118	Schizophrenia (45%), Schizoaffective disorder (17%), Bipolar disorder (14%), Major depression (13%), Other (11%)	USA	38 (NR)	28%	African American (41%)	Community and residential	Dickey 1997 [142]
Granholm 2005 [57]	Social Isolation	RCT	2	Treatment as usual	76	Schizophrenia (100%)	USA	NR (42–74)	26.40%	Caucasian (79%)	Community	
Gutman 2009 [91]	Employment	RCT	2	Treatment as usual	38	Schizophrenia (42%), Schizoaffective disorder (29%), Bipolar disorder (16%), depression (13%)	USA	NR (19–55)	42%	Hispanic (39%), African American (37%), White (21%)	Community	
Harris 2017 [118]	Employment	RCT	2	Treatment as usual	86	Schizophrenia (58.1%), Schizoaffective (5.8%), Bipolar (31.4%), Other psychotic (4.7%)	Australia	39.6 (NR)	36.05%	NR	Outpatient	
Haslam 2019 [40]	Social Isolation	RCT	2	Treatment as usual	120	Major depression (41.7%), Anxiety disorder (38%), Post-traumatic stress disorder (7.5%), Others (12.5%), None (but with symptoms meeting criteria for major depression) (40.8%)	Australia	31.1 (17–69)	64%	Caucasian (74%), Australian (69%)	Outpatient	
Hasson-Ohayon 2014 [42]	Social Isolation	RCT	2	Active	55	Serious mental illness (100%)	Israel	38.5 (21–62)	44%	NR	Community	
Hees 2013 [121]	Employment	RCT	2	Treatment as usual	117	Major depression (100%)	The Netherlands	43 (18–65)	53%	NR	Outpatient and community	
Hellstrom 2017 [79]	Employment	RCT	2	Treatment as usual	326	Depression (69%), Phobic anxiety (7.7%), Other anxiety (12%), Bipolar disorder (11.3%)	Denmark	35 (18–60)	68%	NR	Community	
Henderson 2013 [104]	Employment	Feasibility RCT	2	Treatment as usual	80	Schizophrenia spectrum (30%), Depression (12.7%), Bipolar disorder (16.5%), Personality disorders (5.1%), Anxiety (7.6%), Anxiety and depression (7.6%), Mixed (6.3%)	United Kingdom	36.1 (NR)	48%	Black/Black British (47%), White (38%), Other (11%), Asian/Asian British (4%)	Community	
Herman 2011 [66]	Housing	RCT	2	Treatment as usual	150	Schizophrenia (61%), Schizoaffective (35%), Other (4%)	USA	37.5 (NR)	29%	African American (62%), White (17%), Latino (15%), Other (6%)	Community	Baumgartner 2012 [143]
Himle 2014 [119]	Employment	Pilot RCT	2	Treatment as usual	58	Depression (60.3%), Post-traumatic stress disorder (37.9%), Generalized anxiety disorder (19%), Obsessive–compulsive disorder (15.5%), Panic disorder (13.8%), Bipolar disorder (3.4%), Psychotic disorder (3.4%), Specific phobia (1.7%)	USA	43.6 (NR)	32.80%	African American (86.2%), White (10.34%), Multiracial (3.45%)	Community	
Hurlburt 1996 [67]	Housing	RCT	4	No intervention	361	Schizophrenia (55%), Other (bipolar disorder or major depression (55%))	USA	NR (NR)	33.20%	White (62.8%), Black (19.7%), Hispanic (12.5%), Other (5%)	Community and residential	
Kern 2018 [102]	Employment	RCT	2	Active	58	Schizophrenia/schizoaffective disorder (100%)	USA	42.7 (NR)	15.52%	White (65.3%)	Community	
Killackey 2019 [95]	Employment	RCT	2	Treatment as usual	146	Psychosis (100%)	Australia	20.4 (NR)	30.80%	Australian (76%)	Community	Killackey 2012/Killackey 2013/Killackey 2014 [144–146]
Kingston 2018 [135]	Offending	RCT	2	Treatment as usual	101	Major mood, psychotic, anxiety and/or trauma-related disorder (100%)	Canada	34.5 (NR)	0	Caucasian (87.6%), Aboriginal (11.3%), Other (Asian) (9.3%), Black (6.2%)	Prison and community	
Korr Joseph 1995 [68]	Housing	RCT	2	Treatment as usual	95	Schizophrenia, depressive and affective disorders, or other psychoses (78%), Other diagnosis (22%)	USA	38.09 (NR)	20.00%	Black (43%), White (49%), Other (8%)	Community and housing	
Kukla 2018 [112]	Employment	RCT	3 (2 included)	Active	50	Schizophrenia (74%), Schizoaffective disorder (26%)	USA	51.5 (NR)	4.00%	African American (58%), White (40%), Hispanic American (2%)	Outpatient	
Lamberti 2017 [131]	Offending	RCT	2	Treatment as usual	70	Schizophrenia (51%), Depression with psychotic features (19%), Psychotic disorder, NOS (10%), Schizoaffective disorder (11%), Bipolar disorder with psychotic features (9%)	USA	37.5 (NR)	39%	African American (73%), Caucasian (19%), Hispanic (8%)	Outpatient	
Lecomte 2020 [147]	Employment	RCT	2	Active	164	Mood disorder (18.5%), Anxiety disorder (7.4%), Organic disorder (0.6%), Psychotic disorder (58.6%), Substance-related (1.2%), Personality disorder (6.2%), Developmental disorder (1.9%), Other (5.6%)	Canada	36.6 (NR)	39.30%	Caucasian (63.4%)	NR	
Lehman 1997 [69]	Housing	RCT	2	Treatment as usual	152	Schizophrenia (44.5%), Schizoaffective disorder (14%), Bipolar (20.5%), Depression (8.5%), Other (12.6%)	USA	37.5 (NR)	32.50%	African American (72.3%), White (23.7%)	Community	
Lindenmayer 2008 [89]	Employment	RCT	2	Attentional control	85	Schizophrenia or schizoaffective disorder (84%); "other" diagnosis (17%) [details of mental health conditions of participants with diagnoses falling into the "other" category were not reported]	USA	43.5 (NR)	11%	Black (58%), Hispanic (27%), White (13%), Asian (3%)	Inpatient	
Lipton 1988 [70]	Housing	RCT	2	Treatment as usual	49	Schizophrenia (81%), Personality disorders (8%), Affective disorders (2%), Other (8.1%)	USA	37 (NR)	35%	NR	Housing	
Lloyd-evans 2020 [41]	Social Isolation	Feasibility RCT	2	Treatment as usual	211	Common mental disorders (100%)	UK	43.1 (NR)	73%	White (64%), Black/African/Caribbean/Black British (13%), Asian/Asian British (10%), Mixed/multiple ethnic groups (8%), Other ethnic groups (5%)	Outpatient and community	
Lysaker 2005 [113]	Employment	RCT	2	Treatment as usual	50	Schizophrenia (74%), Schizoaffective disorder (26%)	USA	48.1 (NR)	0	African American (56%), Caucasian (42%), Latino (2%)	Outpatient and community	
Marder 1996 [55]	Social Isolation	RCT	2	Active	80	Schizophrenia (100%)	USA	38.2 (NR)	0	Non-white (68.8%)	Veterans affairs medical centre	
McGurk 2007 [97]	Employment	RCT	2	Treatment as usual	44	Schizophrenia (23.4%), Schizoaffective disorder (22.4%), Bipolar disorder (23.4%), Major depression (16.8%), Other (14%)	USA	35.59 (NR)	45.50%	African American (68.2%), Hispanic (15.9%), Caucasian (13.6%), Asian (2.27%)	Community	McGurk 2005 [148]
McGurk 2015 [98]	Employment	RCT	2	Active	107	Schizophrenia (23.4%), Schizoaffective disorder (22.4%), Bipolar (23.4%), Depression (16.8%), Other (14%)	USA	44.1 (NR)	34.60%	White (86%), Black (10.3%), Asian (1.9%), More than one race (1.9%)	Community	
McGurk 2016 [85]	Employment	RCT	2	Active	54	Schizophrenia (83.3%), Major mood disorder (13%), Other (3.7%)	USA	37.7 (NR)	29.63%	African American (61.1%), Caucasian (25.9%), Hispanic/Latino (18.5%), Multiracial (14.8%)	Community	
McHugo 2004 [71]	Housing	RCT	2	Active	121	Schizophrenia (72.7%), Mood disorder (27.3%)	USA	39.85 (21–60)	53%	African American (82.6%)	Community	
Mervis 2017 [86]	Employment	RCT	2	Active	64	Schizoaffective disorder (46.9%), Other (43.1%)	USA	36.1 (NR)	39.10%	NR	Community	
Milligan-Saville 2017 [127]	Employment	Cluster RCT	2	Waitlist	88 (managers) 1966 (employees)	NR	Australia	(managers) 49.3 (NR)	0%	NR	Workplace	
Morse 1992 [72]	Housing	RCT	3 (2 included)	Treatment as usual	116	Schizophrenia, Depression, Bipolar, Other psychotic disorders, Other disorders not listed (100%)	USA	NR	NR	NR	Community	
Morse 1997 [76]	Housing	RCT	3	Active	165	Schizophrenia (66%), Recurrent depression (15%), Bipolar disorder (13%), Atypical psychosis (12%), Delusional or paranoid disorder (3%), Dementia (1.2%)	USA	34.7 (NR)	42%	African American (55%), Caucasian (45%)	Community	
Morse 2006 [73]	Housing	RCT	3 (2 included)	Treatment as usual	NR	Schizophrenia, Schizoaffective disorder, Atypical psychotic disorder, Bipolar disorder, Major depression-recurrent disorder, Delusional disorder + One or more substance use disorders (100%)	USA	NR	NR	NR	Community	
Mueser 2005 [117]	Employment	RCT	2	Active	35	Schizophrenia or schizoaffective disorder (66%), Major depression or bipolar disorder (11%), Other psychiatric diagnoses (23%)	USA	37.7 (NR)	20%	Non-hispanic White (97%), Asian (3%)	Outpatient	
Noordik 2013 [122]	Employment	RCT	2	Treatment as usual	160	Stress-related disorders (22.5%), Depressive disorders (23.1%), Anxiety disorder (23.1%), Mixed anxiety-depressive disorders (29.4%)	Netherlands	45.4 (NR)	70%	NR	Community	
Nuechterlein 2019 [103]	Employment	RCT	2	Active	69	Schizophrenia or schizoaffective disorder (100%)	USA	24.7 (NR)	33.30%	Multiracial (37.7%), White (26.1%), Hispanic (26.1%), Asian (11.6%), Pacific Islander (2.9%)	Outpatient	
Okpaku 1997 [105]	Employment	RCT	2	Treatment as usual	152	Mood disorders and Schizophrenia (67%), Psychotic disorder (44%), Mood disorder (36%)	USA	36.8 (NR)	41%	White (60%)	Community	
Overland 2018 [111]	Employment	RCT	2	Treatment as usual	1193	Common mental disorders (100%)	Norway	40.4 (NR)	67%	NR	NR	
Pos 2019 [51]	Social Isolation	RCT	2	Treatment as usual	99	Schizophrenia (100%)	Netherlands	25.4 (NR)	19%	Member of a ethnic minority group (54.5%)	Outpatient	
Pot-Kolder 2018 [52]	Social Isolation	RCT	2	Treatment as usual	116	Schizophrenia or schizoaffective disorder (100%)	Netherlands	38 (NR)	29.30%	Non-dutch origin (34.5%)	Outpatient	Pot-Kolder 2020 [149]
Priebe 2020 [56]	Social isolation	RCT	2	Active	124	Schizophrenia or schizoaffective disorder (100%)	UK	42.4 (NR)	34.70%	Black African (19.4%), Bangladeshi (18.5%), Black Caribbean (17.7%), White (15.3%), Other unspecified (8.9%), Black other (5.6%), Asian other (4.03%), Indian/Pakistani (4%), Mixed/multiple ethnic groups (2.4%), White other (2.4%)	Community	
Rebergen 2009 [123]	Employment	RCT	2	Treatment as usual	240	Mixed (100%)	The Netherlands	39.38 (NR)	44.00%	NR	Outpatient	Rebergen 2009b [150]
Reme 2019 [106]	Employment	RCT	2	Treatment as usual	410	Psychotic (19.8%), Bipolar (13.9%), Major depression (40%), Anxiety (40.5%), Other (26.6%)	Norway	35 (NR)	48.60%	Norwegian (86.1%)	Outpatient	
Rivera 2007 [50]	Social Isolation	RCT	3 (2 included)	Treatment as usual	203	Schizophrenia (29%), Schizoaffective disorder (20%), Other psychotic disorder (3%), Bipolar disorder (26%), Depression (22%), Other or data missing (1%)	USA	38.6 (NR)	48.50%	Caucasian (30.2%), Hispanic (29.4%), Other (22.1%), African American (18.4%)	Community	
Roberts 2014 [53]	Social Isolation	RCT	2	Treatment as usual	66	Schizophrenia (42.4%), Schizoaffective (56.1%), Psychosis NOS (1.5%)	USA	39.7 (NR)	33.30%	Caucasian (63.6%), African American (36.4%), Hispanic (6.1%)	Outpatient	
Rodriguez Pulido 2019 [99]	Employment	RCT	2	Active	57	Schizophrenia (74.5%), Bipolar disorder (14.9%), Personality disorder (8.5%), Depression (2.1%)	Spain	40.5 (NR)	31.92%	NR	Community	
Rogers 2006 [92]	Employment	RCT	2	Treatment as usual	135	Severe mental illness (100%)	USA	33.9 (NR)	45.90%	Caucasian (58%), African American (30.3%), Other (including multiracial) (11.7%)	NR	
Rossler 2020 [107]	Employment	RCT	3 (2 included)	Active	78	Mental and behavioural disorders due to psychoactive substance use (11.5%), Schizophrenia and similar disorders (8.97%), Mood disorders (44.87%), Anxiety, dissociative, stress related and somatoform disorders (15.38%), Personality disorders (12.82%)	Switzerland	36.05 (NR)	51.28%	NR	Outpatient	Rossler 2018 [151]
Rowe 2007 [132]	Offending	RCT	2	Treatment as usual	114	Psychotic disorder (41.3%), Major mood disorder (44.2%), Other (19.2%)	USA	39.8 (NR)	32%	African American (58%), White (31%), Hispanic (15%), Other (9%), Native American (3%)	Community	
Russinova 2018 [90]	Employment	RCT	2	Wait-list	51	Schizophrenia/schizoaffective (31.4%), Bipolar (31.4%), Depression (33.3%), Other (5.9%)	USA	46.2 (NR)	60.80%	Non-Hispanic white (64.7%), Member of minority ethnic groups (35.3%)	NR	
Sacks 2004 [133]	Offending	RCT	2	Active	185	Dual-diagnosis serious mental disorder + substance abuse (100%)	USA	34.3 (NR)	0	Caucasian (49%), Black (30%), Hispanic (16.5%), Other (4%)	Prison and community	
Sacks 2012 [134]	Offending	RCT	2	Active	127	Dual-diagnosis serious mental disorder + substance abuse (100%)	USA	38.2 (NR)	0	White (56%), Hispanic (17%), Other/mixed (17%), Black (10%)	Prison and community	
Salzer 2016 [136]	Rights inclusion and citizenship	RCT	2	Treatment as usual	100	Schizophrenia-spectrum or affective disorder (100%)	USA	48.7 (NR)	46.50%	Black (74.8%), White (21.2%), Latin/Hispanic (4.04%), Native American (2%), Other race (2%), Asian (1%)	Community	
Sanches 2020 [114]	Employment	RCT	2	Active	188	Psychotic disorder (60.1%), Bipolar disorder (3.2%), Depressive or anxiety disorder (6.9%), Personality disorder (6.4%), Eating disorder (6.9%), Other (16.5%)	Netherlands	39.9 (NR)	42%	NR	Outpatient	
Schene 2007 [78]	Employment	RCT	2	Treatment as usual	62	Major depression (100%)	The Netherlands	45.9 (NR)	51.60%	NR	Outpatient	
Segal 2010 [137]	Rights inclusion and citizenship	RCT	2	Treatment as usual	505	Schizophrenia or schizoaffective disorder (9%), Major depression (76%), Other (15%)	USA	NR	47%	White (36%), African American (34%), Other (30%)	Community	
Sheridan 2015 [47]	Social Isolation	RCT	2	Active	107	Diagnosis of enduring mental illness (100%)	Ireland	51 (NR)	52.30%	NR	Community	
Shern 2000 [74]	Housing	RCT	2	Treatment as usual	168	Serious mental disorders (100%)	USA	39.97 (21–66)	34%	Black (61%), White (29%), Hispanic (10%)	Community	
Silverman 2014 [43]	Social Isolation	RCT	4 (2 included)	Attentional control	45	Bipolar Disorder (35.6%), Major Depressive Disorder (48.9%), Schizoaffective disorder (2.3%), Schizophrenia (2.3%), Other (2.3%) No response: 8.89%	USA	35.55 (NR)	51.10%	Caucasian American (71.1%), African American (13.3%), Other (8.9%), Hispanic American (4.4%)	Inpatient	
Stergiopoulos 2015 [60]	Housing	RCT	2	Treatment as usual	1198	Depression (59%), Mania/hypomania (9.9%), Post-traumatic stress disorder (31.3%), Panic disorder (25.1%), Mood disorder with psychotic features (13.2%), Psychotic disorder (21.6%)	Canada	42.2 (NR)	32.60%	White (48.4%), Ethnoracial (27.9%), Aboriginal (23.7%)	Community and residential	
Susser 1997 [75]	Housing	RCT	2	Treatment as usual	96	Schizohrenia (56.1%), Other (33.9%)	USA	NR	0	African American (72.4%), Other (17.6%)	Community	Jones 2003 [152]
Terzian 2013 [45]	Social Isolation	RCT	2	Treatment as usual	357	Schizophrenia (100%)	Italy	NR (18–45)	31%	NR	Community	
Tinland 2020 [59]	Housing	RCT	2	Treatment as usual	703	Schizophrenia (69.3%), Bipolar (30.7%)	France	38.8 (NR)	17.50%	French (81.8%), Other (13.5%)	NR	
Tsemberis 2004 [61]	Housing	RCT	2	Treatment as usual	206	Psychotic (53%), Mood-depressive (14%), Mood-bipolar (14%), Other (5%)	USA	NR	21%	Black (41%), White (27%), Mixed/other/unknown (18%), Hispanic (15%)	Housing	Gulcur 2003 [153]
Twamley 2019 [100]	Employment	RCT	2	Active	153	Schizophrenia or schizoaffective disorder (38%), Bipolar disorder (24%), major depressive disorder (38%)	USA	43.7 (NR)	43.10%	Racial/ethnic minority (37.9%)	NR	Twamley 2008/Twamley 2012 [154, 155]
Van Beurden 2017 [124]	Employment	RCT	2	Treatment as usual	3379	Common mental disorders (100%)	Netherlands	44.6 (NR)	58.50%	NR	Outpatient	
Vauth [87] 2005	Employment	RCT	3 (2 included)	Treatment as usual	93	Schizophrenia (100%)	Germany	28.95 (NR)	38.71%	NR	Inpatient	
Vlasveld 2013 [125]	Employment	RCT	2	Treatment as usual	126	Moderate major depressive disorder (100%)	Netherlands	42.6 (NR)	53.80%	Dutch (93.66%)	Community	
Volker 2015 [126]	Employment	RCT	2	Treatment as usual	220	Common mental disorders (100%)	Netherlands	44.2 (NR)	59.10%	Dutch (97.7%)	Online	Lokman 2017 [156]
Yamaguchi 2017 [110]	Employment	RCT	2	Treatment as usual	92	Schizophrenia (87%), Depression (7.6%), Bipolar (5.4%)	Japan	34.7 (NR)	38.04%	NR	Community and inpatient	

*Note: NR* Not recorded*, RCT* Randomised controlled trial

### Interventions

Interventions were often complex in nature and included multiple treatment components. A full description of interventions from all included studies is available in Additional File 3, while characterisation of key components is presented alongside outcomes in Tables 3, 4, 5, 2, 6, 7.

### Study quality

**Fig. 2:**
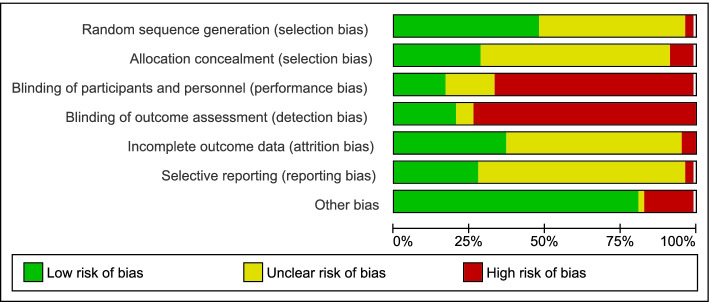
Risk of bias graph: review authors' assessments about each risk of bias item presented as percentages across all included studies

The quality of the included systematic review was deemed to be high, with all requirements of the AMSTAR 2 tool being met. The quality of RCTs varied across different domains of the Cochrane Risk of Bias (ROB) tool. Fifteen RCTS were of low ROB in the majority (5/7) of domains, though only two RCTs [64, 127] were rated as low ROB across six domains. The most common areas of bias were blinding of participants and assessments. In general ratings of high ROB resulted from aspects necessary due to the populations, target problems and RCT designs of studies: areas of most concern were blinding of outcome assessment and also of participants. Attrition bias was frequently unclear, usually due to high rates of attrition that spanned all study arms. Reporting bias was also often unclear due to a lack of study protocol publication. A summary of Risk of Bias rating is available in Fig. 2 and a summary of variations in quality across social domains and our evaluation of the included syis available in Additional File 4.

### Housing and homelessness

Nineteen of the included trials, including 5281 participants, focused on homelessness. Of these, 18 studies included participants with SMI, including three requiring dual diagnosis of SMI and substance use disorder, and one including participants with mixed or unspecified diagnoses. All 19 of these studies reported on outcomes relating to achieving or sustaining housing, and three also reported on housing quality outcomes. The largest trials made use of the “Housing First” programme. This is based on the principle that housing is a fundamental right, and therefore provides immediate access to independent housing (with no requirement to progress through staged supported housing first) as well as mental health care to homeless people with mental health conditions [157]. Housing First interventions blend components of housing support and Assertive Community Treatment. Other interventions encompassed assertive outreach, specified psychological therapies, and supported housing. Table 3 shows the results of each study and key components of interventions.

#### Achieving and sustaining housing

##### **SMI populations (*N* = 18 trials)**

Four studies reported specifically on Housing First interventions [58–61]. Several additional studies used sub-samples from Aubry et al. [58] and Stergiopoulos et al. [60]: these were not synthesised due to overlapping study populations but are listed in Additional File 5. Housing First interventions tended to be integrated with case management [58–60]. Results of these studies suggest that Housing First programmes result in significant improvements in achievement and retention of stable housing (remaining housed) at both short- and long-term follow-up, while Tsemberis [61] reported that Housing First participants experienced a faster increase in stable housing compared to continuum of care control participants (a programme subscribing to the abstinence–sobriety belief that without strict adherence to treatment and sobriety, housing stability is not possible). Interventions involving supported housing or on-site staff tended to be described in earlier publications [62, 65, 71, 74, 158]. Though results of two studies suggested that supported housing interventions did not increase the chances of being stably housed after the intervention when compared to independent housing controls [62, 65], there were mixed findings overall in the likelihood of participants being stably housed across the five studies (see Table 4). Mental health support using multi-disciplinary teams was a common element described in housing interventions, with an additional 10 studies alongside Housing First interventions including some aspect of this. While half of these reported significant benefits of their interventions compared to controls [66, 69–71, 73], other studies including this aspect did not report significant benefits [62, 64, 65, 67, 72]. Housing support workers (outside of multi-disciplinary team support) were included in three trials [74, 75, 158] and governmental monetary support was included in one trial which examined the benefits of Sect. 8 subsidies (subsidised rent, with remaining amount due to private landlords paid for by the housing authority) [67]. Having support with practicalities did seem to contribute to increased numbers of participants achieving stable housing compared to Treatment as usual (TAU) controls. Manualised psychological therapy was reported in three studies [63, 64, 73]. These three studies focused on recovery of substance abuse and community integration alongside Cognitive behavioural therapy (CBT), and were the three studies including participants with a dual diagnosis of substance abuse and SMI. It is unclear if psychological therapy of this kind significantly adds to improvements in housing in this population.

##### **Mixed populations (*N* = 1 trial)**

One study [76] reported on an intervention for participants with mixed mental health conditions, comparing broker case management (where primarily office-based case managers developed individualised service plans for clients), Assertive Community Treatment (ACT) with additional community workers (where workers conducted more homeless outreach and engagement methods than broker case management), and ACT only. In contrast to author hypotheses, participants in the ACT only group averaged more days in stable housing at 18 months than the other conditions.

#### Housing quality

##### **SMI populations (*N* = 3 trials)**

Three studies reported housing quality outcomes. One Housing First study [58] reported that housing quality was rated as significantly higher in the Housing First group at all follow-ups (6–24 months). This, and the remaining two interventions [69, 70] included multi-disciplinary team elements. There was some limited evidence that multi-disciplinary team elements were associated with reports of better housing quality [58, 70], though this may only be short lived [69].

#### Housing: summary

Overall, the majority of studies using a range of strategies reported substantial effects for interventions on achieving stable housing and better housing quality for homeless participants. Only five of 19 interventions did not find significant improvements compared to the control group. Housing First interventions can provide long term (up to 24 months) benefits for homeless participants with severe mental illness for housing related outcomes, though it is currently not clear whether multi-disciplinary teams, involved in Housing First protocols as well as other intervention strategies have significant additional benefits on housing outcomes. Supported housing, less widely studied recently, does not appear to show clear benefits in achieving stable housing. A small number of trials report on other forms of practical support, finding some benefits.

### Money and basic needs

We found only one RCT aiming to improve money management in people with a range of psychological conditions [141]. This study from the USA included 184 participants and compared a psychoeducational money management programme ($afe budget) to TAU. There were no significant main effects of the intervention on outcomes in the randomized clinical trial.

### Work and education

#### Obtaining and retaining paid employment and enrolling in education

Studies which targeted work and education often made use of the “Individual Placement and Support” (IPS) programme (with and without augmentation), in which employment specialists embedded in clinical teams aim to support participants who would like to work in a rapid search for competitive employment, and then provide time-unlimited and individualised support to participants and employers [159]. A range of other interventions such as other supported employment (SE) protocols, skills training and transitional employment were also examined in the literature.

We identified a high quality Cochrane review of 48 RCT publications evaluating interventions for obtaining and maintaining employment in adults with SMI [7]. The review identified trials of SE (including those specifically described as IPS, and others which described a more general approach to SE, *n* = 30), SE augmented with other interventions (including both symptom skills training, such as cognitive strategies or mindfulness-based exercise, and job-related training such as decision-making training, *n* = 13), prevocational training (*n* = 17) and transitional employment (*n* = 6), with some studies comparing multiple interventions. A network meta-analysis was conducted to identify which interventions are more effective in helping unemployed adults with SMI to (a) obtain and (b) retain competitive employment. Augmented SE and SE (including IPS) were the most effective interventions in obtaining competitive employment in comparison to psychiatric care only interventions. SE was also found to be more effective than transitional employment, prevocational training in retaining competitive employment (measured by total weeks of competitive employment worked) at the short-term follow-up (one year or shorter). In the long-term follow-up participants in augmented SE worked the highest number of weeks in competitive employment, followed by those receiving SE.

In addition to the trials included in the systematic review [7], we found 44 further RCTs that tested ways of improving employment rates and job retention, including 7695 participants in total. Of these, 29 studies included participants with SMI (the same population as the above review), seven included participants with CMD, and eight studies reported populations with a variety of mental health conditions. Table 4 shows results of employment interventions in gaining and retaining employment, and education outcomes.

#### Obtaining paid employment

##### CMD populations (***N*** = 5 trials)

Five RCTs were found of interventions intended to improve employment rates in participants with CMD [77–81]. Among these, three utilised models based on IPS [79–81]. On balance this IPS model appeared to be effective in improving rates of competitive employment in these patient populations with only one (low fidelity IPS) study [79] reporting that IPS specifically modified for anxiety and depressive disorders did not improve longer-term employment outcomes compared to treatment as usual. A study of prevocational job skills training intervention [78] involving work situation role play and contact with occupational physicians and work re-integration plans found that long term part-time employment was more likely in the intervention group compared to TAU at both 12 and 24 months. Finally, Beutel et al. [77] reported that after 12 months a transitional employment and psychodynamic treatment intervention showed no significant difference compared to TAU in getting participants into employment, but at 24 months the intervention group were at higher risk of unemployment compared to the TAU group.

##### **SMI populations (*N* = 23 trials)**

Outcomes relating to gaining employment were reported in 23 studies of people with SMI (additional to the above systematic review). Positive outcomes were reported for 10 of these interventions: three of six transitional employment interventions, none of the five prevocational training interventions, one of three SE interventions (a non-IPS badged programme), and six of nine augmented SE interventions.

##### Augmented IPS vs IPS or similar controls

RCTs augmenting SE with cognitive training reported mixed results: three [97–99] interventions increased the likelihood of employment but two [96, 100] studies found no significant difference compared to controls (see Table 2). IPS augmented with cognitive therapy resulted in higher odds of employment than IPS only [113]. Augmentation of SE with job related skills training (e.g. work based problem solving) was associated with both improved employment rates [102, 160] and no significant differences in time to first job [101] (see Table 2).

##### IPS vs other comparators

A large trial by Cook and colleagues [93] reported that SE participants had significantly higher rates of competitive employment at 24 months compared to TAU. Two RCTs [94, 95] reported only short-term benefits [95] or no differences at mid or long term follow up [94].

##### Other employment interventions

Sheltered work was included in six interventions, five of which combined this with cognitive skills training [82–87]. One [82] compared paid sheltered work to unpaid sheltered work, and found that more participants accepted sheltered work if they were paid for their time. The remaining studies of transitional employment which added an element of cognitive training suggested that adding cognitive training to transitional employment may increase the chances of some form of employment when supported and non-competitive employment was also considered [86, 87] at 12 months. However, when only competitive employment was considered, a statistically significant effect tended not to be found for adding cognitive therapy to transitional employment [83–85]. Two studies examined cognitive skills-based prevocational training [89, 90] and one combined prevocational training with cognitive therapy [88]. These did not find significant differences compared to a range of controls in employment rates. Job-focused prevocational training was reported by two studies [91, 92], of which one [92] also added psychiatric elements such as diagnosis and management of difficulties in functioning, comparing this to enhanced vocational rehabilitation. While Gutman et al.’s [91] main focus was on education, one participant in the intervention group accepted employment while none of the control group attained this goal. There were no differences in the number of participants accepting competitive work when a psychiatric job skills training was compared to standard vocational rehabilitation [92].

##### **Mixed populations (*N* = 7 trials)**

Outcomes relating to gaining employment were reported in seven studies in people with mixed or unspecified mental health conditions. Positive outcomes were reported in four of these seven studies- One of three SE studies and three of three augmented SE studies. More participants were in full time employment when a decision aid was used as a means of teaching job skills in a pilot prevocational skills training study [104]. Another study [105] found that low fidelity SE (employment-oriented case management) did not increase rates of employment of any type at the end of the study. High fidelity SE compared to TAU [106] resulted in better odds of being employed at 12 months. When SE was compared to the same intervention with smaller time allowances [107], shorter time budgets gave indication of being better than long term budgets in gaining first employment but results were not significant. Compared to TAU, IPS augmented with cognitive skills training seemed to show a significant benefit in long term employment gain [108, 110] and when employment or education was considered in recording beneficial outcomes [109].

#### Time worked in paid employment

##### **CMD populations (*N* = 5 trials)**

Five RCTs were found of interventions intended to improve length of time continuing work by people with CMD [78–81, 111]. Two of these trials reported positive outcomes. As with employment gain, the two high fidelity IPS trials [80, 81] both found that IPS participants worked significantly more weeks in competitive work than TAU or transitional work controls up to 18 months follow up. However, Hellstrom et al. [79] conducted an adapted IPS intervention specifically for mood and anxiety disorder and did not find that participants worked more hours than a TAU control (matching those findings for gaining employment). Another study [111] compared an augmented SE (with cognitive skills training) with TAU: median months with work were marginally lower in the intervention group (20.3 vs 18.5). A prevocational job skills training intervention [78] reported that compared to TAU, the intervention group recorded significantly more median hours of work at 6 months and 12 months, however by 24 months the median hours worked within the last six months were similar between groups.

##### **SMI populations (*N* = 22 trials)**

Outcomes relating to time spent working in paid employment were reported in 22 studies of people with SMI. Positive outcomes were reported less often for this outcome, with seven studies reporting significant benefits compared to controls: two of the six transitional employment interventions, two of the three prevocational training interventions, and three of 11 augmented SE interventions. One augmented SE intervention reported significant benefits at some timepoints but not others.

##### Augmented IPS vs IPS or similar controls

Several RCTs reported on the effects on time worked from augmenting SE: four trials did not find significant benefits when augmenting SE with job related skills training compared to IPS only [101, 102, 117, 160] and one trial augmenting IPS with cognitive therapy also found no difference in weeks worked compared to IPS only [147]. Augmentation with cognitive skills training showed mixed results across six RCTs [96–100, 118] (see Table 2).

##### IPS vs other comparators

Two studies [94, 95] compared IPS alone to TAU. Erickson, Roes [40] found that IPS did not show a benefit in the number of days worked at 6 or 12 months, but did show a group x time interaction such that the IPS group increased the number of days they were working faster than the TAU group (*P* = 0.03). Killackey et al. [95] also reported that IPS did not improve the number of hours worked compared to TAU.

##### Other employment interventions

Six RCTs compared transitional employment to controls. As with gaining employment, Bell et al., [82] found that participants were more likely to have continued their sheltered employment at 6 months if they were paid. Other RCTs added cognitive skills training [84, 85] or cognitive therapy [112, 113] to transitional employment. Most found that the amount of time spent working was no better than a range of controls [84, 85, 112], though one [113] vocational CBT programme resulted in participants working significantly more weeks at 12 months than TAU controls. Sanches et al. [114] added social skills training to transitional employment but found that participants did not participate in employment for significantly more hours than an active control condition. Three studies reported outcomes relating to time spent working for prevocational training. Behavioural job skills training [115] and training combined with cognitive remediation [89] both resulted in increased time spent working. A Mindfulness-based training pilot study reported a similar number of weeks worked at the end of the 24-month intervention in both groups [116].

##### **Mixed populations (*N* = 4 trials)**

Outcomes relating to time spent in employment were reported in four studies in people with mixed or unspecified mental health conditions. A cognitive based prevocational skills training pilot trial [119] did not show preliminary evidence of added benefit over vocational services alone in the short term, but the remaining three trials comparing augmented SE to TAU controls found that this population responded well to cognitive training as augmentation to SE, working significantly more weeks [108], days [110] and hours [109] than controls.

#### Education

##### **SMI populations (*N* = 5 trials)**

Five RCTs reported education outcomes in SMI populations, though only one intervention was aimed specifically at educational outcomes rather than employment [91]. This skills training-based intervention which taught study skills, time management and basic computer skills among others was the only study to report significant benefits compared to usual care in successful enrolment in education at 6 months. A pattern emerged from two studies using an IPS model [95, 160] such that IPS contributed to short term benefits in getting participants into education, however control TAU groups caught up and ended up with similar numbers studying in longer term (12–24 month) follow ups. Two other prevocational skills training interventions which reported education outcomes, including one that added cognitive therapy [88] and another involving job related skills training [92] did not find improved education outcomes in the medium and long term.

##### **Mixed populations (*N* = 1 trial)**

One study which included participants with both depression and bipolar disorder [108] reported no significant differences in education engagement after SE augmented with cognitive skills training after 12 months.

#### Reducing length of sickness absence in employees

In total, there were nine trials, including 6597 participants meeting criteria for inclusion in the review which focused on reducing the length of sickness absence taken as a result of mental health conditions in employees. All trials were conducted in CMD populations, except for one trial [127] which trained managers to provide mental health support for employees, but did not specify the diagnoses of employees. Table 5 shows results of interventions reporting length of sickness absence.

One study [77] reported that occupational training integrated into psychodynamic treatment had a significantly lower mean duration of sick leave compared to controls. One exposure-based return to work intervention [122] with gradual exposure to progressively more demanding work situations in fact induced a longer time to full return to work compared to TAU (*P* = 0.02) and intervention participants also had a lower likelihood of full return to work. One study trained managers to provide mental health support for employees on sick-leave, however there were no differences in the proportion of sick leave taken between the employees whose managers had received the intervention and employees whose manager had not [127]. The remaining RCTs [120, 121, 123–126] reported no significant benefits in reducing length of sickness absence. These examined a wide range of interventions with varying components such as progressive return to work [121, 123], self-management or psychological training [123–126] and individualised assessment [120, 121, 124].

#### Employment: summary

##### Gaining and retaining employment and education

Findings from this review on interventions for people with severe mental illness to improve employment obtention and retention complement those found by Suijkerbuijk et al. [7] which reported that SE with augmentations (both job related and symptom related) is the best currently available intervention option, alongside IPS only protocols. When considering gaining employment in this review, similar conclusions can be drawn, though it should be noted that while augmented SE is the most widely studied, not all RCTs report that adding treatment elements to IPS contribute significantly above the IPS protocol to employment gain. Evidence for benefits in retention of employment in participants with SMI is more limited and currently interventions show less-clear benefits, with augmented IPS as well as stand-alone IPS showing unclear benefits in improving weeks worked. Limited evidence in mixed diagnosis populations did suggest that cognitive skills training could be a useful addition to IPS however.

In CMD populations, limited evidence suggested that IPS was beneficial in obtaining and retaining employment although alternative strategies were not available for comparison. Only one intervention considered improving education as its primary goal, but this study suggested that training basic skills such as computing and time management may help encourage enrolment. Employment focused interventions do not seem to report educational benefit when this is not specifically targeted.

##### Reducing length of sickness absence

Evidence currently available suggests that interventions so far tested to reduce length of sickness absence are not particularly effective. There remains a lack of research in the area, with the majority of currently available evidence coming from the Netherlands.

### Social isolation and connectedness

In total, there were 20 trials, including 2423 participants meeting criteria for inclusion in the review. Of these, 12 studies included participants with SMI, three included participants with CMD and five were of mixed diagnoses or unspecified. Twelve of these studies reported on subjective social isolation outcomes (including loneliness), one reported on social capital, and 12 reported on objective social isolation outcomes. Social isolation focused interventions were categorised according the classifications in a previous review [31, 38]. Table 2 displays social outcomes for the social isolation domain.

#### Subjective social isolation

##### **CMD populations (*N* = 3 trials)**

Two trials and one feasibility trial aimed to reduce levels of subjective social isolation in people with CMD. Haslam et al.’s [40] psychoeducation programme (“Groups 4 health” social identity intervention) reduced loneliness in the medium term, though one ‘changing cognitions’ intervention, which aimed to use reframing to improve measures of loneliness [39] did not produce significantly different levels of loneliness to controls. A feasibility trial of a supported socialisation intervention, involving support with developing social connections from a “Community Navigator” [41] indicated good acceptability of the approach but did not have sufficient power to detect effects.

##### **SMI populations (*N* = 8 trials)**

Of those interventions aimed at people with SMI, only two of eight trials reported positive results for subjective social isolation. Terzian et al., [45] showed significant benefits in overall quality of intimate and working relationships at one and two years following a supported socialisation social network intervention where staff suggested external social activities of interest for participants. One social cognition and interaction training intervention [42] also reported significant positive medium-term benefits in perceived social support, with intervention participants reporting higher social engagement at the end of the six month intervention. Of the other six trials, interventions involved psychoeducation in one trial [43], supported socialisation in three trials [44–47], and a combination of supported socialisation and psychoeducation in two [48, 49]. None of these found better outcomes for the treatment group compared to controls.

##### **Mixed populations (*N* = 1 trial)**

One supported socialisation intervention included participants with both CMD and SMI [50], but this peer assisted case management intervention did not demonstrate significant differences in subjective quality of social relations at medium or long-term follow up.

#### Social capital

##### **CMD populations (*N* = 1 trial)**

One feasibility RCT [41] reported results for social capital in participants with CMD, finding similar scores for social capital in the intervention and the control group at the end of the six months supported socialisation intervention.

#### Objective social isolation

##### **SMI populations (*N* = 10 trials)**

Positive results were reported for seven of the trials targeting objective social isolation among people with SMI. Interventions in four studies were based on changing cognitions [42, 51–53], two on supported socialisation [44, 56], two on social skills training [54, 55] and two on a combination of approaches [49, 57]. Only one of four changing cognitions interventions [53], a social cognition and interaction training intervention, showed positive medium-term benefits compared to TAU controls, while the remaining three, another social cognition and interaction training programme [42], a social activation-focused Cognitive behavioural therapy (CBT) programme [51] and a virtual reality- based CBT programme involving social situation exposure [52], did not show benefits compared to either TAU or social mentoring active controls. Social skills training interventions [54, 55] showed increases in social contacts (outside of intervention contacts) at short and mid-term follow ups, and both found that social functioning measures improved at long-term follow up compared to active controls, with Marder et al. [55] suggesting that the biggest advantages may stem from combining social skills training with drug treatment. Both supported socialisation interventions, one which used humour as a bonding facilitator with peers [44] and the other which facilitated befriending with a volunteer [56] reported increased social contacts in the short to medium term. Mixed approach interventions also showed a benefit in increasing social contacts for participants with SMI; these included a mix of supported socialisation and psychoeducation in a guided peer support intervention [49], which improved the number of social contacts with peers after the eight month intervention and a mix of changing cognitions and social skills training in a CBT social skills intervention [57] which also reported improved numbers of social activities reported on the social adjustment scale compared to treatment as usual at six months.

##### **Mixed populations (*N* = 1 trial)**

Another supported socialisation study [50] which involved helping participants to engage in social activities to develop social networks included participants with both CMD and SMI. This study did not show significant improvements in objective social isolation (number of contacts) at six and 12 months.

#### Social isolation- summary

At present, we have very little trial evidence about how to address loneliness/subjective social isolation for populations with mental health conditions, with mixed results and no clear pattern in intervention strategies producing benefits. Objective social isolation appeared to improve in participants with SMI with a range of approaches such as supported socialisation and social skills training, particularly in the medium term. Limited focus specifically on social capital prevents conclusions on how best to target this.

### Family, intimate and caring relationships

We did not find any systematic reviews or RCTs directly addressing the achievement or sustainment of intimate partner or family member roles, or maintenance of informal caring roles or custody of children.

### Victimisation and exploitation

We also found only one RCT aiming to reduce victimisation in people with SMI [140]. This study from the Netherlands included 250 participants and used a manualized group training programme focused on enhancing emotion regulation, conflict resolution and street skills (SOS training) and found that care as usual plus SOS training was more effective in preventing victimisation than care as usual alone but the results were inconclusive: significantly more participants in the experimental group (67.6%) achieved a treatment response for total victimization compared to the control group (54%) at 14 months post baseline, and this difference was significant (OR = 1.78, 95% CI: 1.02–3.11, *P* = 0.042). However, when the focus was narrowed to include only violent victimisation instead of all victimisation, the difference did not reach statistical significance (OR = 1.75, 95% CI: 0.91–3.34, *P* = 0.092).

### Offending

In total, eight RCTs, including 1148 participants met criteria for inclusion in the review which focused on offending. Of these, seven studies focused on participants with SMI, of which three included participants with a dual diagnosis of SMI and substance use disorder. One study included participants with a mixture of mental health conditions. All eight studies reported on outcomes relating to offending or reoffending. Table 6 shows the results of each study and key components of interventions.

#### Offending or reoffending

##### **SMI populations (*N* = 7 trials)**

For people with SMI, court ordered treatment was part of the intervention in two studies [128, 131]. While Cosden et al. [128] did not find that outcomes were improved in mental health treatment court participants vs TAU, Lamberti et al., [131] found that court-ordered treatment combined with forensic Assertive Community Treatment did have a small effect in reducing convictions. Two other studies were of interventions which included multi-disciplinary team support [129, 130]. These programmes also both included a specified drug and alcohol programme aspect, but neither found significant differences compared to usual services. In total, drug and alcohol programmes were part of four interventions [128–130, 132], but these trials failed to report benefits at long term follow up. Of two interventions which included a specified (predominantly psychoeducation and cognitive behavioural based) psychological therapy [133, 134], only one [134] found that their intervention group had fewer reincarcerations than the standard care group, however, this prison modified therapeutic community utilised a similar protocol to Sacks et al. [133], where no benefits were found.

##### **Mixed populations (*N* = 1 trial)**

One study included populations with a mixture of mental health conditions [135], finding that a group psychotherapy programme focusing on self-control, emotion management and problem solving did not reduce offending compared to TAU at 18 months.

#### Offending: summary

Evidence is limited for interventions which aim specifically to reduce offending or reoffending in populations with mental health conditions. Only two of eight studies (Forensic ACT; [131] and Prison modified therapeutic community [134]) reported significant benefits compared to controls. Given these involved different approaches to intervention: we cannot be certain about the most effective approaches or essential intervention components.

### Rights, inclusion and citizenship

Only two RCTs tested interventions intended to improve rights, inclusion or citizenship outcomes in people with SMI [137] or a mixture of diagnoses [136], including a total of 605 participants. We did not find any RCTs addressing lack of privacy or dignity. Table 7 shows the results of interventions reporting Rights outcomes.

#### Participation

##### **SMI populations (*N* = 1 trial)**

Segal et al. [137] found that adding self-help agencies to community mental health agencies contributed to improvements in independent social integration which includedsocial presence (the feeling of being there with a real person), access, participation, production, and employment.

##### **Mixed populations (*N* = 1 trial)**

Salzer et al. [136] found that peer-delivered core services of centres for independent living did not significantly improve the number of social participation days reported, compared to TAU over time at six and 12 months.

#### Access to services

##### **Mixed populations (*N* = 1 trial)**

Salzer et al. [136] also reported that while the number of reported unmet needs of participants decreased, these did not differ significantly from the TAU group.

#### Rights, inclusion and citizenship: summary

Currently available evidence for the impact of interventions to improve participation in communities and access to services is very limited, and there remain significant gaps in the literature regarding improving privacy and dignity. It is unclear whether self-help agencies and peer support could have positive impacts on these outcomes.

### Secondary outcomes

#### Mental health

A total of 54 studies reported mental health symptom severity outcomes alongside social outcomes (Social isolation *N* = 13, housing *N* = 15, offending *N* = 2, employment *N* = 21, rights inclusion and citizenship *N* = 2, and victimisation *N* = 1). Only 14 of these 54 studies reported benefits for the intervention group compared to the control group: (social isolation: *N* = 2 CMD, *N* = 1 SMI; employment: *N* = 2 CMD, *N* = 1 SMI, *N* = 1 mixed; housing: *N* = 3 SMI; offending *N* = 2 SMI; rights, inclusion and citizenship: *N* = 2). However, no study reported that mental health symptoms were significantly worse than the control group, with the other 40 studies reporting no differences between arms.. Similarly, of eight RCTs reporting mental health service use (employment *N* = 1, housing *N* = 4, offending *N* = 2, social isolation *N* = 1), four reported no differences in hospitalisations between groups, though three housing interventions [59, 69, 70] reported that their intervention groups (ACT, residential treatment, and Housing First, respectively) resulted in fewer days in psychiatric hospitals compared to treatment as usual. Further detail on mental health symptom outcomes is available in Additional File 6.

#### Quality of life

Nineteen RCTs reported quality of life outcomes (Seven social isolation, four housing, one offending, seven employment, two rights, inclusion and citizenship), while three reported life satisfaction (Two housing, one social isolation) and one reported wellbeing (Social isolation). Six of these 19 trials reported positive quality of life outcomes for the treatment group compared to control (usually TAU) groups. These positive trial results were found across three life domains (Employment SMI *N* = 1, mixed *N* = 1; Housing SMI *N* = 3; offending SMI *N* = 1). We cannot discern evident patterns identifying those clinical groups or intervention types where quality of life improvements was most likely to be achieved. Further details on quality of life outcomes are available in Additional File 7.

#### Costs and cost-effectiveness

Sixteen studies were identified with sufficient information to enable them to be classified as economic evaluations (see Additional File 8 for further details). This included cost comparisons (*N* = 6), studies combining costs and outcomes either directly in the form of a ratio (*N* = 6) or return on investment (*N* = 2) or indirectly where cost and outcomes are reported alongside each other (*N* = 2). Overall, the economic evidence is reasonably strong in favour of social interventions, particularly when these focussed on housing and employment. Only a small number of studies measured outcomes using quality adjusted life years (QALYs). Use of QALYs can help decision makers to compare across different areas of health, but they focus on functioning and health status rather the achievement of specific social outcomes. As such it was not unexpected to see them rarely used in the evaluations reviewed here, and indeed their use may not have been appropriate.

## Discussion

### Summary of findings

The results of this evidence synthesis highlight a number of important findings. Adding to the evidence gathered through a Cochrane review [7], we found a growing literature base which gave some indication that IPS could be beneficial in improving employment rates for people with SMI, and that augmentation through adding additional intervention components may be beneficial in some circumstances. There was also some evidence that this could be extended to support people with CMDs or to encourage people to enrol in educational courses. Similarly, there is a strong evidence base for Housing First interventions, which provided international evidence from large scale trials that people with SMI who are homeless can benefit from programmes that prioritise providing stable housing in the first place, with clinical and social support linked to this subsequently [58–60]. Finally, we found some evidence that objective measures of social isolation can be improved through interventions focused on supporting socialisation or training socialisation skills.

However, the overall picture from our review is of very large gaps in the evidence. Several social domains almost entirely lack an evidence base even though they are not only outcomes that are highly valued by service users and carers, but are also implicated as risk factors for onset and continuation of mental health conditions. For example, debt can increase risk of mental health disorders six-fold [161], yet despite its clear importance as a determinant of mental health outcomes, we found only one RCT with any monetary focus. Other notable omissions in the data include interventions to improve successful community living after offending, engagement in meaningful activity (outside of employment), lack of privacy, exploitation, family relationship roles, rights and participation, and victimisation. The lack of RCT trials of interventions to improve retention of caring family roles including parenthood, or to help people establish satisfying intimate relationships is an important gap given the importance of these areas in people’s lives and the proven link between family roles and social isolation [15]. Similarly, prevention of victimisation was addressed in only one RCT. It is of particular interest that interventions to prevent offending were more commonly reported than those to prevent people with mental health conditions from being victims of crime, as these are often outcomes which are highly correlated [162], and therefore could well benefit from interventions with a dual focus.

In other areas, evidence remains weak, with most trials to date remaining preliminary. For example, clear evidence on the best ways to address loneliness in people with mental health conditions remains elusive, in spite of repeated calls for this to be a priority [16], and little progress has been made in developing interventions which reduce offending in people with mental health conditions, despite strong associations between them [163]. Success of interventions such as IPS and Housing First could shed some light onto how best to facilitate improvements in domains such as this.For example the “place then train” approach may be one that can be adapted to improve social functioning. Furthermore, despite a considerable pool of evidence, it remains unclear how best to augment IPS to further improve employment rates and retention. For example, though both IPS and augmented IPS show clear benefits compared to other interventions (e.g. [97, 99, 147]), when directly compared, it was not clear that augmentation had an additive impact. Finally, while Housing First programmes have demonstrated positive outcomes relating to gaining housing in those who are without stable housing at baseline (e.g. [58, 60]), it remains unclear how best to support those who are not homeless to retain the stability in their tenancies, and little focus has been on improvement in perceived housing quality, something which has been associated with an exacerbation of clinical symptoms [164].

### Implications for research

The current review highlights important gaps in the literature regarding the effectiveness of social interventions, despite the emphasis placed on improvement in these domains by service users [3]. Provision of more high-quality trial data may help to identify the best way to integrate social interventions into current practice. The success of some interventions provides three potentially generalisable indications of what may be required to improve social circumstances across life domains. First, similarly to IPS and Housing First interventions, interventions which directly target the desired social circumstance, rather than providing an interim staged approach may result in greater benefit. Second, successful interventions identified in this review suggest that high-intensity support may be required to achieve improvements in social circumstances. Third, although we did not find a clear pattern of provision of multi-disciplinary team support, there is an indication that the enhanced and comprehensive care integration typical of both Housing First and IPS are important in producing positive outcomes [165, 166], and this may be an influential factor in interventions which improve other social circumstances. For example, a more detailed focus on debt restructuring and support with utilities companies and landlords may be of more benefit than a narrower focus on financial education [141].

Our synthesis of secondary outcomes suggests that socially focused programmes can improve symptom severity and quality of life, but do not necessarily do so. It is likely that security across multiple social domains, and multiple aspects within each domain, alongside effective treatment, could play a major role in facilitating improved outcomes [4]. Future trials are likely to be more successful if they have a clear theoretical basis alongside co-production to ensure that they reflect service user and carer priorities.

### Implications for practice

This review also highlights important implications for future practice. Firstly, despite guidance (e.g. NICE) suggesting that SE should be integrated into care for people with severe mental health conditions [167], this is not routinely provided in all service settings or to all service users who want to find work in the UK [168] or internationally [169]. Given the often-cited goal of patients with SMI of returning to employment [170], widespread implementation of IPS services should be considered a key policy focus. Our findings suggest IPS employment support may also be helpful for people with other mental health conditions, and be able to help address low rates of employment among people with all mental health conditions [171]. Secondly, Housing First trials have demonstrated that participants remain in stable housing for longer compared to controls, indicating that this is a key intervention which could be implemented to reduce the number of people with mental health conditions who are rough sleeping or whose difficulties are exacerbated by insecure housing. However, implementation of this complex intervention may rely heavily on additional context-specific supporting evidence to encourage more long-term funding for services in national policy and service planning [172, 173].

The World Health Organization has highlighted the need for integrated support for people with mental health conditions, such that their clinical and social circumstances are jointly targeted within care [174]. This notion, though most commonly seen within housing interventions [175, 176] could be extended further to improve other social circumstances. Combining interventions to focus on, for example, both placing patients in employment as well as training cognitive coping strategies in augmented IPS gave some indications of being beneficial, and was also supported in conclusions drawn in a Cochrane review [7]. Integration of approaches to different social domains may also be useful to avoid fragmentation of care and ensure a holistic and comprehensive approach to support. Social interventions remain a comparatively untested approach to trying to improve outcomes and help people with mental health conditions live lives that they value, extending beyond a narrow focus on clinical symptom severity to a broader person-centred approach to recovery, and should thus be a clear focus of policy and practice [1].

### Limitations

Despite the implications raised within this review, a number of limitations should be noted. First, our aims, which were to conduct a very broad stocktake of the current state of the evidence across multiple social domains resulted in extremely heterogenous data which could not be quantitatively pooled. However, consideration of potentially useful avenues for future research through a narrative synthesis allows for reflection on more complex and diverse data [177], making it a beneficial strategy in the context of this review. For most domains, we were unable to identify established intervention typologies with which to categorise the programmes reported in this review. Our focus on randomised controlled trials may have meant that key literature examining social domains less well covered within a randomised method were missed, for example many efforts to get employees back to work following sickness absence are made, but do not necessarily get compared in trials [178]. Furthermore, our focus on individual level interventions means that organisational and population level interventions could also have been missed. Because of this, a full examination of additional literature which may help to shed light on what sorts of interventions may work for specific populations, and most promising approaches not evaluated in trials, may be an important focus for future research. Our review also only included interventions which directly focused on our selected social outcomes. We have therefore excluded pharmacological or psychological interventions which may help to improve social outcomes by reducing illness severity or changing thinking and behaviour, if their primary aim was not to improve our selected social outcomes. For example, we have not included trials of family interventions which seek to improve health outcomes by helping family communications and problem solving, where these programmes did not explicitly focus on helping people to maintain family or caring roles, which were our included outcomes. Lastly, the focus of our review on interventions which have been evaluated specifically for people with mental health conditions meant that we did not consider interventions to improve social circumstances which have established evidence of effectiveness in the general population, but which may also be helpful for people with mental health conditions.

## Conclusion

In conclusion, there is a large body of literature examining how best to support people with mental health conditions in some aspects of their lives, such as employment, housing, and objective social isolation, and particularly in well-studied interventions such as IPS and Housing First can help to improve people’s social circumstances. Other research has indicated that it is possible to support people to improve other aspects of their social circumstances, but more high quality evidence is required in a number of areas which contribute to significant risk for people with mental health conditions- additional research focus and resource for targeting social domains such as money and debt, rights, inclusion and citizenship, victimisation (and its links with offending), and family and caring relationships could contribute significantly to positive changes for people with mental health conditions. More broadly, integration of social support within health and social care services could be an important focus for policy and practice.

## Lived experience commentary

This comprehensive paper attempts to cover all important domains in pulling together 20 years’ evidence. However, despite its broad coverage, from a Lived Experience perspective, it lays bare the large gaps in data and absence of granular detail. It highlights the fundamental lack of evidence for interventions to support people’s social needs including basic needs, citizenship and rights, in the context of the whole family or community.

This research study isolates each domain, but in real life, domains interact with each other or occur in varying sequences. Each study assumes a homogeneity of the people involved without addressing specific groups. With little detail of what might work for whom or when, this spotlights a significant gap in knowledge.

People who have lived experience of these issues welcome a focus on social needs, but may raise alternative research questions. With first-hand experience of the impact of immigration or homelessness, we place an emphasis on prevention across our needs rather than interventions which are too late to address any one of our challenges. We also value services which are offered sensitively and effectively to meet the varying needs of a range of people whose first language may not be English or who may have survived specific traumatic experiences. Involving lived experience researchers is essential to ensuring the research questions are relevant to real life in all its variety, and to maintain a focus on the acceptability of any interventions from the perspective of service users and their carers.

Funding is fundamental to all social needs and associated interventions, whether that is the personal lack of money to attend appointments, the debt that led to housing problems, or the historically insufficient resources in the system to provide mental health support. Issues around money, including benefits, poverty, financial difficulties, access to services, the impact on the built environment, and digital exclusion, need to have greater emphasis in future studies.

No research since March 2020 can occur without mentioning COVID-19 which has rapidly impacted people’s lives, and increased social, economic, and health inequalities. This shift demands that people’s social needs receive much greater attention: earlier, quicker, and more adapted to individual lives and their complexity.

## Supplementary Information

**Additional file 1. **Search Strategy. The search strategy used to source texts.

**Additional file 2. **Outcome Hierarchy. The tool used to select which outcomes to extract when multiple items for each outcomes were reported.

**Additional file 3.** TIDIER Checklist. Additional information regarding the interventions in each study reported according to the TIDIER checklist.

**Additional file 4. **ROB description across domains. Further description of variations in study quality across domains.

**Additional file 5. **Housing First Trial additional papers. A list of additional publications relating to the Housing First Trial conducted in Canada, not extracted in this review due to irrelevant outcomes and overlapping data.

**Additional file 6.** Mental health outcomes. Additional information on mental health outcomes of the interventions.

**Additional file 7. **Quality of life outcomes. Additional information on quality of life outcomes of the interventions.

**Additional file 8. **Costs. Additional information on cost analyses reported within studies.

## Data Availability

The datasets used and/or analysed during the current study are available from the corresponding author on reasonable request.
