# Supplementary material for: Interventions to improve social circumstances of people with mental health conditions: a rapid evidence synthesis
Source: BMC Psychiatry. 2022 Apr 28;22:302. doi: 10.1186/s12888-022-03864-9 (PMC9047264; doi:10.1186/s12888-022-03864-9)
Supplement: Supplementary file 1 — Additional file 1. Search Strategy. The search strategy used to source texts. [file 12888_2022_3864_MOESM1_ESM.docx]

# Systematic review stage: example strategy (MEDLINE)

Database: Ovid MEDLINE(R) and Epub Ahead of Print, In-Process & Other Non-Indexed Citations and Daily <1946 to January 23, 2020>

Search Strategy:

--------------------------------------------------------------------------------

1 mental disorders/ or anxiety disorders/ or agoraphobia/ or anxiety, separation/ or neurocirculatory asthenia/ or neurotic disorders/ or obsessive-compulsive disorder/ or hoarding disorder/ or panic disorder/ or phobic disorders/ or phobia, social/ or "bipolar and related disorders"/ or bipolar disorder/ or trichotillomania/ or dissociative disorders/ or multiple personality disorder/ or "feeding and eating disorders"/ or anorexia nervosa/ or binge-eating disorder/ or bulimia nervosa/ or mood disorders/ or depressive disorder/ or depression, postpartum/ or depressive disorder, major/ or depressive disorder, treatment-resistant/ or dysthymic disorder/ or premenstrual dysphoric disorder/ or seasonal affective disorder/ or cyclothymic disorder/ or mutism/ or reactive attachment disorder/ or "fetishism (psychiatric)"/ or personality disorders/ or antisocial personality disorder/ or borderline personality disorder/ or compulsive personality disorder/ or dependent personality disorder/ or histrionic personality disorder/ or hysteria/ or paranoid personality disorder/ or passive-aggressive personality disorder/ or schizoid personality disorder/ or schizotypal personality disorder/ or "schizophrenia spectrum and other psychotic disorders"/ or affective disorders, psychotic/ or capgras syndrome/ or delusional parasitosis/ or paranoid disorders/ or psychotic disorders/ or psychoses, substance-induced/ or psychoses, alcoholic/ or schizophrenia/ or schizophrenia, catatonic/ or schizophrenia, disorganized/ or schizophrenia, paranoid/ or shared paranoid disorder/ or sexual dysfunctions, psychological/ or somatoform disorders/ or body dysmorphic disorders/ or conversion disorder/ or hypochondriasis/ or munchausen syndrome/ or munchausen syndrome by proxy/ or neurasthenia/ or "trauma and stressor related disorders"/ or adjustment disorders/ or stress disorders, traumatic/ or combat disorders/ or psychological trauma/ or stress disorders, post-traumatic/ or stress disorders, traumatic, acute/ (566973)

2 catatonia/ or delusions/ or depersonalization/ or depression/ or malingering/ or obsessive behavior/ or stalking/ or paranoid behavior/ or schizophrenic language/ or self-injurious behavior/ or self mutilation/ or suicide/ or suicidal ideation/ or suicide, attempted/ or impulsive behavior/ or compulsive behavior/ or behavior, addictive/ or hoarding/ (205479)

3 Mental Health/ or (mental* adj2 (health* or ill*)).ti,ab,kf. (182830)

4 mental health services/ or community mental health services/ or community psychiatry/ (51809)

5 (acute stress or adjustment disorder* or ADNOS or affective disorder* or agoraphobi* or anorexia nervosa or anxiety or astheni* or attachment disorder* or BPD or binge eat* or binging or bipolar or body dysmorphi* or bulimi* or catatoni* or combat disorder* or compulsi* or conversion disorder* or cyclothymi* or delusion* or depersonali#ation or depressed or depression or depressive or dissociative disorder* or dyssomni* or dyspareunia* or dysphori* or dysthymi* or dystoni* or eating disorder* or EDNOS or emotional trauma or fear or health anxiety or hoarding or hyperactivity or hypochondri* or hysteri* or medically unexplained or malingering or mania or manic or MDD or mental* or mood? or munchausen or MUPS or mutism or neurastheni* or neurotic or neuros* or obsess* or panic or paranoi* or parasuicid* or perceptual disorder* or personality disorder* or phobi* or PND or ((post-trauma* or posttrauma*) adj stress*) or psychiatr* or psychogenic or psychopathol* or psychosomatic or psychotic or psychos* or PTSD or schizo* or (self adj (injur* or harm or mutilat*)) or (sexual dysfunction* adj3 psycho*) or social anxiety or somati* or somatoform or suicid* or trichotillomani* or stalking).ti,kf,kw. (864045)

6 or/1-5 (1158443)

7 ((chang* or develop* or enhanc* or initiative? or intervention? or program* or mitigat* or address* or improv* or target*) adj3 (community or living or social) adj3 (condition? or circumstance?)).ti,ab,kf. (1726)

8 social prescribing.ti,ab,kf. (113)

9 ((communit* or social) adj (connect* or engagement? or link* or referral? or intervention? or wellbeing)).ti,ab,kf. (9740)

10 "sense of belonging".ti,ab,kf. (886)

11 or/7-10 (12384)

12 6 and 11 (2481)

13 HOUSING/ (17140)

14 homeless persons/ or homeless youth/ (8613)

15 ((chang* or develop* or enhanc* or initiative? or intervention? or program* or mitigat* or address* or improv* or target*) adj3 (housing or neighbo?rhood?)).ti,ab,kf. (4438)

16 homeless*.ti,ab,kf. (10246)

17 ((housing adj (first or stability or instability)) or permanent housing).ti,ab,kf. (776)

18 housing.ti. or ((housing adj (strateg* or polic* or project* or program* or quality)) or new* buil* or social housing*).ti,ab,kf. (8502)

19 *independent living/ or *assisted living facilities/ or *group homes/ or *halfway houses/ or *public housing/ or residence characteristics/ (37492)

20 poverty areas/ (5938)

21 ((autonomous or assisted or sheltered or support*) adj3 (housing or accomodation or dwelling?)).ti,ab,kf. (1421*) [N.B. Typo corrected in a later Ovid cross-search, see below]*

22 (((clubhouse or club house) adj model?) or ((autonomous or independent or assisted) adj living)).ti,ab,kf. (4815)

23 ((independ* or assist* or support* or secur* or sustain* or maint*) adj3 (tenanc* or tenure?)).ti,ab,kf. (139)

24 ((halfway or satellite) adj (dwelling? or home? or house?)).ti,ab,kf. (232)

25 (neighbo?rhood? adj (characteristic* or intervention* or program*)).ti,ab,kf. (1461)

26 ((environment* or housing or neighbo?rhood?) and infrastructure).ti,ab,kf. (5442)

27 built environment/ (333)

28 or/13-27 (85505)

29 6 and 28 (12717)

30 MONEY.ti,kf. (5314)

31 economic status/ or exp income/ or exp pensions/ or remuneration/ or exp "salaries and fringe benefits"/(62214)

32 Financing, Personal/ (5483)

33 ((access* or improv* or manag* or supplement*) adj2 (cash or money or financ* or income? or savings)).ti,ab,kf. (6537)

34 ((financial adj (autonomy or security or insecurity)) or loans or borrowing or budgeting or microcredit or microfinance or social fund*).ti,ab,kf. (5277)

35 Poverty/pc [Prevention & Control] (561)

36 high poverty.ti,ab,kf. or poverty.ti. (4864)

37 ((address* or escap* or improv* or support* or target*) adj2 (depriv* or poor or poverty)).ti,ab,kf. (5307)

38 "out of poverty".ab. (125)

39 (((food or fuel) adj poverty) or food bank?).ti,ab,kf. (337)

40 ((alleviat* or ease or manag* or prevent* or reduc* or stop*) adj2 (poverty or ((economic or financial) adj hardship?))).ti,ab,kf. (1943)

41 ((alleviat* or ease or manag* or prevent* or reduc* or stop*) adj debt?).ti,ab,kf. (26)

42 debt?.ti,kf. (1570)

43 (introduction adj3 (basic or minimum) adj3 (wage? or income?)).ti,ab,kf. (0)

44 paid work.ti,ab,kf. (1045)

45 "dealing with money".ab. (7)

46 (family adj (income? or tax credit?)).ti,ab,kf. (6388)

47 *Social Welfare/ (5463)

48 welfare benefit?.ti,ab,kf. (480)

49 or/30-48 (105521)

50 6 and 49 (8684)

51 EMPLOYMENT/ (45196)

52 employment, supported/ (1166)

53 rehabilitation, vocational/ (9367)

54 unemployment/ (6716)

55 ((chang* or develop* or enhanc* or initiative? or intervention? or program* or address* or improv* or target*) adj3 (employment or unemployment or unemploy*)).ti,ab,kf. (3417)

56 (support* adj3 (employment? or work or vocational)).ti,ab,kf. (11552)

57 (employment or unemployment).ti. (11069)

58 individual placement?.ti,ab,kf. (322)

59 ((finding or gaining or obtaining or keeping or sustaining) adj3 (work or job or employment)).ti,ab,kf. (1914)

60 (social firms or (sheltered adj (employment or work))).ti,ab,kf. (136)

61 (precar* adj1 (employment or work)).ti,ab,kf. (226)

62 paid work.ti,ab,kf. (1045)

63 (voluntary work or volunteering).ti,ab,kf. (2010)

64 (meaningful adj (activit* or employment or work)).ti,ab,kf. (912)

65 ("return to work" or "back to work" or absenteeism).mp. (21774)

66 ((alleviat* or ease or manag* or prevent* or reduc* or stop*) adj work* disabilit*).ti,ab,kf. (73)

67 "return* to education".ab. (82)

68 ((education or learning or training) adj3 (access* or takeup or take up)).ti,ab,kf. (4401)

69 ((labo?r force or employment or unemployment) adj status).ti,ab,kf. (200)

70 or/51-69 (101045)

71 6 and 70 (15781)

72 SOCIAL ISOLATION/ (12965)

73 *loneliness/ or social alienation/ (3392)

74 (loneliness or social inclusion or social participation).ti,kf. or (communit* and (social adj (isolation or participation))).mp. (6502)

75 ((subjective or objective) adj social isolation).ti,ab,kf. (38)

76 ((chang* or develop* or enhanc* or initiative? or intervention? or program* or mitigat* or address* or improv* or target*) adj5 (loneliness or ((social or community) adj (connect* or inclusion or isolation or network? or participation or relations*)))).ti,ab,kf. (5418)

77 ((alleviat* or ease or manag* or mitigat* or prevent* or overcom* or reduc* or stop*) adj5 (social* or communit*) adj5 (alienat* or discriminat* or excluded or exclusion or isolated or isolation)).ti,ab,kf. (895)

78 ((alleviat* or ease or manag* or mitigat* or prevent* or overcom* or reduc* or stop*) adj2 (isolation or isolated or exclusion or excluded)).ti,ab,kw. (4506)

79 ((address* or enhanc* or improv* or increas* or promot* or target*) adj2 (inclusion or inclusivity)).ti,ab,kf. (2465)

80 ((address* or enhanc* or improv* or increas* or promot* or target*) adj3 (social* or communit*) adj3 network?).ti,ab,kf. (693)

81 social participation/ (2202)

82 ((social or psychosocial) adj support*).ti,kf. (11132)

83 (social adj (capital or mobilit*)).mp. (5057)

84 navigator?.ti,ab,kf. or (peer? adj1 support*).ti,kf. (4500)

85 (anti-stigma* or ((intervention? or alleviat* or prevent* or reduc* or stop*) adj2 stigma*)).ti,ab,kf. (3239)

86 or/72-85 (54122)

87 6 and 86 (15602)

88 FAMILY RELATIONS/ (10845)

89 family conflict/ or intergenerational relations/ (5716)

90 ((family or families or intergenerat* or inter-generat*) adj (relation* or conflict?)).ti,ab,kf. (9362)

91 ((sexual or intimate or partner?) adj (relation* or conflict?)).ti,ab,kf. (9709)

92 ((develop* or enhanc* or initiative? or intervention? or program* or address* or improv* or promot* or target*) adj2 relationship?).ti,ab,kf. (13524)

93 ((carer? or partner or relationship?) adj support*).ti,ab,kf. (1280)

94 (child* adj2 (access or contact or custody or maintenance)).ti,ab,kf. (3354)

95 ((care proceeding? or family court? or child removal or fostercare or foster care) and (parent* or mother? or father?)).mp. (1024)

96 parenting/ (16107)

97 parent* outcome?.ti,ab,kf. (372)

98 or/88-97 (65179)

99 6 and 98 (14567)

100 parental mental health.ti,ab,kf. (394)

101 (99 or 100) (14854)

102 (VICTIMIZATION or victimisation or revictimi#ation).mp. (8820)

103 crime victims/ or "adult survivors of child abuse"/ (11043)

104 (crime victim? or revictimi*).ti,ab,kf. (673)

105 ((victim* or crime?) and survivor*).ti,ab,kf,hw. (3179)

106 Domestic Violence/ or Battered Women/ or Spouse Abuse/ (13676)

107 ((domestic or partner? or spouse?) adj3 (abus* or violen*)).ti,ab,kf. (14828)

108 ((domestic or marital or partner? or spous*) adj3 (rape or sex* assault*)).mp. (329)

109 (intimate partner adj2 abus*).ti,ab,kf. (379)

110 coercive control.ti,ab,kf. (138)

111 ((female? or women?) adj (refuge? or shelter?)).ti,ab,kf. (160)

112 (exploitation or safe guarding or safeguarding).ti,ab,kf. (20770)

113 or/102-112 (55734)

114 (6 and 113) (12442)

115 OFFENDING.ti,kf. (899)

116 Recidivism.ti,kf,sh. (1446)

117 ((crime? or criminal* or offend* or offence? or recidiv*) adj3 (initiative? or intervention? or program* or mitigat* or address* or rehabilitat*)).ti,ab,kf. (1330)

118 ((crime? or criminal* or offend* or offence? or recidiv*) adj3 (diver* or prevent*)).ti,ab,kf. (1045)

119 prisoners/ (16232)

120 ((inmate? or prisoner? or convict? or felon?) adj3 (rehabilitat* or releas*)).ti,ab,kf. (455)

121 (community adj2 (reentry or re-entry)).ti,ab,kf. (202)

122 or/115-121 (20082)

123 6 and 122 (7054)

124 HUMAN RIGHTS/ or CIVIL RIGHTS/ or SOCIAL JUSTICE/ (33968)

125 (((citizen? or civil* or human or legal or social or voting) adj rights) or social justice or equal protection or social protection).ti,ab,kf. (15440)

126 rights.ti,hw. (44081)

127 *public policy/ or social polic*.ti,ab,kf. (20382)

128 equity-focus*.ti,ab,kf. (116)

129 "Social Determinants of Health"/ (2636)

130 ((social or community or neighbo?rhood?) adj3 (equit* or inequit* or inequalit*)).ti,ab,kf. (6028)

131 (digital adj (inclusion or exclusion or divide or equit* or inequit* or inequalit*)).ti,ab,kf. (528)

132 or/124-131 (87087)

133 (6 and 132) (8238)

134 (systematic or structured or evidence or trials or studies).ti. and ((review or overview or look or examination or update* or summary).ti. or review.pt.) (199027)

135 (0266-4623 or 1469-493X or 1366-5278 or 1530-440X or 2046-4053).is. (18594)

136 meta-analysis.pt. or (meta-analys* or meta analys* or metaanalys* or meta synth* or meta-synth* or metasynth*).ti,ab,kf,hw. (193412)

137 ((systematic or meta) adj2 (analys* or review)).ti,kf. or ((systematic* or quantitativ* or methodologic*) adj2 (review* or overview*)).ti,ab,kf,sh. or (quantitativ$ adj2 synthesis$).ti,ab,kf,hw. (248479)

138 (integrative research review* or research integration).tw. or scoping review?.ti,kf. or (review.ti,kf,pt. and (trials as topic or studies as topic).hw.) or (evidence adj3 review*).ti,ab,kf. (189612)

139 review.pt. and (medline or medlars or embase or pubmed or scisearch or psychinfo or psycinfo or psychlit or psyclit or cinahl or electronic database* or bibliographic database* or computeri#ed database* or online database* or pooling or pooled or mantel haenszel or peto or dersimonian or der simonian or fixed effect or ((hand adj2 search*) or (manual* adj2 search*))).tw,hw. (149262)

140 or/134-139 (534039)

141 ((child* or adolescen*) not (childbirth or adult* or family or families or mother? or woman* or women* or female? or father? or men or mens or male? or relations or former)).ti. (767066)

142 prevalence.ti. (128833)

143 140 not (141 or 142) (512063)

144 12 and 143 (141) [Linking interventions]

145 29 and 143 (339) [Housing]

146 (50 and 143) (223)

147 (71 and 143) (545)

148 (146 or 147) (691) [Money or Employment]

149 (87 and 143) (530) [Social Isolation]

150 (101 and 143) (474) [Family Relations]

151 (114 and 143) (343)

152 (123 and 143) (158)

153 (151 or 152) (498) [Victimization or Offending]

154 (133 and 143) (189) [Rights]

155 or/144-154 (2539)

Ovid XSearch - Accommodation (typo correction): PsycINFO <1806 to February Week 1 2020>, Journals@OVID, PsycARTICLES Full Text, Embase <1974 to 2020 Week 06>, Ovid MEDLINE(R) and Epub Ahead of Print, In-Process & Other Non-Indexed Citations and Daily <1946 to February 07, 2020>

Search Strategy:

--------------------------------------------------------------------------------

1 systematic review/ or meta analysis/ or network meta-analysis/ (513932)

2 ((systematic or structured or evidence or trials or studies) and (review or overview or look or examination or update* or summary)).ti. (400036)

3 (0266-4623 or 1469-493X or 1366-5278 or 1530-440X or 2046-4053).is. (45607)

4 (systematic review? or evidence report* or technology assessment?).jw,jx. (54310)

5 (meta-analys* or meta analys* or metaanalys* or meta synth* or meta-synth* or metasynth*).ti,ab,id,kf,kw,hw. (583547)

6 ((systematic or meta) adj2 (analys* or review)).ti,id,kf,kw. or ((systematic* or quantitativ* or methodologic*) adj5 (review* or overview*)).ti,ab,id,kf,kw,sh. or (quantitativ* adj5 synthes*).ti,ab,id,kf,kw. (775687)

7 exp "clinical trial (topic)"/ and review.ti,id,kf,kw,pt. (117667)

8 (integrative research review* or research integration).ti,ab,id,kf,kw. or scoping review?.ti,id,kf,kw. or (evidence adj3 review*).ti,ab,id,kf,kw. or (narrative adj (review* or synthesis)).ti,ab,id,kf,kw. (187315)

9 review.ti,pt. and (trials as topic or studies as topic).hw. (140037)

10 (meta analysis or systematic review).md. (41838)

11 ((systematic or structured or evidence or trials or studies) adj3 review*).ti,ab,id. and (evidence based practice or treatment outcomes or mental health program evaluation).sh. (12053)

12 review.pt,ti. and searched.ab. (190564)

13 or/1-12 (1342308)

14 (((child* or adolescen* or teen* or school? or youth?) not (childbirth or adult* or family or families or mother? or maternal or woman* or women* or female? or father? or men or mens or male? or relations or former)) or ((smoking or tobacco) not mental*)).ti. (2869272)

15 prevalence.ti. (373533)

16 (acute stress or adjustment disorder* or ADNOS or affective disorder* or agoraphobi* or anorexi* or anxiety or astheni* or attachment disorder* or BPD or binge eat* or binging or bipolar or body dysmorphi* or bulimi* or catatoni* or combat disorder* or compulsi* or conversion disorder* or cyclothymi* or delusion* or depersonali#ation or depressed or depression or depressive or dissociative disorder* or dyssomni* or dyspareunia* or dysphori* or dysthymi* or dystoni* or eating disorder* or EDNOS or emotional trauma or fear or health anxiety or hoarding or hypochondri* or hysteri* or medically unexplained or malingering or mania or manic or MDD or mental* or mood? or munchausen or MUPS or mutism or neurastheni* or neurotic or neuros* or obsess* or panic or paranoi* or parasuicid* or perceptual disorder* or personality disorder* or phobi* or PND or ((post-trauma* or posttrauma*) adj stress*) or psychiatr* or psychogenic or psychopathol* or psychosomatic or psychotic or psychos* or PTSD or schizo* or (self adj (injur* or harm or mutilat*)) or (sexual dysfunction* adj3 psycho*) or social anxiety or somati* or somatoform or suicid* or trichotillomani* or stalking).ti,ab,kf,kw,id. (5635366)

17 ((autonomous or assisted or sheltered or support*) adj3 accommodation).ti,ab,id,kf,kw. (1378)

18 (13 and 16 and 17) (30)

19 (18 not (14 or 15)) (29)

20 remove duplicates from 19 (17)

***************************

OVID XSearch - Social Isolation: PsycINFO <1806 to February Week 1 2020>, Journals@OVID, PsycARTICLES Full Text, Embase <1974 to 2020 Week 06>, Ovid MEDLINE(R) and Epub Ahead of Print, In-Process & Other Non-Indexed Citations and Daily <1946 to February 07, 2020>

Search Strategy:

--------------------------------------------------------------------------------

1 ((alienation or loneliness or social isolation or social network or social support or confiding relationship? or social contact? or social network or social relationship? or social capital) adj7 (acute stress or adjustment disorder* or ADNOS or affective disorder* or agoraphobi* or anorexi* or anxiety or astheni* or attachment disorder* or BPD or binge eat* or binging or bipolar or body dysmorphi* or bulimi* or catatoni* or combat disorder* or compulsi* or conversion disorder* or cyclothymi* or delusion* or depersonali#ation or depressed or depression or depressive or dissociative disorder* or dyssomni* or dyspareunia* or dysphori* or dysthymi* or dystoni* or eating disorder* or EDNOS or emotional trauma or fear or health anxiety or hoarding or hypochondri* or hysteri* or medically unexplained or malingering or mania or manic or MDD or mental* or mood? or munchausen or MUPS or mutism or neurastheni* or neurotic or neuros* or obsess* or panic or paranoi* or parasuicid* or perceptual disorder* or personality disorder* or phobi* or PND or ((post-trauma* or posttrauma*) adj stress*) or psychiatr* or psychogenic or psychopathol* or psychosomatic or psychotic or psychos* or PTSD or schizo* or (self adj (injur* or harm or mutilat*)) or (sexual dysfunction* adj3 psycho*) or social anxiety or somati* or somatoform or suicid* or trichotillomani* or stalking)).ti,ab,kf,kw,id. (57176)

2 systematic review/ or meta analysis/ or network meta-analysis/ (513932)

3 ((systematic or structured or evidence or trials or studies) and (review or overview or look or examination or update* or summary)).ti. (400036)

4 (0266-4623 or 1469-493X or 1366-5278 or 1530-440X or 2046-4053).is. (45607)

5 (systematic review? or evidence report* or technology assessment?).jw,jx. (54310)

6 (meta-analys* or meta analys* or metaanalys* or meta synth* or meta-synth* or metasynth*).ti,ab,id,kf,kw,hw. (583547)

7 ((systematic or meta) adj2 (analys* or review)).ti,id,kf,kw. or ((systematic* or quantitativ* or methodologic*) adj5 (review* or overview*)).ti,ab,id,kf,kw,sh. or (quantitativ* adj5 synthes*).ti,ab,id,kf,kw. (775687)

8 exp "clinical trial (topic)"/ and review.ti,id,kf,kw,pt. (117667)

9 (integrative research review* or research integration).ti,ab,id,kf,kw. or scoping review?.ti,id,kf,kw. or (evidence adj3 review*).ti,ab,id,kf,kw. or (narrative adj (review* or synthesis)).ti,ab,id,kf,kw. (187315)

10 review.ti,pt. and (trials as topic or studies as topic).hw. (140037)

11 (meta analysis or systematic review).md. (41838)

12 ((systematic or structured or evidence or trials or studies) adj3 review*).ti,ab,id. and (evidence based practice or treatment outcomes or mental health program evaluation).sh. (12053)

13 review.pt,ti. and searched.ab. (190564)

14 or/2-13 (1342308)

15 (1 and 14) (1712)

16 (((child* or adolescen* or teen* or school? or youth?) not (childbirth or adult* or family or families or mother? or maternal or woman* or women* or female? or father? or men or mens or male? or relations or former)) or ((smoking or tobacco) not mental*)).ti. (2869272)

17 prevalence.ti. (373533)

18 (15 not (16 or 17)) (1534)

19 remove duplicates from 18 (836)

***********************************************

Summary of search numbers – systematic reviews

|  | **Linking** | **Housing** | **Money/Employ.** | **Social Isolation** | **Family Relations** | **Victimisat./Offend.** | **Rights** |
| --- | --- | --- | --- | --- | --- | --- | --- |
| **WoS:SSCI** | 135 | 332 | 517 | 1085 | 364 | 784 | 217 |
| **SCOPUS** | 286 | 527 | 442 | 447 | 315 | 341 | 195 |
| **Embase** | 190 | 548 | 765 | 703 | 594 | 744 | 383 |
| **PsycINFO** | 136 | 336 | 596 | 763 | 546 | 660 | 292 |
| **MEDLINE** | 141 | 340 | 717 | 533 | 475 | 501 | 189 |
| **CLib:CDSR** | 16 | 28 | 43 | 16 | 33 | 30 | 8 |
| ***OvidXS** | - | 16 | - | 836 | - | - | - |
| Totals | 904 | 2127 | 3080 | 4383 | 2327 | 3060 | 1284 |
| Duplicates | 365 | 706 | 943 | 1341 | 691 | 971 | 338 |
| To Screen | **539** | **1421** | **2137** | **3042** | **1636** | **2089** | **946** |

*: An additional search was conducted on the Ovid platform, to correct a spelling mistake and to incorporate

additional terms for loneliness/social isolation.

# RCT Stage: example strategy PsycINFO

Database: APA PsycInfo <1806 to September Week 3 2020>

Search Strategy: (n=5461)

--------------------------------------------------------------------------------

[RCT Filter]

1 clinical trials.sh. (11768)

2 (randomi#ed or randomi#ation or randomi#ing).ti,ab,id. (88340)

3 (RCT or at random or (random* adj3 (administ* or allocat* or assign* or class* or control* or crossover or cross-over or determine* or divide* or division or distribut* or expose* or fashion or number* or place* or recruit* or split or subsitut* or treat*))).ti,ab,id. (104712)

4 ((control* adj5 (trial or study or group?)) and (placebo or waitlist* or wait* list* or ((treatment or care) adj2 usual))).ti,ab,id,hw. (22391)

5 trial.ti,id. (35922)

6 controlled trial.ab. (19786)

7 or/1-6 (155879)

8 ((children? or kids or adolescen* or teens or teenagers or schools or school based) not (adult* or family or families or mother? or woman* or women* or female? or father? or men or mens or male? or relations or former)).ti. (319590)

9 prevalence.ti. (19345)

10 (7 not (8 or 9)) (144005)

11 limit 10 to yr="2000 -Current" (120238)

*[Serious Mental Illness]*

12 BIPOLAR DISORDER/ or bipolar i disorder/ or bipolar ii disorder/ or cyclothymic disorder/ or mania/ or hypomania/ or exp dissociative disorders/ or PERSONALITY DISORDERS/ or antisocial personality disorder/ or avoidant personality disorder/ or borderline personality disorder/ or dependent personality disorder/ or histrionic personality disorder/ or narcissistic personality disorder/ or obsessive compulsive personality disorder/ or paranoid personality disorder/ or passive aggressive personality disorder/ or schizoid personality disorder/ or schizotypal personality disorder/ or PSYCHOSIS/ or exp acute psychosis/ or affective psychosis/ or exp alcoholic psychosis/ or capgras syndrome/ or chronic psychosis/ or postpartum psychosis/ or reactive psychosis/ or schizophrenia/ or acute schizophrenia/ or catatonic schizophrenia/ or paranoid schizophrenia/ or process schizophrenia/ or schizoaffective disorder/ or schizophreniform disorder/ or undifferentiated schizophrenia/ or delusions/ or schizotypy/ or toxic psychoses/ (172360)

13 (bipolar or cyclothymi* or mania or manic or hypermani* or rapid cycling or conversion disorder* or (dissociative adj (amnesi* or fugue* or disorder*)) or borderline state? or catatoni* or character disorder* or delusion* or capgras syndrom* or diogenes syndrom* or depersonalization or depersonalisation or de-personalization or de-personalisation or perceptual disorder* or personality disorder* or BPD or paranoi* or psychiatr* or psychopathol* or psycho-pathol* or psychotic* or psychosis* or psychoses* or schizo* or hebephreni* or serious* mental* or SMI).ti,ab,id. (491991)

14 (12 or 13) (499282)

15 (11 and 14) (16113)

*[Common Mental Disorders]*

16 MENTAL DISORDERS/ or anhedonia/ or neurosis/ or ACUTE STRESS DISORDER/ or adjustment disorders/ or attachment disorders/ or disinhibited social engagement disorder/ or posttraumatic stress disorder/ or complex ptsd/ or desnos/ or acute stress disorder/ or post-traumatic stress/ or traumatic neurosis/ or *emotional trauma/ or AFFECTIVE DISORDERS/ or disruptive mood dysregulation disorder/ or dysthymic disorder/ or seasonal affective disorder/ or major depression/ or anaclitic depression/ or endogenous depression/ or late life depression/ or postpartum depression/ or reactive depression/ or recurrent depression/ or treatment resistant depression/ or premenstrual dysphoric disorder/ or ANXIETY DISORDERS/ or generalized anxiety disorder/ or exp obsessive compulsive disorder/ or panic attack/ or panic disorder/ or exp phobias/ or trichotillomania/ or phobias/ or acrophobia/ or agoraphobia/ or claustrophobia/ or ophidiophobia/ or social phobia/ or separation anxiety disorder/ or mutism/ or elective mutism/ or EATING DISORDERS/ or anorexia nervosa/ or binge eating disorder/ or bulimia/ or SELF-INJURIOUS BEHAVIOR/ or self-destructive behavior/ or self-inflicted wounds/ or self-mutilation/ or self-poisoning/ or suicide/ or attempted suicide/ or suicidality/ or suicidal ideation/ or suicide prevention/ or SOMATOFORM DISORDERS/ or body dysmorphic disorder/ or exp conversion disorder/ or exp factitious disorders/ or hypochondriasis/ or exp hysteria/ or neurasthenia/ or neurodermatitis/ or somatization disorder/ or somatoform pain disorder/ or munchausen syndrome/ or munchausen syndrome by proxy/ (388562)

17 "Depression (Emotion)"/ (25667)

18 "Stress and trauma related disorders"/ (19)

19 Mental Health/ or (mental* adj2 (health* or ill*)).ti,ab,id. (233745)

20 Mental Health Services/ or Community Mental Health Services/ or Community Counseling/ or Community Psychiatry/ (42727)

21 Mental Health Program Evaluation/ (2140)

22 (acute stress or adjustment disorder* or ADNOS or affective disorder* or agoraphobi* or anorexia nervosa or anxiety or astheni* or attachment disorder* or binge eat* or binging or body dysmorphi* or bulimi* or combat disorder* or obsessive or compulsi* or OCD or depression or depressed or depressive or dyssomni* or dyspareunia* or dysphori* or dysthymi* or dystoni* or eating disorder* or EDNOS or emotional trauma or fear or health anxiety or hoarding or hyperactivity or hypochondri* or hysteri* or medically unexplained or malingering or MDD or common mental or (mental* adj2 (health or well*)) or mood or moods or munchausen or MUPS or mutism or neurastheni* or neurotic or neuros* or panic or phobi* or PND or ((post-trauma* or posttrauma*) adj stress*) or psychogenic or psychosomatic or PTSD or (self adj (injur* or harm or mutilat*)) or psychosexual or (psychological adj3 sexual adj3 dysfunction*) or social* anxi* or somati* or somatoform or suicid* or parasuicid* or trichotillomani*).ti,ab,id. (901309)

23 or/16-22 (976029)

24 (11 and 23) (40144)

*[Linking Domian]*

25 social prescribing.mp. (43)

26 ((chang* or develop* or enhanc* or initiative? or intervention? or program* or mitigat* or address* or improv* or target*) adj3 (community or living or social) adj3 (condition? or circumstance?)).ti,ab,id. (1265)

27 ((communit* or social) adj (connect* or engagement? or link* or referral? or intervention? or wellbeing)).ti,ab,id. (11030)

28 "sense of belonging".ti,ab,id. (2930)

29 or/26-28 (15112)

30 (15 and 29) (43)

31 (24 and 29) (196)

*[Housing]*

32 housing/ or assisted living/ or group homes/ or shelters/ (8407)

33 residential facilit*.ti,id. (703)[^[i]^](applewebdata://4839BB90-BAA1-46A9-9E46-C662B749B28B#_edn1)

34 homeless/ or homeless mentally ill/ or deinstitutionalization/ (9459)

35 ((chang* or develop* or enhanc* or initiative? or intervention? or program* or mitigat* or address* or improv* or target*) adj3 (housing or neighbo?rhood?)).ti,ab,id. (3922)

36 homeless*.ti,ab,id. (11038)

37 ((housing adj (first or stability or instability)) or permanent housing).ti,ab,id. (871)

38 housing.ti. or ((housing adj (strateg* or polic* or project* or program* or quality)) or new* buil* or social housing*).ti,ab,id. (4887)

39 independent living programs/ or living arrangements/ or residential care institutions/ (13677)

40 halfway houses/ or independent living programs/ or living arrangements/ or residential care institutions/ (13959)

41 poverty areas/ or social environments/ (7975)

42 ((autonomous or assisted or sheltered or support*) adj3 (housing or accommodation or dwelling?)).ti,ab,id. (1551)

43 (((clubhouse or club house) adj model?) or ((autonomous or independent or assisted) adj living)).ti,ab,id. (4322)

44 (community residences or group homes or community living or supervised apartments).ti,ab,id. (3127)

45 therapeutic social clubs/ (148)

46 ((independ* or assist* or support* or secur* or sustain* or maint*) adj3 (tenanc* or tenure?)).ti,ab,id. (205)

47 ((halfway or satellite) adj (dwelling? or home? or house?)).ti,ab,id. (474)

48 (neighbo?rhood? adj (characteristic* or intervention* or program*)).ti,ab,id. (1208)

49 ((environment* or housing or neighbo?rhood?) and infrastructure).ti,ab,id. (1837)

50 built environment/ or urban planning/ (1568)

51 or/32-50 (53888)

52 15 and 51 (233)

53 24 and 51 (486)

*[Money]*

54 MONEY.ti,id. (3569)

55 socioeconomic status/ or "income (economic)"/ or budgets/ or economic security/ or financial strain/ (34235)

56 exp employee benefits/ (5804)

57 *disadvantaged/ or *social deprivation/ (6197)

58 ((access* or improv* or manag* or supplement*) adj2 (cash or money or financ* or income? or savings)).ti,ab,id. (3870)

59 exp income level/ (14774)

60 ((financial adj (autonomy or security or insecurity)) or loans or borrowing or budgeting or microcredit or microfinance or social fund*).ti,ab,id. (4345)

61 high poverty.ti,ab,id. or poverty.ti. (5274)

62 ((address* or escap* or improv* or support* or target*) adj2 (depriv* or poor or poverty)).ti,ab,id. (2074)

63 "out of poverty".ab. (179)

64 (((food or fuel) adj poverty) or food bank?).ti,ab,id. (115)

65 ((alleviat* or ease or manag* or prevent* or reduc* or stop*) adj2 (poverty or ((economic or financial) adj hardship?))).ti,ab,id. (1297)

66 ((alleviat* or eas* or manag* or prevent* or reduc* or relief or stop*) adj1 debt?).ti,ab,id. (90)

67 debt?.ti,id. (877)

68 (austerity or recession?).ti,ab,id. (3049)

69 (((basic or minimum) adj3 (wage? or income?)) or zero hours).ti,ab,id. (514)

70 paid work.ti,ab,id. (1511)

71 "dealing with money".ab. (16)

72 (family adj (income? or tax credit?)).ti,ab,id. (3858)

73 "welfare services (government)"/ or community welfare services/ or medicaid/ or welfare reform/ (7702)

74 welfare benefit?.ti,ab,id. (400)

75 or/54-74 (88399)

76 (15 and 75) (129)

77 24 and 75 (470)

*[Employment]*

78 employment status/ or employability/ or occupational tenure/ or occupational status/ or job security/ or job search/ or supported employment/ or vocational rehabilitation/ or vocational evaluation/ or work adjustment training/ or sheltered workshops/ (28910)

79 unemployment/ or personnel termination/ or employee layoffs/ (5135)

80 ((chang* or develop* or enhanc* or initiative? or intervention? or program* or address* or improv* or target*) adj3 (employment or unemployment or unemploy*)).ti,ab,id. (4855)

81 (support* adj3 (employment? or work or vocational or occupation*)).ti,ab,id. (11133)

82 ((job? or work* or employment* or employee? or occupation*) adj5 skills adj5 train*).ti,ab,id. (1202)

83 (paid adj (job? or employment or work or occupation*)).ti,ab,id. (2639)

84 (employment or unemployment or occupation*).ti. (28327)

85 individual placement?.ti,ab,id. (382)

86 ((finding or gaining or obtaining or keeping or sustaining) adj3 (work or job? or employment or occupation*)).ti,ab,id. (2621)

87 (social firms or (sheltered adj (employment or work or occupation*))).ti,ab,id. (223)

88(precar* adj1 (job? or employment or work or occupation*)).ti,ab,id. (347)

89 (voluntary work or volunteering).ti,ab,id. (3068)

90 (meaningful adj (activit* or job? or employment or work or occupation*)).ti,ab,id. (1708)

91 (((return or back) adj2 work) or absenteeism).ti,ab,id. (7117)

92 ((alleviat* or ease or manag* or prevent* or reduc* or stop*) adj ((employment or work* or occupation*) adj disabilit*)).ti,ab,id. (39)

93 (return* adj2 education).ab. (170)

94 ((education or learning or training) adj3 (access* or takeup or take up)).ti,ab,id. (4053)

95 ((labo?r force or employment or unemployment or occupation*) adj status).ti,ab,id. (2375)

96 or/78-95 (80848)

97 (15 and 96) (294)

98 limit 97 to yr="2017 -Current" (75)

99 (24 and 96) (592)

*[Family]*

100 family relations/ or intergenerational relations/ or exp marital relations/ (54189)

101 family conflict/ or marital conflict/ (5714)

102 home environment/ or living alone/ (10146)

103 ((family or families or intergenerat* or inter-generat*) adj (relation* or conflict?)).ti,ab,id. (19758)

104 ((sexual or intimate or partner? or marital) adj (relation* or conflict?)).ti,ab,id. (18862)

105 ((develop* or enhanc* or initiative? or intervention? or program* or address* or improv* or promot* or target*) adj2 relationship?).ti,ab,id. (18035)

106 ((carer? or partner or relationship? or marital) adj support*).ti,ab,id. (1485)

107 (child* adj2 (access or contact or custody or maintenance)).ti,ab,id. (5001)

108 child custody/ or joint custody/ or child visitation/ or divorce/ or family reunification/ or living arrangements/ (13740)

109 ((care proceeding? or family court? or child removal or fostercare or foster care) and (parent* or mother? or father?)).ti,ab,id. (3433)

110 parenting/ or parental involvement/ or parental role/ (21036)

111 *parents/ or parent* outcome?.ti,ab,id. (22119)

112 or/100-111 (157967)

113 (15 and 112) (182)

114 (24 and 112) (854)

115 parent* mental health.ti,ab,id. (848)

116 (114 or 115) (1675)

*[Victimisation/Offending]*

117 (VICTIMIZATION or victimisation or revictimi#ation).ti,ab,id,hw. (29460)

118 crime victims/ (4821)

119 (crime victim? or revictimi*).ti,ab,id. (1946)

120 ((victim* or crime?) and survivor*).ti,ab,id,hw. (4598)

121 domestic violence/ or battered females/ or exposure to violence/ or intimate partner violence/ or physical abuse/ or exp sexual abuse/ or shelters/ (52485)

122 elder abuse/ (1687)

123 ((domestic or partner? or spouse?) adj3 (abus* or violen*)).ti,ab,id. (21400)

124 ((domestic or marital or partner? or spous*) adj3 (rape or sex* assault*)).ti,ab,id,hw. (685)

125 (intimate partner adj2 abus*).ti,ab,id. (597)

126 interpersonal control/ or coercion/ (13686)

127 coercive control.ti,ab,id. (292)

128 ((female? or women?) adj (refuge? or shelter?)).ti,ab,id. (349)

129 (exploitation or safe guarding or safeguarding).ti,ab,id. (8021)

130 slavery/ or human trafficking/ or *freedom/ (3168)

131 or/117-130 (103206)

132 exp *criminal offenders/ (14669)

133 Recidivism.ti,id,sh. (6475)

134 ((crime? or criminal* or offend* or offence? or recidiv*) adj3 (initiative? or intervention? or program* or mitigat* or address* or rehabilitat*)).ti,ab,id. (5629)

135 ((crime? or criminal* or offend* or offence? or recidiv*) adj3 (diver* or prevent*)).ti,ab,id. (3461)

136 prisoners/ or criminal rehabilitation/ or reintegration/ (14068)

137 ((inmate? or prison* or convict? or felon? or gang member? or delinquent? or justice-involved or perpetrator? or probation) adj5 (release or integration or reintegrate or re-integrat* or rehabilitat* or re-habilitat* or desistance)).ti,ab,id. (2183)

138 (community adj2 (reentry or re-entry or rehabilitat* or re-habilitat*)).ti,ab,id. (1503)

139 or/132-138 (38230)

140 (15 and (131 or 139)) (303)

141 (24 and (131 or 139)) (837)

*[Rights]*

142 human rights/ or exp civil rights/ or exp freedom/ (33925)

143 (((citizen? or civil* or human or legal or social or voting) adj rights) or social justice or equal protection or social protection).ti,ab,id. (21843)

144 *needs/ (5277)

145 rights.ti,hw. (13944)

146 *government policy making/ (15017)

147 ((public or social) adj polic*).ti,ab,id. (19063)

148 equity-focus*.ti,ab,id. (73)

149 *health disparities/ (6331)

150 ((social or community or neighbo?rhood?) adj3 (equit* or inequit* or inequalit* or dispar*)).ti,ab,id. (6945)

151 digital divide/ or information literacy/ (818)

152 internet access.ti,ab,id. (1053)

153 (digital adj (inclusion or exclusion or divide or disparit* or equit* or inequit* or inequalit*)).ti,ab,id. (1170)

154 or/142-153 (97209)

155 (15 and 154) (79)

156 (24 and 154) (232)

*[Loneliness/Social Isolation]*

157 SOCIAL ISOLATION/ (7390)

158 loneliness/ (4680)

159 (loneliness or lonely).ti,ab,id. (11014)

160 social isolation.ti,ab,id. (7470)

161 ((social* or societ* or communit*) adj3 (isolated or isolation)).ti,ab,id. (10023)

162 ((alleviat* or ease or manag* or mitigat* or prevent* or overcom* or reduc* or stop*) adj2 (isolation or isolated)).ti,ab,id. (858)

163 ((address* or enhanc* or improv* or increas* or promot* or target*) adj2 (inclusion or inclusivity)).ti,ab,id. (1297)

164 or/157-163 (25889)

165 limit 164 to yr="2017 -Current" (5016)

166 (15 and 165) (23)

167 (24 and 165) (119)

*[Social Participation]*

168 SOCIAL PARTICIPATION.mp. (3319)

169 (social alienation or social inclusion).ti,ab,id. (2588)

170 (community involvement or social support or social network or psychosocial environment or psychosocial rehabilitation).ti,id,hw. (52837)

171 abandonment/ or alienation/ (2990)

172 (abandonment or alienation).ti,id. (3334)

173 ((social or societ* or community) adj (confine* or contact or contacts or connect* or inclusion or network* or participation or relations*)).ti,id,hw. (33174)

174 ((social* or societ* or communit*) adj3 (alienat* or discriminat* or excluded or exclusion)).ti,ab,id,hw. (10610)

175 ((alleviat* or ease or manag* or mitigat* or prevent* or overcom* or reduc* or stop*) adj2 (exclusion or excluded)).ti,ab,id. (236)

176 (social capital or social* mobil*).ti,ab,id,hw. (11532)

177 (navigator or navigators).ti,id. (261)

178 peer? support*.ti,id. (1668)

179 Peers/ and Social Support/ (731)

180 (anti-stigma* or ((intervention* or alleviat* or prevent* or reduc* or stop*) adj2 stigma*)).ti,ab,id. (3386)

181 (social learning theory or (social adj3 interaction* adj3 (counsel* or educat* or intervention* or program* or therap* or train*)) or SCIT).ti,ab,id. (3546)

182 community integration.mp. (1416)

183 (peer? adj (support* or navigat*)).ti,id. (1682)

184 community involvement/ or *social support/ or *social networks/ (40906)

185 ((social or societ* or psychosocial) adj support*).ti,id,hw. (39488)

186 ((social* or societ* or communit*) adj network*).ti,id,hw. (23139)

187 ((address* or enhanc* or improv* or increas* or promot* or target*) adj3 (social* or communit*) adj3 (network? or support)).ti,ab,id. (3652)

188 Psychosocial Rehabilitation/ (4147)

189 ((address* or enhanc* or improv* or increas* or promot* or target*) adj2 (inclusion or inclusivity)).ti,ab,id. (1297)

190 or/168-189 (114060)

191 (15 and 190) (401)

192 (24 and 190) (1118)

193 (30 or 52 or 76 or 98 or 113 or 140 or 155 or 166 or 191) (1313) [SMI]

194 (31 or 53 or 77 or 99 or 116 or 141 or 156 or 167 or 192) (5043)[CMD]

195 (**193 or 194)** (5461)

***************************

Summary of search numbers – PsycINFO-RCTs

- Linking-CMD n=196
- Linking-SMI n=43
- Employment-CMD n=592
- Employment-SMI n=75
- Family-CMD n=1675
- Family-SMI n=182
- Housing-CMD n=486
- Housing-SMI n=233
- Money-CMD n=470
- Money-SMI n=129
- Rights-CMD n=232
- Rights-SMI n=79
- Social Isolation-CMD n=119
- Social Isolation-SMI n=23
- Social Participation-CMD n=1118
- Social Participation-SMI n=401
- Victimisation/Offending-CMD n=837
- Victimisation/Offending-SMI n=303

TOTAL (OR) =5461

De-duplicated (against all previous search results, RCTs, SRs) n=3460

***************************************************************************

A copy of all the search strategies can be obtained from the researchers on request.

***************************************************************************
