# Supplementary material for: Interventions to improve social circumstances of people with mental health conditions: a rapid evidence synthesis
Source: BMC Psychiatry. 2022 Apr 28;22:302. doi: 10.1186/s12888-022-03864-9 (PMC9047264; doi:10.1186/s12888-022-03864-9)
Supplement: Supplementary file 2 — Additional file 2. Outcome Hierarchy. The tool used to select which outcomes to extract when multiple items for each outcomes were reported. [file 12888_2022_3864_MOESM2_ESM.docx]

**Appendix 2: Preferred outcome hierarchy**

**Social Needs Review - Proposed outcomes to extract**

**Social outcomes**

*Within each domain, we will extract one outcome per research question (where available). Where more than one outcome is listed for a research question, these will be used as a hierarchy: the reported outcome nearest the top of the list will be extracted*

**Employment**

Finding paid employment

1. Employment rate/work status (competitive employment vs other statuses)/Odds of competitive employment (D)
2. Time to employment (C)

Retention of paid employment

1. N no longer employed at FU (D) (include participants who did not get a job, if these are excluded from the N reported)
2. Total weeks worked (C)
3. Total days worked
4. Total hours worked
5. Total months worked

Length of sickness absence for mental health problems from paid employment

1. N returning to work (D)
2. Length of sickness absence (C)
3. Time to return to work (C) (from baseline? From start of absence? Make clear in extraction)
4. Incidence of absence (D)
5. Time to sickness absence (C)

Access to/completion of education courses or qualifications [No outcomes come across so far]

1. Engagement in education (any) (D)
2. Completed educational qualification (D)

Engagement in meaningful activity/apart from paid work [No outcomes come across so far]

1. Hours per week in meaningful activity (C)

**Offending**

Offending/reoffending

1. Criminal activities – N participants engaging in any crime during follow-up (D)
   1. N convictions during FU
   2. N self reported criminal activity during FU
2. Number of new criminal activities/felonies/convictions during follow-up (C)
3. Incarcerations (whether participants stayed in prison at any time during follow-up). (D)
4. Number of days spent in prison (C)

Successful community living following criminal conviction or prison (See outcomes for the other social domains)

**Social isolation**

Subjective social isolation

1. loneliness (C)
2. Perceived social support (C)
3. Social network quality (C)

Objective social isolation

1. Social network size (C)
2. Frequency of contact with network members (D)

Social capital

1. Individual social capital (C)
2. Perceived neighbourhood social capital (C)

**Housing**

Achieving/sustaining housing

1. Housing status at follow-up (homeless vs sheltered/supported housing vs independent living) (D)
2. Number of days/nights housed/homeless during follow-up period NB. Many report this as a percentage (C/D)
3. Days stably housed – v. similar to above, commonly used (defined as living in one’s own room, apartment, or house, or with family, with an expected duration of residence of 6 months or more or tenancy rights) (C)

Housing quality

1. Quality of housing/living arrangements, satisfaction with housing (most likely rating scales) (C)

**Mental health**

1. ONE Symptom measure of the primary disorder (if available and clear)
   1. If CMD unspecified, favour depression over anxiety
   2. SMI- psychotic symptom severity
2. OR if not, A global functioning measure

Also extract hospital/service use if reported:

- - Hospital admission during follow up (Y/N) (any admission for a MH problem)
  - Number of hospital admissions during follow up

**Quality of life**

1. Quality of life (C)
2. Life satisfaction (C)
3. Well-being (C)

**Costs**

Extract all if available of costs broken down into

- Healthcare costs
- Social costs
- Broader societal costs (costs associated with lost work etc)
- Criminal justice costs
