# Supplementary material for: Interventions to improve social circumstances of people with mental health conditions: a rapid evidence synthesis
Source: BMC Psychiatry. 2022 Apr 28;22:302. doi: 10.1186/s12888-022-03864-9 (PMC9047264; doi:10.1186/s12888-022-03864-9)
Supplement: Supplementary file 3 — Additional file 3. TIDIER Checklist. Additional information regarding the interventions in each study reported according to the TIDIER checklist. [file 12888_2022_3864_MOESM3_ESM.docx]

Appendix 3: Characteristics of included interventions

| Study ID | Domain | Treatment comparison | Why: Rationale, theory, or goal | What: Materials | What: Procedures | Who Provided | How |
| --- | --- | --- | --- | --- | --- | --- | --- |
| Aubry 2016 | Housing | Housing First + ACT vs TAU | NR | NA | Participants contributed 30% of their income toward rent, and subsidies covered the difference. Housing units consisted mostly of private-market scattered-site units. Clients were assisted to choose among available units and furnish and move into them. Study participants had to agree to observe the terms of their lease and to be available for at least one weekly visit by ACT staff. | NR | NR |
| Bejerholm 2017 | Employment | Individual enabling and support vs TAU | To increase employment rates and better vocational outcomes in people with mental illness and is based on principles from the individual placement and support (IPS) model. | NR | Phases of 1) enabling mobilization of motivational, cognitive and lifestyle strategies 2) completion of a career profile and plan 3) job-seeking 4) supported employment phase during which mobilized strategies are intertwined.  There are 10 IES principles: handling change and developing motivational and cognitive strategies 2) having a time-use pattern that supports work-life balance 3) integration of IES with mental health treatment 4) competitive employment as a primary goal 5) eligibility based on client choice 6) rapid job search 7) job search based on personal preferences 8) ongoing support and work accommodations 9) benefit counselling at an early stage and 10) systematic recruitment and quality engagement with employers. Principles 2-10 are based on the IPS model. | Two full-time employment specialists experienced in vocational rehabilitation. Employment specialists work closely with the participant in relation to the outpatient team, family, social insurance agency, public employment service and employers. They received training in motivational interviewing from a certified motivational interviewer, cognitive strategies from a CBT psychologist, time-for-work strategies from an occupational therapist and supported employment from supported employment specialists. | Face to face, Individual |
| Bell 1993 | Employment | paid work vs unpaid work | Based on a comprehensive review of vocational rehabilitation efforts, Bond (4) concluded that patients clearly prefer early placement and have an aversion to prevocational programs that provide no pay. | NR | Subjects assigned to either condition were offered jobs at a variety of work sites at the VA medical centre (for example, medical records, grounds and maintenance, patient escort, and mail delivery). Subjects in both groups could choose from more than 25 different types of work activity, and research staff made every effort to make a good match between the subject’s interests and abilities and the work assignment. All those who chose to work were successfully placed within two weeks of their intake interview. A few subjects chose to work in volunteer settings in their communities. Most worked alongside regular hospital employees; supervision was provided by the hospital departments to which patients were assigned. | Worked alongside regular hospital employees and supervision was provided by the hospital departments to which patients were assigned | Face to face |
| Bell 2003 | Employment | Paid work plus behavioural intervention vs paid work only | Evidence suggests benefits of feedback and goal setting to improve work performance | Work behaviour inventory used to provide feedback | In a 60-minute, weekly group, usually with 6 participants, workers received WBI feedback including their scores on each scale and particular items that might have been responsible for lowering or raising their score. A graphic representation of WBI ratings with longer lines indicating better scores on each subscale was also presented to the worker. After discussion and group problem-solving, a specific work performance goal was set for the next two-week period. The worker wrote the goal on a time sheet that the worker kept for recording daily work hours. At each group meeting, the worker was asked about efforts toward meeting the goal. When that goal was met, the worker would set a new goal. In this manner, WBI feedback was fully utilized to promote specific work performance changes and to recognize improvement | Masters or doctoral level staff at the VA medical centre. No training mentioned | Individual workplace support Group feedback sessions |
| Bell 2005 | Employment | Work therapy + neurocognitive enhancement vs work therapy | cognitive impairment may be one of the most influential of barriers to vocational success for people with schizophrenia. | Work behaviour inventory used to provide feedback.  As part of neurocognitive enhancement therapy, feedback was also given via the vocational cognitive rating scale | Work therapy: 1) Payment for work activity at the rate of $3.40/h for up to 15 h/weeks with increasing bonus pay for 16 to 20 h 2) job placement at the VA medical centre 3) workers meeting offering support, problem solving, detailed work performance feedback using the WBI and goal setting 4) a job coach for job related difficulties and vocational counselling 5) referral to other vocational services  Neurocognitive enhancement therapy: 1) feedback from the vocational cognitive rating scale in the workers meeting 2) cognitive exercises for up to 5 h a week for 26 weeks 3) weekly social processing group where one subject each week prepared an oral presentation with staff assistance that was delivered to the group, with questions and feedback from the other group members. Topics were "my job", "a day at work" and "what I've learnt". | NR | Individual workplace support Group feedback sessions |
| Bell 2008 | Employment | neurocognitive enhancement + vocational rehabilitation vs vocational rehabilitation only | NR | Work behaviour inventory used to provide feedback. As part of neurocognitive enhancement therapy, feedback was also given via the vocational cognitive rating scale | 1) Employment services provided by the community mental health centre, and weekly groups led by research staff including a work support group and a lifestyles group 2) The CMHC vocational program is a hybrid transitional and supported employment program that derives its essential features from the Individual Placement and Support model (IPS) plus transitional funds to facilitate rapid job placement in community-based sites. 3) Also received up to 10 h per week of computerized cognitive exercises and attended two cognitively focused, research staff-led groups per week focused on work feedback and social information-processing. Job specialists attended the work feedback group and provided participants with specific feedback from WBI and VCRS evaluations which was then used to formulate individual weekly work goals. | Doctoral level Clinical psychologists | Individual workplace support Group feedback sessions |
| Bell 2018 | Employment | vocational rehabilitation + cognition remediation vs vocational rehabilitation + cognitive games | Recent reviews of cognitive remediation (CR) for people with schizophrenia have found moderate effect sizes on neurocognitive outcomes, and there has been growing evidence for generalized benefits to social role functioning when paired with rehabilitation interventions | Individuals had free access to all available vocational rehabilitation services. | As part of the vocational rehabilitation program, VACHS and CMHC had a range of work services such as transitional programs and IPS. The transitional programs included paid placement in work therapy at VACHS or at a psychosocial clubhouse with staff support and guidance for job-seeking. Participants performed exercises that targeted auditory and visual discrimination and memory. After a short time, most participants could work through the exercises with minimal assistance. If a participant became sleepy or frustrated during a task, the monitor suggested taking a break before continuing. The monitor, however, did not coach participants in strategies to improve performance. Participants were encouraged to attend 5 laboratory sessions per week, with exercise selection and duration of sessions determined by software. Training progress was logged into a laboratory manual and reviewed with participants at each session. The “Cog Lab” did not include any specific protocol for encouraging socializing or for “buddying up” among the participants; the environment was friendly but business-like, and each person trained on their own. | Vocational rehabilitation specialists led the goal-setting groups.  A laboratory monitor provided a friendly and encouraging environment, recorded training activity, and gave technical assistance as needed | Face-to-face, virtual, individual and group-based. |
| Beutel 2005 | Employment | Occupational training integrated into psychodynamic treatment vs TAU | Based on a psychodynamic approach so the treatment is multimodal including group, individual and body-oriented psychotherapy; as well as physical training, relaxation and creative therapies. | NR | Vocational treatment is integrated into psychosomatic treatment. 1) Indication is based on intensive medical, psychological assessment and an occupational history 2) Treatment is multimodal 3) Daily working hours in a local company increase gradually from 4 up to a maximum of 8 hours a day 4) Experiences at the workplace are discussed in an ongoing group. Feedback and assessment sessions with supervisors and social workers are scheduled in the middle and at the end of the programme. 5) Assessments and work experiences enter the concluding clinical evaluation and recommendations for further occupational rehabilitation. The work assignments to a local company considers capacities, skills and interests of the patient and includes both white-collar and blue-collar jobs where the patient undergoes a formal application procedure. The daily working hours increase gradually from 4 to a maximum of 8 hours a day. Ongoing experiences at work are discussed per week and formal feedback/assessment sessions are given. | Psychotherapist and social worker. | NR |
| Boevink 2016 | Social Isolation | TREE Recovery programme + TAU vs TAU | The programme enables its participants to exchange experiences and offer mutual support. It also encourages them to develop knowledge and to use such knowledge by making it available to others. In addition, the programme promotes user-led change within mental health care organisations in the direction of a more recovery-based orientation | NA | i) Self-help working groups: Each group consisted of a maximum of eight participants plus two peer workers who acted as facilitators. The workshop activities were based on recovery and empowerment and organised as a self-help rather than a therapeutic group activity.  ii) One-day training course: The seminar programme targeted patients receiving long-term care and their professional mental health care workers. They could only take part if they came as a pair (patient and professional). Mental health care managers facilitated the professionals to attend the seminar during working hours as part of the internal education programme. The aim of the seminar was to familiarise participants with the meaning of recovery and to teach them to apply these values to their own lives (users), as well as to the mental health system (professionals).  iii) Training course: “Making a start with recovery” was a familiarisation course on the meaning of the concept of “recovery” for patients using long-term psychiatric care. | Senior peer workers | Face-to-face and group-based |
| Burnam 1996 | Housing | Social model residential treatment program vs no intervention | based on a social model recovery approach which combined elements of substance abuse recovery and mental illness management. The goal of this social model approach is to assist clients in developing an independent life in the community through abstinence from alcohol and street drugs and by enhancing their social and vocational abilities. | NA | abstinence from drugs or alcohol was a requirement for remaining in the residential community and a single infraction confirmed with drug testing resulted in expulsion from the program.  Common activities included: (1) curriculum-based groups focused on substance abuse and mental health education and rehabilitation (2) 12-step programs including participation in community-based AA or NA meetings (3) process-oriented groups to facilitate discussion of issues of importance to the clients (4) individual counselling and case-management (5) psychiatric consultation and ongoing medications management (6) general community activities including doing chores, helping with meal preparation, participating in sports and recreational activities, and personal time. | NR | Face to face with some group activities |
| Burnam 1996 | Housing | Social model non-residential treatment program vs no intervention | based on a social model recovery approach which combined elements of substance abuse recovery and mental illness management. The goal of this social model approach is to assist clients in developing an independent life in the community through abstinence from alcohol and street drugs and by enhancing their social and vocational abilities. | NA | clients were not allowed to attend the program on any day that they were discernibly intoxicated on alcohol or drugs, but staff continued to work with these clients to engage them in the program and encourage their sobriety, irrespective of number of relapses. received much more case management than residential clients  Common activities included: (1) curriculum-based groups focused on substance abuse and mental health education and rehabilitation (2) 12-step programs including participation in community-based AA or NA meetings (3) process-oriented groups to facilitate discussion of issues of importance to the clients (4) individual counselling and case-management (5) psychiatric consultation and ongoing medications management (6) general community activities including doing chores, helping with meal preparation, participating in sports and recreational activities, and personal time. | NR | Face to face with some group activities |
| Castelein 2008 | Social Isolation | Guided peer support VS TAU | Aim to provide peer to peer interaction | NA | First, people are encouraged to work in pairs to exchange positive experiences from the previous two weeks (ten minutes). Next, all pairs share with the group the stories they just heard (ten minutes). Then the nurse initiates the general discussion by asking, “What have you just heard that could be of interest for the whole group?” Next, the participants choose the theme of the session (five minutes), briefly introduced by the nurse (two minutes). The themes should relate to the illness, for example: living with schizophrenia, telling others about your illness, or resuming your job. After a 15-minute break, they share their experiences about the theme in pairs (15 minutes), participants reconvene for the final plenary session (25 minutes). At the end, the nurse briefly summarizes the session (eight minutes). | Nurses - The nurse had a facilitating role to avoid professionalization of the groups. | Group face-to-face - nurses needed to guide the groups with minimal involvement. This required training in the intervention and the minimal guidance attitude: offering structure, continuity, and a sense of security without actively interfering with the group process. |
| Chandler 2006 | Offending | Integrated dual diagnosis treatment post-custody vs usual post-custody services | - treat mental health problems and substance abuse and reduce re-offending and return to jail | NA | Features an MDT, with a substance abuse specialist and counselling. Outreach case management approach (very little detail) | Team members had worked in substance abuse or dual diagnosis services before. And also featured a probation officer, and dedicated staff to assist housing | NR |
| Christensen 2019 | Employment | IPS with enhancements vs TAU | No details given | Employment specialists are encouraged to closely follow the methods described in the updated and expanded IPS manual “Applying the individual placement and support (IPS) model to help clients compete in the workforce”. A detailed manual based mainly on the “Thinking skills for work” manual was developed in Danish but was adapted to the present trial and extended with work-related social skills training. Use of computer for training with the CIRCUITS software | The IPS intervention is based on eight key principles: (1) eligibility based on client choice, (2) focus on competitive employment or education, (3) integration of mental health and employment services, (4) attention to client preferences, (5) benefits counselling, (6) rapid job search, (7) systematic job development, and (8) individualized long-term job support. enhanced with cognitive remediation and work-related social skills training: The enhancement program consists of 24 group-based sessions of computer training using newly developed software (CIRCuiTS) and incorporates evidence-based training principles such as errorless learning and massed practice. The computer training provides practices across a broad range of cognitive functions hypothesized to be impaired in persons with severe mental illness, including attention, concentration, psychomotor speed, learning, memory, and executive functions. Each participant works through a so-called metacognitive journey consisting of 278 task instances divided into seven different stages. The participant receives ongoing feedback and is able to monitor their own scores, strategy use, progression in skills, and development or change in personal goals. In addition to receiving the computer training, participants are offered 12 sessions in coping strategies for dealing with cognitive challenges. These sessions are aimed at helping participants develop effective strategies for improving their cognitive skills or reducing the effects of cognitive challenges in order to achieve vocational goals, maintain work, and increase performance. Finally, the program consists of six work-related social skills training sessions with a focus on disclosure, communications skills, decoding norms for social interaction, and conflict management. | the two IPS teams will be trained in the method by an IPS expert, who will also offer tele-supervision throughout the trial period. Training part: Trained psychologists will be responsible for the group sessions, and employment specialists will be co-therapists. To ensure the quality of the intervention, employment specialists are trained by psychologists with experience in using the method | Training: performed primarily in group format, and eight participants will be assigned to each group. |
| Conoley 1985 | Social Isolation | Reframing vs Waitlist control | Aim to change the client's view of the problematic behaviour (loneliness) from uncontrollable to controllable | NA | Group intervention. 1st session: Refraining subjects received directives such as the following: "Being lonely has been for most others with situations similar to yours, part of learning a new way of being. This is enabling you to be more adaptable, to function in a new, more creative way"; or "A nice part of being lonely now, is that it allows you to develop and discover more about yourself at a time when others may be so wrapped up in a relationship that they end up spending their time trying to be what someone else wants them to be"; or "Your development and personal growth is being experienced as loneliness at this time. As you are moving away from dependence on your family through this period of self-discovery, you are learning new ways to express your own uniqueness." 2nd session: reviewed the first session and explored reactions to the events of the intervening week. Again, between three and five reframing directives continued the preceding week's theme. | Two male doctoral students with 3 years counselling experience were the experimenters, each received 6 hr of training in both interventions. The experimenters were trained until they could perform confidently and accurately. | Group face-to-face |
| Cook 2008 | Employment | Supported employment vs TAU | Supported employment appears to be effective for individuals with schizophrenia spectrum disorders. What is not well understood is the model’s differential effectiveness for those with schizophrenia versus other severe disorders in diverse populations, multiple locales, and different program structures |  | The experimental condition was always a form of enhanced best-practice supported employment.  At Maryland, Connecticut, and South Carolina the experimental condition was Individual Placement and Support, where multidisciplinary provider teams engaged in rapid job search, placement into competitive jobs, and provision of training and ongoing support. The Massachusetts site used the Program of Assertive Community Treatment vocational model, with services provided exclusively in the community through a team of psychiatrists, nurses, case managers, and vocational specialists who placed patients in competitive employment and provided continuous employment support.  The Texas, Maine, and Arizona sites used experimental models developed especially for the EIDP. The Texas model included supported employment services with social network enhancements designed to create more balanced and reciprocal interpersonal networks.  In Maine, the model used family-aided assertive community treatment teams working with an employer consortium of the area’s major businesses to develop job opportunities and workplace supports. Arizona’s integrated treatment team was comprised of psychiatrists, case man- agers, rehabilitation counsellors, employment specialists, job developers, and benefits specialists emphasizing rapid job placement and ongoing support for job retention | NR | NR |
| Cosden 2005 | Offending | Mental health treatment court vs TAU | MHTC (non-adversarial court proceedings) with the aim to provide offenders with mental illnesses with the opportunity for treatment instead of jail time, with the hope of decreasing the recidivism of this population. ACT - community approach to manage serious mental health problems and help community re-integration | NA | MHTC - Once offenders entered the program, decisions regarding their disposition were made by the treatment team, which met before each court session to discuss the participant’s progress. Offenders were scheduled for weekly or bi-weekly court supervision. The judge spoke with the offender during each session and gave the offender positive feedback or assigned sanctions, including jail days, for non-compliance. ACT - case managers had frequent contact with their clients and offered them practical help, including transportation to meetings, attendance of skills building, community re-entry and substance abuse management groups and vocational skills training, access to section 8 housing. | MHTC & ACT: treatment team | Face-to-face management with groups |
| Cusack 2010 | Offending | Forensic ACT vs TAU | Aim to divert people with mental illness away from jail. Several features make FACT unique, including its targeting of individuals with prior arrests, accepting referrals from criminal justice agencies, recruiting criminal justice agency partners, use of court sanctions to encourage participation, engaging probation officers as members of the treatment team, and making re-arrest prevention an explicit goal | NA | Participants in the FACT program received team-based mental health and substance abuse services, as well as support for housing, employment assistance, benefits applications, and advocacy. The full-time probation officer worked with the courts and the participants to establish conditions of probation, agreement to avoid substance abuse that encouraged participation in behavioural health services. Each member of the team had primary responsibility for providing and/or coordinating services appropriate for designated consumer participants. Psychiatric and medication services were available to the participants through a part-time psychiatrist and registered nurse. | FACT was staffed and operated by the county behavioural health department. Team includes a peer support worker, nurse, psychiatrist and a probation officer | NR |
| Davidson 2004 | Social Isolation | Matched with a volunteer partner who had a personal history of psychiatric disability vs Matched with a volunteer partner who had no history of psychiatric disabilities vs Not matched with a volunteer partner | Aim to investigate the role of social support in recovery from serious mental illness. | NR | Volunteers and participants were matched by project staff based on shared interests and were asked to spend 2-4 hours per week for a period of 9 months participating together in social or recreational activities in the community. Participants were either matched with a volunteer partner with his/her own history of psychiatric disability or with a volunteer partner from the general community who did not have a personal history of psychiatric disability. | All volunteers received an initial orientation and training session and then participated with other volunteers in ongoing, monthly peer support meetings facilitated by consumer and non-consumer staff. | Face-to-face and individually. |
| Davis 2012 | Employment | IPS vs TAU | The standard VA Vocational Rehabilitation Program (VRP) does not meet the occupational recovery needs of veterans with PTSD. IPS has been shown to be effective for individuals with SMI not yet in those with PTSD. Engaging in competitive employment rather than sheltered jobs has been shown to enhance other recovery outcomes such as self-esteem and quality of life. | The ‘A working life for people with severe mental illness’ manual and an IPS manual from the substance abuse and mental health services administration were used as references for administering IPS. | IPS specialist was integrated into the clinical mental health or PTSD treatment team, carried out all phases of the vocational services provided predominantly community-based services, provided assertive engagement and outreach in community-based employment, had a caseload of no more than 25 clients, and provided continuous, time-unlimited, follow-along supports for vocational services. IPS involved rapid job search and individualised placement in diverse competitive jobs, with ongoing work-based vocational assessment and assistance in finding subsequent jobs, if needed. Group supervision is regularly scheduled with IPS specialists and an IPS supervisor | IPS specialists: The IPS specialists received ongoing training and supervision via monthly conference calls and quarterly site visits by the national IPS consultant (RT) | NR |
| Davis 2015 | Employment | Mindfulness based stress reduction (Mirrors) vs Intensive support control | Mindfulness interventions appear to be accepted by individuals who have schizophrenia, who report benefits such as improved cognition and coping, similar to those with other chronic mental and physical conditions. Enhancing vocational rehabilitation with a mindfulness stress reduction programme may enhance work persistence in those with schizophrenia, as the main reported reasons for work discontinuation include impaired cognition and coping. | Intervention based on MIRROS manual - based on Kabat-Zinn’s MBSR programme | Based on the Mindfulness Based Stress Reduction curriculum with an added, explicit emphasis on self-compassion and referring to work and schizophrenia as a source of stress that participants have in common during discussions. The weekly MBSR curriculum includes mindfulness meditation practices; experiential exercises to convey teaching points; discussion of the experience of meditating; and covering topics such as the stress response and the effect of meditation in daily life, and in particular for the current study, work. | Classes were taught primarily by the first author, who had a regular yoga and personal meditation practice for 3 years in addition to completing an MBSR class and two of three required trainings for teacher certification by the Centre for Mindfulness at the University of Massachusetts Medical School. She was assisted by doctoral-level psychology trainees who functioned primarily as co-teachers. The co-teachers’ initial training involved completing an MBSR class and observing/participating during a full cycle of the 8-week MIRRORS.  The role of co-teacher was introduced and progressively advanced until co-teachers were able to independently conduct the sessions. Ongoing training and super- vision was provided by the first author and involved maintaining a personal mindfulness practice, which included attending and/or leading one to two weekly 30-min mindfulness practices for the research team and/or medical centre staff and participating in weekly supervision | Face to face in a group of max. 8 people |
| Davis 2018 | Employment | IPS vs transitional work | Vocational rehabilitation services for veterans with PTSD typically involve stepwise transitional work assignments in Veterans Administration (VA) settings rather than a referral to individual placement and support (IPS)-supported employment. Individual placement and support has shown robust effectiveness in people living with SME. For the past decade, as a consequence of limited resources and lack of results from large effectiveness trials in a PTSD population, the VA has prioritized IPS services for veterans with serious mental illness; as such, veterans with PTSD have limited access to IPS-supported employment. | NR | IPS involves job development to rapidly obtain competitive work in the community that aligns with the participant’s preferences, skills, and abilities. The IPS specialist provides all phases of person-centre employment services that include vocational assessment; individualised job search consistent with the participant’s preferences, skills and abilities; job coaching and advocacy; care coordination within the treatment team; disability benefits counselling and open-ended follow-along supports. | The IPS specialists were hired and trained specifically for the study. Most of the IPS specialists had experience with or exposure to evidence-based supported employment serving other populations. On-site and remote IPS training and technical assistance were provided by experienced IPS trainers who guided the IPS service delivery and implementation as an integral part of the local sites’ existing IPS teams. | NR |
| de Waal 2019 | Victimisation | Self-wise, other-wise, street-wise (SOS) training + TAU vs TAU | To prevent victimisation in dual-diagnosis patients as an add-on to care as usual | There is reference to some specifically designed visual materials used in the intervention, such as emotions but no reference to where you could find them. When training the intervention providers, a treatment manual was studied. | The SOS training comprises three modules: self-wise, other-wise and street-wise - each of which consist of four sessions. Self-wise: involves a newly developed emotion regulation skills training, inspired by the principles of existing emotion regulation skills training. Comprises various interactive group exercises to practice with recognising one's own emotions, interpreting emotional expressions of others and coping with feelings of anxiety and feeling of anger. Other-wise module: Newly developed conflict resolution skills training specifically focusing on preventing and resolving interpersonal conflicts. Participants jointly compose a list of important resolution skills which are practiced in role-playing exercises categorised by relevant themes. The specific role-playing exercises are based on input from dual-diagnosis patients who participated in the pilot phase to ensure the exercises are relevant to the population and applicable to real-life situations of dual-diagnosis patients. Streetwise module - Newly developed street skills training which builds on the idea that teaching patients about behavioural factors that contribute to their risk of victimisation may be effective in reducing victimisation. | Therapists were 6 psychologists with a master's level university degree and 8 nurses with immediate or higher vocational education. They all received training to deliver the SOS training which included studying the treatment manual and participating in 2 4-hour training sessions. The first 12 sessions of SOS training per location were supervised and supervision was provided at least once a month at each location. Additional supervision by telephone or email was provided on request. | Manualised group-based, face-to-face intervention. |
| de Weerd 2016 | Employment | Work-focused CBT with convergence dialogue training vs work-focused CBT only | Aims to facilitate return to work as part of CBT and convergence dialogue meeting of employee, therapist and supervisor | Evidence-based protocols for Axis I disorders on how to conduct CBT are found in the list on references but also online via Google. | Therapists are requested to follow the specific protocol and address RTW early in treatment to reach treatment goals such as activation, day structure, social contact and self-esteem within the context of work. The CDM was performed according to a prescribed agenda: first informing the supervisor about the treatment and enabling the employee to disclose his/her experiences with treatment. Then, a dialogue between employee and supervisor was initiated to identify and solve obstacles for RTW. At the end of the CDM, employee and supervisor agreed on a joint RTW plan. | Each participating department employed 9-16 therapists. All therapists were trained and received supervision in applying evidence-based treatment protocols for Axis I disorders of the DSM-IV. Therapists were all university graduates in clinical psychology and registered as, or in training for health care psychologist. | Face-to-face and individually. |
| Elbogen 2016 | Money | $teps for Achieving Financial Empowerment ($AFE) (psychoeducational money management program) vs TAU | A psycho-educational, recovery-oriented management intervention to help veterans with psychiatric disabilities who face a unique set of challenges concerning money management | $AFE adapts various skills training materials from various sources e.g. VA, SSA, the US Department of Labor and Boston University Psychiatric Rehabilitation Practitioner Tools. | The intervention involves outlining specific strategies to help military veterans to save money e.g. by providing information about veteran discounts for goods and services. Facilitators teach veterans how to create a viable budge by first distinguishing between expense needs and expense wants and then listing their own income and expenses including saving 10% of their income for emergencies and another 10% for reaching a goal or purchasing a desired item. Facilitators calculate how much veterans could earn without losing disability SSA benefits and review various VA vocational rehabilitation programs available to veterans. Strategies for avoiding various forms of financial exploitation are reviewed and local/national/vocational mental health and veterans’ resources are provided. | Care managers who work with veterans with psychiatric disabilities | 1-1 intervention face-to-face. |
| Elison 2020 | Housing | Manualized treatment model for co-occurring mental illness and substance use disorders (MISSION-Vet) vs TAU | A manualised treatment model for cooccurring mental illness and substance abuse disorders specifically for homeless or formerly homeless veterans. A peer specialist intervention. | The MISSION-VET manuals were used as a basis for 20 structured sessions of the intervention and were found in the reference. | The intervention was guided by the adapted MISSION-Vet intervention. The peer specialist-veteran meetings were designed to focus on mental health and substance use recovery and community integration skills delivered in both 20 structured sessions derived from the MISION-Vet manuals and 20 unstructured meetings designed for community engagement and relationship building. The meetings might address emergent veteran needs e.g. medical, family, legal or benefits issues. | The peer specialists had to be veterans with significant recovery from mental health issues which could include substance abuse. 7 male peer specialists were hired, 6 were White and 1 was African American. The peer specialists were supervised weekly by psychologists and their work was guided by the adapted MISSION-Vet intervention. | Face-to-face and individually. |
| Erickson 2020 | Employment | IPS + TAU vs TAU | To increase competitive employment for people with severe mental illness | NR | Clients randomly assigned to the IPS group received 1 year of employment support from one of two experienced vocational counsellors, whose offices were located in the three hubs. Both IPS workers provided services in a range of locations, both within and outside of the mental health centres. Three of the 12 mental health centres served a substantially rural population. The vocational counsellors regularly communicated with the clinical staff, both individually and at the weekly team meetings at each hub. They also met weekly with the IPS supervisor who was responsible for all the IPS services in the health authority. | The vocational counsellors regularly communicated with the clinical staff, both individually and at the weekly team meetings at each hub. They also met weekly with the IPS supervisor. There were two experienced vocational counsellors. | NR |
| Fletcher 2008 | Housing | Integrated assertive community treatment vs standard care | Considerable research has shown that assertive community treatment (ACT) produces better outcomes than other treatments for individuals with severe mental illness, especially for housing and consumer satisfaction (Bond et al. 2001; Mueser et al. 1998). Consequently, some researchers have argued that the ideal way to serve dual disorder individuals is to combine integrated treatment with ACT. | NR | An approach to treat problems for people with dual disorders. Integrated treatment tends to emphasize a) assertive outreach b) motivational interventions c) a stages-of-treatment approach d) cognitive behavioural counselling e) interventions to strengthen social networks supportive of recovery f) a long-term perspective | A new IACT clinical team was created, although several team members had prior experience providing ACR. They received training and follow-up consultation regarding ACT treatment principles and practices. Experts provided the IACT team with training and consultation on integrated treatment principles and services. The IACT team had a substance abuse specialist on staff and provided outpatient substance abuse counselling and bi-weekly treatment groups. | NR |
| Fowler 2019 | Employment | Social recovery CBT + TAU vs TAU | Based on CBT and combines techniques of CBT with vocational case management. | Specific therapeutic procedures used in the study were drawn from existing CBT manuals such as focus on self-regulation of psychotic symptoms which were can be referenced. | Stage 1) Developing a formulation of the person in social recovery - assessments and history taking with respect to personal motivation and goals. Stage 2) Identifying and working towards medium- to long-term goals - Identifying specific pathways to meaningful new activities including referral to relevant vocational agencies or direct liaison with employers and cognitive work to promote a sense of agency and addressing hopelessness, feelings of stigma and negative beliefs. Stage 3) Active promotion of social activity, work, education and leisure linked to meaningful goals - promotion of activity by behavioural experiments while managing symptoms. Therapists were encouraged to combine therapist role with case management roles typical of IPS and encouraged to adopt a pragmatic and problem-solving approach in assisting people to overcome work-related problems. The SRCBT group also received TAU which was active case management by MDT secondary care mental health teams. | Therapy in Norfolk was carried out by case managers who had no previous formal training in CBT, but who had over 2 years’ experience working in an early intervention in psychosis team, under the supervision of expert CBT therapists. Therapy in the Cambridge based centre was carried out by CBT therapists who had attended approved courses prior to working on the trial. | Face-to-face and individually. |
| Gelpkopf 1994 | Social Isolation | Comedy films vs Variety of film genres | Humour is a powerful social facilitator. A group of people faced with humorous stimuli will laugh more easily than individuals facing such a stimulus alone. laughter helps establish group membership. | NA | The experimental group was exposed exclusively to comedies. | NR | Group face to face |
| Glynn 2004 | Social Isolation | Skills training + generalization vs skills training only | Unfortunately, the benefits accruing from psychosocial interventions in general and social skills training in particular are often impeded by obstacles to the adoption of these skills in patients’ everyday life. Some of these generalization obstacles derive from the negative symptoms, positive symptoms, and neurocognitive impairments inherent in schizophrenia. Other impediments to generalization are related to lack of environmental opportunities for using the skills learned in a clinical site and lack of encouragement and reinforcement from community supporters and caregivers. | NR | (IN BOTH GROUPS): social skills training modules that were administered in a group setting. Subjects participated in modules on medication management and symptom self-management during the first 24 weeks of the trial. A social problem-solving module was completed during the subsequent 12 weeks. Finally, a group focused on successful living skills was completed during the last 24 weeks of the intervention.   (INTERVENTION SPECIFIC): In vivo amplified skills training is a behaviourally oriented, manual-based intervention with 60 specific activities scheduled to coincide temporally with the skills being trained in the clinic, utilizing an overarching problem-solving approach embedded in ongoing assessment. In vivo amplified skills training has four objectives: 1) support completion of clinic assignments in the community 2) identify opportunities for skill use in the community 3) reinforce opportunities for skill use in the community 4) establish a liaison with or develop a natural support system to maintain gains.  Typical in vivo amplified skills training activities included going to a pharmacy to investigate remedies for medication side effects such as dry skin, dry mouth, or photosensitivity; developing a regular daily schedule; identifying the nearest medical facility for use in an emergency; and attending social gatherings to learn to identify and remedy social problems. Depending on their complexity and requirements, one or two tasks were covered in each 75-minute in vivo amplified skills training session. Sessions were held with individual subjects in the community, often in the patient’s home or in a setting conducive to the skill being trained (e.g., a coffee shop for communication skills training, a place with a phone when patients were being taught how to contact a medical worker if they were experiencing side effects). Skills were modelled and prompted by the trainer, who then provided ample social reinforcement for the participant’s rehearsal and eventual successful completion of the skill activity. | The social skills training: doctoral- and master’s-level psychologists, an occupational therapist, and a social science technician. The in vivo amplified skills trainer had a background and skill level consistent with an experienced psychiatric technician.  [training not discussed, but trainers were supervised] | Face to face, individual and group activities |
| Glynn 2017 | Employment | IPS plus work skills training vs IPS only | One strategy to improve job tenure and to empower consumers to manage their own work challenges is to augment supported employment with more focused instruction on the generic work skills required to succeed across employment settings. The Workplace Fundamentals Module (WPFM) is a manualized, clinic-based behavioural group intervention designed to increase employment tenure by providing workers with the competencies needed to be successful in any job. | Problem solving homework and out of session practice | IPS: no information given Work skills training: 7 sequential learning activities: skill introduction, videotaped demonstration, role-playing practice, resource problem solving, outcome problem solving, out-of-session practice, and problem-solving homework. These learning activities covered nine generic work skill areas, including benefits and costs of work, identifying specific job details, recognizing problematic job details, learning a seven-step method to solve problems, solving problems with symptoms and medication, coping with general medical issues and drug abuse, interacting with the supervisor to obtain feedback, understanding workplace culture and interactions on and off the job, and improving work motivation | IPS: trained IPS specialist with a BA or master’s degree Work skills training: facilitator with MA or PhD and experience of working with psychosis | Face to face, group meetings |
| Goldfinger 1999 | Housing | Group housing vs independent housing | shared housing arrangement intended to maximize independence and minimize the presumed risks of independent living. | NA | Unlike traditional group homes, the ECH model offered residents permanent secure housing without the requirement of treatment compliance. ECH staff were trained to promote resident independence, and it was expected that staff time would gradually be reduced as the residents learned how to manage their house themselves. ECH residents were encouraged to take the lead in establishing their own house rules. House staff offered advice and support in this process. In addition to fostering consumer independence, other goals were to reduce isolation, to provide paraprofessional monitoring of the residents' clinical condition, and to offer skills training in managing the house (e.g., paying the bills, negotiating with the landlord). Apartments in this demonstration project were one- or two-room single apartments in public housing subsidized by the Boston Housing Authority. In both housing types, residents were required to maintain behaviour that met landlord or co-resident agreements. All tenants paid rent, which they had not had to do in the homeless shelter. All had some form of income support, and rents (including utilities) were set as a proportion (about one-third) of that benefit amount. Each client, regardless of housing assignment, had a project funded case manager. Each case manager had a caseload of 15 clients. . | Support from case managers - Housing provided by local authority | Case manager face to face |
| Granholm 2005 | Social Isolation | Cognitive behavioural social skills training vs TAU | It is possible that combining cognitive behaviour therapy and social skills training may improve treatment. For example, by challenging thoughts that interfere with skills execution in the real world (e.g., expectancies, delusional fears), social competence and functioning may be improved. Also, by adding social skills training, emphasis on social functioning is increased in types of cognitive behaviour therapy that primarily target symptoms. | The treatment manual included a patient workbook that contained homework forms | Social skills training + CBT + aids to compensate for cognitive impairment associated with schizophrenia as well as normal ageing (e.g. challenging ageist beliefs, such as “I am too old to learn”, or age specific problem solving such as coping with hearing problems)  Self-contained modules including orientation to the group and progression through skills across sessions. Patients completed all three modules twice for a total of 24 sessions.   i) Thought-challenging module: patients used thought records and homework assignments to identify relationships among thoughts, feelings, and behaviours, and they identified mistakes in thinking. Patients conducted behavioural experiments to gather evidence to evaluate their beliefs. The primary thoughts targeted were beliefs about voices (e.g., “God is speaking,” “The voice could harm me”) and events related to delusions. To simplify learning and to help patients remember to use cognitive techniques in everyday life, mnemonic aids were provided (e.g., laminated wallet cards with “The 3Cs: Catch it—identify the thought, Check it—examine evidence, Change it.”).  ii) Asking for support module: to improve communication skills and social interactions. This training used behavioural role-playing exercises that focused on reporting symptoms to doctors, expressing positive and negative feelings, assertive sharing in social interactions, and improving everyday leisure activities.  iii) Solving problems module- problem-solving skills were taught by using the acronym “SCALE,” which represented “Specify, Consider possible solutions, Assess the best solution, Lay out a plan, and Execute and evaluate the outcome.” Problems related to illness and disability were emphasized, including coping with symptoms, stressors (e.g., loss of a loved one), taking medication, using public transportation, leisure activities, hygiene and nutrition, and getting eyeglasses and hearing aids. | Doctoral-level psychologists or senior graduate students in clinical psychology with at least master’s-level training and 2 years of clinical experience. Two therapists led each group. Two of us (E.G., J.R.M.) provided training and weekly supervision, including review of session videotapes. | Face-to-face group sessions |
| Gutman 2009 | Employment | BRDGE supported education programme vs TAU | In accordance with the model of human occupation, supported education participants can develop • An occupational identity as an adult learner, • Occupational competence in the educational and social skills needed to succeed in a postsecondary setting, and • Occupational adaptation to the demands of being an adult learner with a psychiatric disability. Through structured activities and opportunities for practice, participants can master the habits and routines needed to support the desired role as an adult learner. | NA | The Bridge Program consisted of 12 classroom–lab modules that were held twice per week over 6 weeks. Module topics included the following: • An exploration of training programs, degrees, and work options; • Study skills for school or work; • Time management skills for school or work; • Effective reading skills for school and job training; • Basic writing skills for school or job seeking; • Basic computer skills; • Introduction to Internet skills; • Basic math skills for school and job placement tests; • Use of library resources; • Public speaking strategies for school or work; • Professional behaviours and social skills; and • Stress management skills for school or work  The modules consisted of an integration of lecture and lab activities through which participants could practice the skills they were learning. | Occupational therapy students: mentors. Training not described | Computer based learning in a lab face-to-face mentoring |
| Harris 2017 | Employment | Cognitive remediation and supported employment vs internet information | To combine supported employment programs with internet-based cognitive remediation therapy to improve employment outcomes for people with SMI in frontline services. | Participants had to access all material via a purpose build website but were given initial 10-hour training. They were requested to use 4 commercially available cognitive training packages - Lumosity, Brain HQ, Mybrainsolutions and Scientific Brain Training Pro. | Participants were provided a password to access treatment and were sent an email with instructions, the information was also sent to the DES workers so they could assist participants if necessary. They were requested to use 4 commercially available cognitive training packages - Lumosity, Brain HQ, Mybrainsolutions and Scientific Brain Training Pro. Participants were not directed to any one exercise or website but suggested to sample and sue as many as they liked. | Digital-based via computer. But DES workers and research team provided assistant to participants when necessary and all DES workers were given a brief orientation to the trial and were encouraged to refer their clients to the study. | Via the internet and individually. |
| Haslam 2019 | Social Isolation | Groups 4 Health social identity intervention vs TAU | Social identity theory-derived intervention focusing on building and maintaining positive social group identifications to improve health and well-being. It targets specific social identity processes e.g. multiple group membership, group identification, group maintenance, group gain and group compatibility | The five-module program is manualized and comes with an associated workbook that has program activities and exercises as well as a facilitator's manual. Doesn’t specify where the materials can be found exactly but references both the workbook and manual. | First session - Schooling: focuses on psychoeducation and raises awareness of role of social groups.  Second session - Scoping: uses a social identity mapping tool to help people visualise their social group world. Third session - Sourcing: focuses on maximising a person's existing group relationships which are positive.  Session 4 - Scaffolding: uses the G4H group as a platform where participants build on their existing relationships to join new groups and develop a social plan.  Fifth module (Takes place one month later) - Sustaining: people have trialled their social plans and report on experiences of doing so. | Facilitated by two provisionally registered psychologists completing supervised graduate training in the psychology clinic of the researcher’s university. | Intervention was provided in groups comprising of between 5-9 participants and was delivered face-to-face. |
| Hasson-Oyayon 2014 | Social Isolation | Social cognition and interaction training + social mentoring vs social mentoring only | Social impairments associated with schizophrenia are targeted | Educational handouts, videos and slides given to participants during training (no information on where to access.) | One of three weekly social mentoring sessions was dedicated to SCIT session. Home assignments given. From cited papers also testing SCIT: structured check ins: ppts make observations about their current emotional state and its relationship to their behaviour, thoughts and social interactions. Homework. In sessions: psychoeducation, discussion of social cognitive principles, learning social cognitive strategies, using these strategies to analyse social cognitive stimuli. Strategy practice in the form of games or collaborative data gathering exercises or problem-solving sessions. | Social mentor: NR Clinician: experience of providing psychiatric rehabilitation services and had completed a two-day workshop on SCIT training. All SCIT group leaders attended monthly 2-hour group supervision sessions. | Face to face group meetings |
| Hees 2013 | Employment | Adjuvant occupational therapy vs TAU | This new OT adopts an increased focus on three elements found to be essential for a successful return to work (RTW): an early return to the work situation according to the ‘place-then-train’ principle, an increased focus on work-related coping and self-efficacy, and enhanced communication among the various stakeholders involved. | Quality of work model used as basis for group and individual discussion | Phase 1: Problem clarification intake (one session)- current work situation and problem areas explored. treatment goals and expectations examined. occupational anamnesis (three sessions)- educational and occupational history systematically analysed to identify recurrent ineffective coping patterns in stressful situations video-observation (one session)-the patient is recorded within a simulated work environment (i.e., engaging in role-playing), while the patient performs key tasks relevant to his or her job. Afterward, the recordings are viewed, and the patient's experiences regarding the current tasks, workload, and relationships with colleagues are discussed. In this way, the aspects of the job that the patient experiences as problematic are identified.  After completing the problem clarification phase, the occupational therapist discusses the content and goals of the intervention with the occupational physician (OP) by telephone.  Phase 2: Occupational intervention 8 x group sessions, 4x individual sessions. sessions based on "quality of work model" that 5 factors affect work performance - work load, autonomy, relationships at work, job perspective, work-home interference.  group sessions: QW model discussed and patients taught how to evaluate the positive and negative factors in their work situation, and decide what aspect of model is most important to change their work situation, which forms the basis of their individual work reintegration plan. progress is evaluated and preparation to meet with the employer through role playing.  Concurrently with group sessions, three individual sessions and a meeting with the employer take place. During individual sessions, the therapist tries to relate the presently occurring work stressors to the patient's recurrent ineffective coping-pattern (as discussed in the first phase of the intervention). If needed, the therapist provides help with filling out the QW model. In addition, the patient's progress with the work-reintegration plan is monitored during individual sessions. During the meeting with the patient's employer (i.e., supervisor), the occupational therapist educates the employer regarding the content of the occupational intervention and the consequences of depression for work performance. During this meeting, the patient has the opportunity to openly discuss work-related difficulties with the employer, such as an excessive workload or problematic interpersonal interactions. Phase 3: Follow-up Within four to six weeks after the completion of the occupational intervention, patients receive a follow-up session to discuss potential problems during the work resumption process | Occupational therapist: Experienced, and received extensive training in the intervention | Face-to-face, group and individual sessions |
| Hellstrom 2017 | Employment | IPS modified for people with mood and anxiety disorders vs TAU | The IPS-MA method is based on eight principles: 1) Sherpa is the patient’s advocate, not an authority or a healthcare provider; 2) the process is led by the individual’s goals and focus is on patient resources; 3) assistance is flexible, without time limits, and responsive to the needs of the patient; 4) the goal is competitive employment or education, without pre-vocational training; 5) the belief is that returning to work is possible despite a mental illness, but therapeutic recommendations in terms of postponement are acknowledged; 6) liaison with healthcare and social workers ensures a coordinated service; 7) a meaningful and realistic career plan will be developed and evaluated continuously after job start; and 8) Sherpa is an interdisciplinary team, which will be reflected in the assistance of each individual. | NA | Five basic services comprise IPS-MA: 1) Individualised mentor support based on psychiatric knowledge. Sherpa mentors all have a background as professionals in mental health services. In cooperation with the participant, the Sherpa mentor helps develop a plan of action in which resources and problems in social life as well as working life are clarified. The Sherpa mentor supports the participant in how to structure and manage everyday life, renew contact with friends and/or family, prepare important meetings and live a healthy everyday life with the disorder. The Sherpa mentors very often act as lay representatives for the participants at meetings at the local job centres or municipalities. 2) Coordination of services provided by Sherpa or external providers. Through their professional skills, Sherpa mentors help avoid lack of coordination and unnecessary waiting time and make sure that all available services are provided. Sherpa mentors have an assertive approach to mental health carers and social workers and thereby ensure that relevant information is distributed between services. 3) Career counselling. Professional career counsellors support participants in creating a realistic match between their competences and the demands of the job market. Participants will be given advice on how to write a curriculum vitae and job applications, on job seeking strategies, and help in practicing job interviews and negotiating employment contracts. 4) Impartial help to clarify private economy is offered by a consultancy firm, the Settlement [34], run by volunteers. The firm consists of two employees and a group of volunteers with professional backgrounds in economics, law and social counselling. 5) Contact with employers to help participants obtain jobs, and keep them. | Sherpa mentor: Newly appointed Sherpa mentors will have a 1-week introduction to working routines, and will attend a 2-day workshop introducing the IPS-MA method. Mentors with experience in the method will conduct the introduction. Team members are furthermore obliged to participate in annual refresher courses. Team members will have monthly supervision provided by a trained psychologist. | Face to face, can be via phone or email after 6 months |
| Henderson 2013 | Employment | Use of a decision aid + TAU vs TAU | To lead to improvements in gaining and retaining employment | CORAL decision aid consisted of six sessions over 12 A4 pages so was a booklet. | The decision aid included 6 sections over 12 A4 pages: a) the pros and cons of disclosure, b) personal disclosure needs, c) personal disclosure values, d) when to tell, e) whom to tell and f) making a decision. Sections b-e include service user quotes from developmental work and the final section summarises the previous sections and asks the reader to make decisions regarding whether to disclose and if so to whom, when and what to disclose. | Researchers - they met with each participant, gave them the CORAL decision aid and answered any questions. | NR |
| Herman 2011 | Housing | Critical time intervention + usual care vs usual care | to prevent recurrent homelessness and other adverse outcomes following discharge in two ways: by strengthening the individual's long-term ties to services, family, and friends; and by providing emotional and practical support during the critical time of transition. An important aspect of CTI is that post-discharge services are delivered by a worker who has established a relationship with the client before discharge. CTI shares with long-term assertive community treatment models a focus on promoting in vivo development of independent living skills and building effective support networks in the community (9). The emphasis, however, is on maintaining continuity of care during the critical period of transition while primary responsibility gradually passes to existing community supports that will remain in place after the intervention ends. Such an approach, we believe, increases the likelihood that the impact of a time-limited intervention will persist beyond its actual endpoint, which is the primary goal of CT |  | While living in the transitional residence, all participants received basic discharge planning services and access to psychiatric treatment. After discharge, participants in both conditions received a range of “usual” community-based services depending on the individual’s needs, preferences and living situation. These services usually included various types of case management and clinical treatment.  In addition to the services noted above, participants randomly assigned to the experimental condition received nine months of CTI following discharge from the transitional residence. In brief, it is a nine-month case management intervention delivered in three phases, each of which lasts approximately three months.  Phase one--transition to the community--focuses on providing intensive support and assessing the resources that exist for the transition of care to community providers. Ideally, the CTI worker will have already begun to engage the client in a working relationship before he or she moves into the community. This is important because the worker will build on this relationship to effectively support the client following discharge from the institution. The CTI worker generally makes detailed arrangements in only the handful of areas seen as most critical for community survival of that individual.  Phase two—try out-- is devoted to testing and adjusting the systems of support that were developed during phase one. By now, community providers will have assumed primary responsibility for delivering support and services, and the CTI worker can focus on assessing the degree to which this support system is functioning as planned. In this phase, the worker will intervene only when modification in the system is needed or when a crisis occurs.  Phase three—transfer of care-- focuses on completing the transfer of responsibility to community resources that will provide long-term support. One way in which CTI differs from services typically available during transitional periods is that the transfer of care process is not abrupt; instead, it represents the culmination of work occurring over the full nine months. | Delivered by 3 workers trained by several of the model developers. 2 were bachelors-level employees of the New York State Office of Mental Health reassigned to this project from their regular duties and the third worker was a more experienced worker who had delivered CTI in an earlier trial. Weekly supervision was carried out by clinically trained staff experienced in the model. | Face-to-face and individually. |
| Himle 2014 | Employment | Work-related CBT + vocational services vs vocational services only | For people with social anxiety disorder (SAD) and aims to reduce social anxiety and enable individuals to seek, obtain and retain employment | There is a manualized group CBT for SAD which was referenced as well as the JOBS program manual which was also used and referenced. | Session 1 - Psychoeducation relating to SAD and its effect on employment. Session 2 - Instruction in the identification of automatic thoughts. Session 3 - Further discussion about how SAD relates to the world of work and instructs participants in constructing rational responses to their automatic thoughts. Sessions 4-8 - Psychoeducational topic related to the world of work, in-session exposure as well as cognitive restructuring and homework exercise planning. | 3 vocational service employees were WCBT group leaders and there were two leaders for each session. 2 leaders completed approximately 50 hours and the third leader received 30 hours of training with specialists in CBT for anxiety disorders. Weekly supervisions took place. | Face-to-face and in a group. |
| Hurlburt 1996 | Housing | Section 8 rent subsidy certificate vs no section 8 rent subsidy certificate | Section 8 certificates from the U.S. Department of Housing and Urban Development (HUD) were used to implement the most important feature of supported housing, access to a range of affordable housing options. | NA | After moving into what is typically an apartment setting, certificate holders pay 30 percent of their income toward rent, and the remainder of the rental cost is subsidized by the department of housing and urban development. In the McKinney project, clients worked closely with housing specialists and case managers to apply for Section 8 certificates and to select and obtain appropriate housing. | NA | NR |
| Hurlburt 1996 | Housing | Comprehensive housing services vs traditional housing services | NR | NA | Access to vocational services. Comprehensive case managers took a formal approach to working with clients. Daily team meetings to discuss clients together so that people could fill in for each other. | Comprehensive case managers | NR |
| Kern 2018 | Employment | IPS + errorless learning vs IPS | Was based on core IPS principles of supported employment - best described as "augmented" IPS supported employment. | Errorless learning training followed previously developed manualized procedures which there is a manual for. There were instructional aids included at each step to ensure high levels of performance proficiency throughout the course of training e.g. cues, prompts, modelling, self-instruction which can all be found in supplementary material. | Participants had specific work behaviour problems targeted for intervention which were prominent areas of difficulty noted during the first 3-4 weeks on the job as measured by the WBI. Supported employment methods to address targeted WBI problems were not prescribed and left to the employment specialists discretion. Some methods include providing general support, implementation of problem-solving strategies, role-play exercises and using outside resources. After an individualized training plan was developed, a series of training steps were devised and arranged hierarchically by level of difficulty or by their natural order of sequence. The aids were used to facilitate functional independence. | Employment specialists conducted errorless learning training and implemented the intervention at the jobsite. | NR |
| Killackey 2019 | Employment | IPS vs TAU | Supported employment is acknowledged as being the superior form of vocational intervention for people with established mental illness in terms of achieving an outcome of employment in the open labour market. Supported employment is an intervention in which an individual is aided to find a job and then supported to remain in the job | IPS has eight fundamental principles, which are as follows: 1 IPS is open to any person with mental illness who wants to look for work. 2 IPS is integrated with the mental health treatment team. 3 IPS is focused on competitive employment as an outcome. 4 Personalized benefits planning/counselling is provided in IPS. 5 Job searching commences directly on entry into the IPS programme and is not determined by measures of work-readiness or illness variables. 6 The IPS worker develops relationships with employers based upon client interests. 7 Potential jobs are chosen based on consumer preference. 8 Support provided in the programme is time unlimited, continuing after employment is obtained and is adapted to individual needs. A manual for conducting IPS in FEP is now available from Orygen Youth Health (http://oyh.org.au/ online-store) | The EC’s job was to meet with clients as soon as possible after randomization and provide them with an employment service based on the eight principles of the IPS model. However, it generally entailed an assessment of vocational goals, engagement in job search, writing resumes, interview training, sourcing appropriate clothes for interviews, employment skills training, among many other tasks. As well as rapid job search and supporting the job-seeking process, the EC provided support to those who found work for the duration of the intervention. The EC also worked with participants to achieve an educational outcome such as enrolment in a course where that was the preferred or most appropriate outcome for the individual. | Employment consultant embedded within the early intervention team. background working in general and disability employment. Consultant had a decade of experience working in government-funded employment and disability employment services | Face to face. One on one |
| Kingston 2018 | Offending | Reasoning and rehabilitation2 + TAU vs TAU | Aim to help secure competitive employment for patients with offending histories. | The original programme can be found in the references. | R&R2 is a manualised group-therapy programme with the goal to reduce antisocial attitudes and behaviours, with particular emphasis placed on self-control, problem-solving skills, emotion management, conflict management, consequential thinking, alternative thinking and social perspective taking and values that underlie pro-social competence. | Facilitators are described to be the intervention providers. They attended a 3-day training session conducted by a trained clinician registered with the Cognitive Centre of Canada. | Face-to-face and group-therapy |
| Korr & Joseph 1996 | Housing | Case management vs routine care | No details given | NA | Targets persons admitted to mental hospitals and were "undomiciled," i.e., homeless at the time of admission. Staff, working as a team, bring the client from the hospital to the place where she he will live in the community. They link the client to entitlements such as Supplementary Security Income (SSI) and to mental health treatment services, especially medication. They work "in vivo," i.e., at the client's home or in the community, to teach living skills. During the first weeks of client involvement, aggressive outreach includes almost daily visits. When possible, clients are linked to rehabilitative services including supported employment. In most cases, the agency also serves as representative payee to receive the client's disability check. The agency staff see that rents are paid. Bridge staff generally placed the homeless clients in this project in single room occupancy (SRO) hotels or apartments on the north side of Chicago. These were the types of places that are affordable for clients on SSI. To pay the first month's rent for clients who were not yet receiving SSI, Bridge staff could obtain a loan from a revolving fund established by the Illinois Department of Mental Health and Developmental Disabilities. | The team for this project consisted of five case managers. The maximum number of clients at any time was about 5Wen clients per case manager. Some of the case managers, the team leader, and the site director were African American | Face to face individual case management |
| Kukla 2018 | Employment | CBT + cognitive remediation vs vocational support | Based on CBT intervention IVIP = The Indianapolis Vocational Intervention Program (IVIP) which is work-focused CBT designed for people with schizophrenia spectrum disorders engaged in non-competitive work. The goal of IVIP is to assist people to learn to identify cognitive processes and correct work-related dysfunctional beliefs and behaviours. | To do the cognitive remediation tasks, a computer is needed. | IVIP - Is a manualised curriculum with the following topics: thinking about work, barriers to work, workplace relationships and realistic self-appraisal. The group sessions included a structured agenda, instruction on the basic CBT principles applied to work, job-related feedback and peer support. Weekly hour long IVIP individual sessions provide further opportunities for examination of work-related thoughts and behaviour using the CBT principles. Cognitive remediation - Participants perform exercises using Posit Science Brain Fitness and Insight Software developed using neuroplasticity models focusing auditory and visual cognitive domains. They focus on sensory discrimination and advancing to higher level cognitive abilities, including working memory, set shifting and problem-solving. | Cognitive remediation - Labs were supervised by research assistants who provided one-to-one software orientation and monitoring as needed and their role was to ensure participants were actively training. IVIP - IVIP therapists were experienced masters level clinicians and were given initial training facilitated by the senior author, a clinical psychologist. | Face-to-face/internet/individual/group |
| Lamberti 2017 | Offending | Forensic ACT vs Enhanced TAU | Based on a conceptual framework that uses legal leverage to engage justice-involved individuals in treatments and services that target criminogenic risk factors. | NR | The FACT group participants received legal leverage in the form of judicial monitoring within a standard criminal court setting and all were enrolled in a single treatment team. FACT group clinicians conducted intake assessments that included review of available criminal justice and health records to identify risk factors for criminal recidivisms and they incorporated the risk factors into service plans for each participant. | FACT clinicians. In preparation for study initiation, two authors provided approximately 6 hours of instruction in mental health-criminal justice collaboration to all group clinicians, the judge who monitored the FACT participants and the attorneys for the FACT participants. The FACTS was utilised as part of training to provide an overview of the Rochester FACT model. | Face-to-face and individually. |
| Lecomte 2019 | Employment | CBT for supported employment vs supported employment | To improve work outcomes of obtaining a competitive job, number of the weeks worked and the number of hours worked per week. | There was a CBT-SE manual which contained information on the sessions and can be found in Table 2 of the paper. | CBT-SE works on job acquisition earlier on and focuses more on negotiating accommodations and coping with stigma at work. Each session involves review of the homework, presentation of the theme of the session, presentation of some didactic information, discussions and application to self of the material, writing relevant information, review of the session and presentation of the homework. The last session ends with a small graduation celebration involving a certificate for each person's participation and a group meal. Participants had a manual on all 8 sessions as well as homework they had to complete outside of the session. The manual had the following: Session 1 - Coping with stress at work. Sessions 2-3 - Recognising and modifying my dysfunctional beliefs linked to work. Session 4 - Overcoming obstacles linked to reintegrating the workplace. Session 5 - My strengths and competencies related to work. Session 6 - Accepting criticism and asserting myself appropriately. Session 7-8 - My best coping strategies for work. | Two co-therapists led each session. The therapists were trained by the team and hired for the study. Therapists training took place over the course of two days by one of the investigators and co-developers of the manual. | Average of 5 participants per group. |
| Lehman 1997 | Housing | ACT vs usual community services | Focus on engagement with client, stabilization and developing skills to maintain a stable lifestyle in the community, and maintenance and ongoing treatment | NA | Each patient was assigned to a "mini-team" consisting of a clinical case manager (case load, 10-12 patients), an attending psychiatrist, and a consumer advocate. The entire ACT team, including the consumer advocates, worked together in decision making and each staff member was knowledgeable about most of the patients. Short-term housing interventions include: motels, crisis bed, transitional shelter. Long-term housing interventions include: section 8 housing subsidies. independent apartments and rooms, board-and-care providers. | continuity of care, and the team was available 24 hours every day. The ACT team consisted of 12 full-time equivalent staff, including a program director with a masters' degree in social work, a full-time psychiatrist and medical director, 6 clinical case managers (social workers, psychiatric nurses, and rehabilitation counsellors), 2 consumer advocates, a secretary-receptionist, a part-time family outreach worker from the Alliance for the Mentally 111 of metropolitan Baltimore, and a part-time nurse practitioner to treat chronic medical problems | Face-to-face, very little details |
| Lindenmayer 2008 | Employment | Cognitive remediation vs computerized control | Providing therapeutic intervention to alleviate any cognitive difficulties experienced by people diagnosed with "severe and persistent" mental health conditions may improve social and vocational functioning, since cognitive difficulties are linked to poorer outcomes in these areas. | Participants conducted cognitive exercises on a computer. Exercises were part of the COGPAK version 6.0 package standardized curriculum and targeted "attention and concentration, psychomotor speed, learning and memory, and executive functions". COGPAK reference: COGPACK, Version 5.1. Ladenburg, Germany, Marker Software. Available at www.cogpack.com/usa/frames.htm | Cognitive remediation therapy: Participants engaged in twice weekly sessions of computer practice, completing the computer exercises that targeted each specified cognitive function. Feedback was provided to participants about their performance. Participants also took part in a once weekly discussion group that focused on developing "compensatory strategies" for persistent cognitive difficulties, “the importance of cognitive skills" and engaging in activities of daily living and work. After completing the intervention, participants in both study groups were offered employment in a work program, that provided salaried, contracted jobs within the hospital (such as cleaning and kitchen assistant roles), following completion of a successful interview. | Cognitive remediation (including the computer training and discussion group) was supervised by three hospital staff members: two psychologists and a psychology/occupational therapy intern. The provider delivering the training is not stated. | The cognitive exercises were delivered via a computer. Discussion groups were provided face-to-face |
| Lipton 1988 | Housing | Residential treatment vs standard care | No details given | NA | St. Francis Residence II supportive housing. Through its linkages with city, state, and voluntary agencies, the residence provides an integrated and comprehensive array of services to chronic mentally ill patients who are homeless or at risk of becoming homeless. In addition to a furnished room, the program offers individualized case management, coordination of public assistance or Social Security benefits, medication monitoring, money management, meals, activity therapy, and, when appropriate, referrals to psychosocial and rehabilitation programs. Through a collaborative | Psychiatric treatment through Bellevue Hospital. Housing and case management from the St Francis Residence | NR |
| Lloyd-evans 2020 | Social Isolation | Community navigator programme + routine care vs Routine care | A socially-focused programme aiming to reduce loneliness for people with complex depression or anxiety. | The CONSORT checklist of the study is available in the supporting information section as a word document. The programme manual is available on the study website (URL given in reference) and refers that the development and theoretical basis are described more fully in published protocol paper (provided as a reference). | Had three main components. 1) Community Navigator helped participants use a social network mapping tool to map out what is important to the participant and potential areas for new social activities or strengthening existing connections.  2) Community navigator helped participants develop a "connections plan" which identified goals to increase connectedness and social relationships and the community navigator offered practical help or support in achieving these.  3) Community Navigator organised three group meet-up sessions with all participants receiving the program to allow people to meet, initiate friendships and share experiences of programme. | Intervention providers were not required to have mental health professional training or qualifications, but were recruited on basis of excellent interpersonal skills, awareness through personal or work experience of the challenges faced by people with serious mental illness, excellent knowledge of their local community and some previous work experience of supporting social inclusion and helping people develop social connections. Community Navigators were given 5 days training delivered by members of study co-production group and training involved: familiarisation and practice using mapping tool, guidance in using a solution-focused approach to support participants, focusing on strengths and what participants can do to develop connections, and information about nature of patient group. A one-day induction in each clinical service was also provided and three days "top-up training" including a one-day workshop on coaching and peer-learning skills. Fortnightly group supervision was provided to the Community Navigators by experienced social work and occupational therapy practitioners. | Face-to-face and mainly individually, but with up to three group sessions over six-month period. |
| Lysaker 2005 | Employment | Vocational CBT program vs TAU | Beliefs that impact negatively on work functioning and views of the self may deleteriously affect employment experiences for people diagnosed with schizophrenia spectrum disorders e.g. expectation of failure may cause many to leave a job. Providing therapeutic support to change the way people think about themselves in an employment context may improve vocational outcomes. The intervention uses techniques and principles of cognitive behavioural therapy (CBT), due to the effectiveness of this approach for changing cognitions. | IVIP is a manualised intervention. The article reports that the treatment manual is available form the authors on request. | Both control and intervention group participants engaged in work placements at the medical centre where the study took place throughout the study. The IVIP included a CBT group and individual sessions: The once weekly CBT group had a structured curriculum covering four topics: "1. Thinking at work 2. Barriers to work 3. Workplace relationships 4. Realistic Self-appraisal". There are three core group activities: 1. Teaching the module content 2. Supporting participants with an exercise that practices the module teaching 3. Offering participants feedback on the exercise to support their progress. One-to-one CBT sessions were provided to participants to enable personal application of module material of individual beliefs interfering with work and self-view. | A therapist provided the individual and group CBT sessions (no further details reported) | Group and individual sessions were delivered fact to face |
| Marder 1996 | Social Isolation | Social skills training vs supportive group therapy | The social skills training procedures were designed to compensate for the symptoms and cognitive deficits that are associated with schizophrenia. The group leader used cognitive restructuring principles, repeated behavioural rehearsal, video modelling, and abundant positive social reinforcement to overcome the intrusion of symptoms, distractibility, and lack of motivation that some patients demonstrated. | NA | Subjects participated in modules on medication self-management and symptom self-management during the first 6 months of the trial. As the names denote, these modules comprise skill areas with educational objectives for recognizing symptoms and side effects, self-administration and monitoring of medication, negotiating medication issues with doctors and other providers, avoiding street drugs and alcohol, and identifying warning signs of relapse. A social problem- solving module was completed during the second 6 months. This module was designed to enhance the ability to recognize social barriers to attaining community life goals and to generate, select, and implement appropriate solutions. Finally, a successful living skills module was completed by subjects who continued into the final year of the study. This module enabled subjects to identify and pursue individualized and personal goals by using the basic model of social skills training.  Each of the modules has a similar structure. The modules begin with an introduction to self-management (which highlights the rationale for the training and the goals and benefits of the training and enhances motivation to participate), move to a training segment (in which the substantive knowledge and skills are detailed), next move to phases that teach problem-solving skills in the areas of resource management and overcoming outcome problems, and end with in vivo exercises and homework assignments to practice the acquired skills learned in natural environments. The training is continued for each patient until criteria for mastery of the knowledge and skills are met. | doctoral or masters level psychologists, an occupational therapist and a social science technician | NR |
| McGurk 2007 | Employment | Supported employment + cognitive training vs supported employment | there is a growing literature indicating that cognitive rehabilitation can produce modest improvements in cognitive functioning, although there continues to be a debate as to whether these improvements translate into functional gains in domains such as social functioning, self-care, and work. These findings suggest that systematic efforts at improving cognitive functioning are feasible, although special consideration may be required in order to ensure that these gains are transferred to employment settings. | Cogpack, version 6.0, Marker Software was used to deliver the computer cognitive training | Assessment-thorough cognitive assessment Computer cognitive training- around 24 hours computerised cognitive training over 12 weeks job search planning-cognitive training specialist and employment specialist meet together with the client to plan the job search based on the clients vocational preferences and evaluation on cognitive gains and consider possible needs and supports for employment Job support consultation-following job attainment, the cognitive training and employment specialist meet regularly with or without the client and discuss job supports to address cognitive challenges in the job, these are designed to help the transfer of cognitive skills learnt during computer training. | Cognitive training specialist: (training not mentioned) Employment specialist: (training not mentioned) | Face to face/computer-based training, individual |
| Mcgurk 2015 | Employment | Enhanced supported employment + cognitive remediation (thinking skills for work) vs enhanced supported employment only | Aims to enhance cognitive performance at work, and therefore improve work functioning and outcomes | Cognitive specialist uses a 24-session standardized curriculum of exercises derived from the COGPACK training program, version 7.0. Use of computer for the cognitive exercises | The Thinking Skills for Work program uses three approaches to enhance cognitive performance at work: cognitive exercise practice, strategy coaching, and teaching coping/compensatory strategies. Participants received a packet of the cognitive exercise curriculum, which was used to guide exercise completion at an individual pace, to record newly learned strategies and performance scores, and to monitor performance progress. The cognitive specialist supplemented task practice with strategy coaching to improve efficiency of task approach and performance, such as teaching how to chunk information to optimize retention. Broader targets for coaching included work-relevant behaviours observed in the sessions, such as timeliness, focus on the curriculum, and appropriate dress and demeanour. Based on the assessment, participants were also taught coping strategies to reduce the effects of cognitive impairments on vocational functioning (24). Specific strategies were identified from input from the client and the employment specialist; they were initially taught by the cognitive specialist and were followed up by the employment specialist. | Cognitive specialist | Face-to-face |
| McGurk 2016 | Employment | Enhanced vocational services + cognitive remediation (thinking skills for work) vs enhanced vocational services only | Multi-component, manualized, cognitive remediation program | Participants received a packet of the cognitive exercise curriculum which was used to guide exercise completion at the person's individual pace, for recording newly learned strategies and performance scores, and for monitoring progress in performance. | Once referred to the program, participants were first assessed and this led to identification of cognitive enhancement strategies. Participants were engaged in computer-based cognitive exercise which provided practice across the broad range of cognitive functions e.g. attention and concentration, psychomotor speed, learning and memory, and executive function. Exercises targeting these areas of cognitive functioning were included in the first 6 sessions with additional sessions focusing on further practice in these areas. The software provided performance scores reflecting accuracy and speed following the completion of each exercise. The computer exercises were designed to be enjoyable and reinforcing with difficulty gradually increasing over time. Based on the assessment, participants were also taught self-management strategies for improving cognitive performance and reducing the effects of cognitive impairments on achieving job goals. The cognitive specialist, the employment specialist and the participant met together to plan the job search, based on the individual's vocational preferences and a meeting was held to review their job interests. The team then worked together as needed on a consultation basis. | Cognitive specialists - Training for the TSW program included a workshop, didactics, role modelling, demonstrations and observation and feedback on cognition functioning in psychiatric illness, cognitive remediation methods for psychiatric illness more broadly and specifically as applied in the TSW program and integration with vocational services. | Digital intervention via computer software and provided individually |
| McHugo 2004 | Housing | Integrated housing vs Parallel housing | NR | NR | The two programs differed primarily in their approach to housing. Although each program was a hybrid, the parallel housing services program more closely resembled a traditional supported housing approach, whereas the integrated housing services program included aspects of the traditional continuum model. Based on idealized implementation of the underlying models, the two housing programs should differ on four dimensions: 1. some of the housing is owned or leased by the mental health provider. 2. Continuum housing units are in apartment buildings in which all, or a majority, of the units are occupied by mental health consumers. 3. the mental health provider often links housing with treatment participation. 4. some of the congregate housing units contain live-in staff. | Multidisciplinary mental health teams | Face to face, Individual |
| Mervis 2017 | Employment | Indianapolis vocational rehabilitation programme vs supportive therapy | Reduce defeatist beliefs related to work functioning, based on CBT principles. IVIP is a manualised treatment that draws from elements of CBT models with the primary goal to assist individuals suffering from serious mental illness so that they can return to work. The therapy targets defeatist beliefs that are maladaptive and automatic through cognitive techniques, psychoeducation, handouts, and semi-scripted role-playing | NR | There are four therapeutic models, each of which contains two sessions and covers issues relevant to work environments. 1) Identifying and changing thoughts about work 2) Identifying problems and generating strategies for prevention and solutions 3) Understanding and interacting with others in the workplace 4) Identifying, understanding and optimising personal strengths and weaknesses in the context of work. | Graduate level therapists (one masters level and one post-baccalaureate) were trained on IVIP by author and all therapists had weekly supervision. | IVIP was offered in small groups with 2-3 participants, a group leader and co-therapist. |
| Milligan-Saville 2017 | Employment | RESPECT manager mental health awareness training vs waitlist | Many managers feel reluctant or under-skilled to contact an employee who is on sick leave or showing signs of ill health, especially if the illness concerned is a mental disorder, as they might fear contact could cause harm or lead to complaints. The aim was to promote manager understanding of mental health problems among workers. | NA | Training consisted of three main topics: key features and effects of common mental health issues in the workplace; roles and responsibilities of senior officers in terms of employee mental health; and development of effective skills for discussing mental health matters with staff. The first phase of training focused on the symptoms of depression, anxiety, post-traumatic stress, and alcohol misuse, and how these conditions can be recognised in the workplace. During the second phase of training, helpful responses towards a subordinate with an identified mental health problem were contrasted with poor management practices. Positive communication techniques were then implemented in group discussions. An important component of the third phase of training was learning how to implement the RESPECT principles outlined below when contacting a worker who might be suffering from mental health problems. The RESPECT principles are as follows: Regular contact is essential; the Earlier the better; Supportive and empathetic communication; Practical help, not psychotherapy; Encourage help-seeking; Consider return to work options; Tell them the door is always open and arrange next contact. | Clinical psychologists or consultant psychiatrists | Face-to-face + one phone call. |
| Morse 1992 | Housing | Continuous treatment team vs outpatient mental health services | No details given | NA | Specific service principles included a “no-reject” policy, provision of community-based services for an unlimited time, and a flexible, individualized approach to address clients’ multiple needs. The model was adapted to meet the special problems associated with homelessness by conducting outreach to shelters to engage reluctant and suspicious clients and prioritizing client needs for basic survival (food, shelter, and clothing) and permanent housing. In addition to outreach, service activities were targeted to three areas-individual change, environmental change, and support for bridging the gap between clients’ needs and environmental. Activities encouraging individual change included helping clients to form an ongoing therapeutic relationship and to learn better ways to cope with problems, linking clients with psychiatric medication services, teaching community living skills and interpersonal skills, and providing crisis intervention. Environmental change was promoted through casework advocacy to obtain resources from agencies addressing clients’ welfare, housing, and health needs. Staff also intervened with persons in the clients’ environment, such as landlords or shelter providers, to encourage more positive reactions to clients. | Clinical case managers within the treatment team | Face to face, individually |
| Morse 1997 | Housing | Broker case management vs ACT | No details given | NA | In the broker case management condition, the case manager’s role was to develop an individualized service plan for the client, arrange for and purchase mental health and psychosocial services from various service providers, monitor the quality of purchased services, and adjust the mix of services based on the client’s changing needs. These case managers were much more office based than case managers on the assertive community treatment teams. Unlike their team counterparts, they rarely went into emergency shelters, made home visits, or accompanied their clients to other agencies and potential housing sites. | Case manager | Face to face individual case management |
| Morse 1997 | Housing | ACT with community workers vs ACT | No details given | NA | Treatment principles were similar to those of other assertive community treatment programs (and included intensive individualized treatment, responsibility for providing or coordinating all services needed by the client, persistent follow-up, and in vivo service delivery. Assertive community treatment staff were instructed to frequent shelters and were trained in homeless outreach and engagement methods. Outreach and engagement strategies included focusing on developing a positive relationship with the homeless person and assisting him or her with basic needs such as for food, shelter, and transportation. Emphasis was also placed on developing service plans that followed the priorities stated by each client, which often involved assistance in obtaining housing and entitlements before traditional mental health treatment. The staff also prioritized service activities that would help clients obtain housing and entitlements. Other activities included supporting landlords in solving clients housing problems, tracking clients’ Social Security and housing applications, and advocating on behalf of clients with staff from other agencies for access to benefits. Community workers: Clients were also assigned a paraprofessional community worker whose role was to assist with activities of daily living and to be available for leisure activities. Typically, the community worker spent more time with the client in the latter phases of treatment, after initial stabilization. | The assertive community treatment team consisted of five to seven persons, with backgrounds primarily in psychology, social work and counselling. + a paraprofessional community worker | Face to face individual case management |
| Morse 2006 | Housing | Integrated assertive community treatment vs standard care | An approach to treat problems for people with dual disorders. The essential feature of integrated treatment is that the same clinician (or team of clinicians) provides both mental health and substance abuse treatment in a co-ordinated manner. | NR | Integrated treatment tends to emphasize a) assertive outreach b) motivational interventions c) a stages-of-treatment approach d) cognitive behavioural counselling e) interventions to strengthen social networks supportive of recovery f) a long-term perspective | A new IACT clinical team was created, although many of the staff had prior experience working with homeless people who had SMI. The team received training and follow-up consultation from project personnel regarding ACT treatment principles and practices. Research personnel and national experts provided the IACT team with training and consultation on integrated treatment principles and services. The IACT had a substance abuse specialist on staff and provided substance abuse services directly. | NR |
| Mueser 2005 | Employment | Skills training programme vs TAU | Although supported employment programs are effective for improving vocational outcomes for people experiencing severe mental health conditions, some difficulties persist including short duration of employment. This may be due to social difficulties encountered at work e.g. in interactions with colleagues, clients and supervisors. Therefore, social skills training may promote positive social interactions and help improve work experiences and outcomes for people accessing supported employment programs. | Materials used to provide training: 1) Trainer's manual: specifies what should be done and said by provider of the intervention; 2) Videotape: used to demonstrate how to solve problems at work; Participant materials: Participant Workbook. Reference for intervention development provided | All participants were employed in a supported employment program. The intervention addresses nine different skill areas: 1) "how work changes your life" 2) learning about your workplace 3) Identifying stressors at work 4) "problem solving" 5) "managing mental health" 6) "managing physical health" 7) "improving job performance" 8) "making friend and socialising" 9) "using supports and staying motivated". These skills are taught weekly in group sessions, with monthly booster sessions available after to maintain learning and individual sessions to catch up were offered to people who missed sessions. | NR | Face-to-face group sessions with 3-5 clients; individual catch-up sessions provided to participants who missed group sessions |
| Noordik 2013 | Employment | Exposure based return to work intervention vs TAU | Therefore, we developed an exposure-based RTW (RTW-E) intervention to be integrated into usual care. With this intervention, we aimed to stimulate workers to use active problem-solving behaviour and prevent avoidance behaviour when dealing with stressful work situations during RTW. | The homework assignment forms and the worker information brochure can be downloaded from http://www.psychischenwerk.nl/datafiles/patientinformationbrochure_RTW-E_english_okt%202006(l).pdf and http://www.psychischenwerk.nl/datafiles/FIomework_RTW-E_A-F_%20english_%20febr%202007( 1 ).pdf. | In the RTW-E program, workers received CAU and were gradually exposed in vivo to more demanding work situations structured by a hierarchy of tasks evoking increasing levels of anxiety, stress, or anger.   CAU aims to help workers regain control and rebuild social and occupational contacts and activities, according to the guidelines for CMD (19, 26). The OP can reach this goal by using recommended methods such as stress inoculation training, cognitive restructuring, graded activity, and time contingency during the RTW. | Occupational physicians recruited from various occupational health services located throughout the Netherlands. items). The OP in the RTW-E group received two days of training in the RTW-E program. | Individual, face-to-face and homework |
| Nuechterlein 2019 | Employment | IPS + Workplace fundamentals module vs brokered vocational rehabilitation + social skills training | Maximise the impact on work recovery after a first psychotic episode | The WFM materials could be found online: https://www.psychrehab.com/modules/module_workplace.html | IPS followed the principles of supported employment but was adapted to a first-episode sample by providing supported education for participants whose preferences and situations made resuming education more appropriate than returning to employment. The common principles are: 1) goal is competitive employment or schooling in community settings 2) IPS services are integrated into the mental health treatment team 3) rapid search for schooling or employment 4) eligibility based on participant choice 5) attention to the individual's job/school preferences 6) continuous individualised support 7) community outreach and 8) disability benefits counselling. The WFM uses a group-based skills training approach emphasising social and problem-solving skills necessary for keeping a job with skills area like how work/school changes your life, learning about your place of work/school, identifying stressors, solving problems, managing symptoms and medications, managing health, improving school/job performance, socialising with fellow workers/students and finding motivation. Each of the skill areas included showing videotaped scenarios, role played practice, generation and evaluation of solutions to individually relevant school/work problems and individualised homework assignments. | The IPS specialist was a member of the clinical team of psychiatrists, psychologists, and social workers, coordinated treatment with them to optimize work recovery. | Face-to-face and individually for IPS and face-to-face but group for WFM. |
| Okpaku 1997 | Employment | Employment-orientated case management vs TAU | The primary purpose of this intervention was to minimize the clinical and administrative obstacles to employment for these individuals. The model combined the direct services of specially trained psychiatric vocational rehabilitation specialists with the supervisory and consultation roles of an interdisciplinary team from various agencies, as opposed to a single agency. In this way, the administrative leverage of the team could solve interagency coordination problems | NA | A specialist reviewed each client’s history, gave an assessment of the client’s needs, and presented a treatment plan designed to return the client to an appropriate level of work. The initial treatment plan and the team’s revisions were completed without knowledge of the individual’s assignment.  For individuals in the E group, the vocational specialists prescribed the revised treatment plan in consultation with the client and the client’s existing case manager or therapist. The specialists aggressively pursued social and rehabilitative services to enable the clients to be appropriately placed in a work setting. | Vocational specialists supervised by a team that consisted of the psychiatrist and vocational counsellors and therapists primarily from the state hospital and a private psychosocial rehabilitation facility. | Face to face - individual |
| Overland 2018 | Employment | work directed CBT and job support programme (At work and Coping) vs TAU | NR | NR | The CBT module focused on managing mental health problems as they related to work situations and addressing work situation and participation as an integrated component in the therapeutic process. The IPS adhered to the principles in the IPS model developed for people with SMI but didn't follow the strict protocol or running fidelity measures on adherence. Individual job support was offered to those in need of individual job support to facilitate workplace adaptions or identification of appropriate employment. Within AWaC, integration referred to a combination of therapy and an explicit work focus on one process to avoid parallel lines of action. | Mini-teams of therapists and employment specialists. | NR |
| Pos 2019 | Social Isolation | CBT for social activation vs TAU | The overall aim of the treatment was to modify dysfunctional beliefs and to increase engagement in constructive social activity in individuals with prominent negative symptoms - the primary focus was to help participants become more active in reaching personal social goals. | A treatment manual was used in the individual therapy sessions for psychoeducation and interventions that could be used when needed. Cognitive restructuring worksheets were used as part of the group sessions. | i) The first session of group therapy was psychoeducation with a focus on negative symptoms and participants were asked to share their experiences and define their individual social goals. To promote peer support, buddy couples were formed to encourage support between sessions in completing homework.  ii) At the start of the second session, goals with the highest personal value were selected and steps to reach these goals were discussed. Participants were asked to rate which obstacles in reaching their goals they anticipated and emphasis was placed on dysfunctional beliefs regarding one's own cognitive functioning, skills or the expected pleasure or social devaluation. Psychoeducation focused specifically in these sessions on the cognitive model of negative symptoms and the role of dysfunctional beliefs, avoidance behaviour and demoralization.  iii) Subsequent sessions focused on challenging anticipated and experienced obstacles using standardised materials such as cognitive restructuring worksheets, role-plays and on-site behavioural experiments.   Specific actions to reach goals were discussed in detail during the sessions with the participant carrying out the step before the next planned session as homework. Individual sessions - These sessions aimed continuation of social activation and achievement of personal goals through a personalised focus on the person's main dysfunctional beliefs and associated behaviours and countering obstacles to these goals. A treatment manual provided material for psychoeducation and interventions that could be used. Intervention tools were based on different elements of demoralization e.g. cognitive techniques (socratic dialogue), cognitive imagery techniques and behavioural techniques. | Four psychologists with a master's degree in clinical psychology and four master students with a bachelor’s degree in clinical psychology carried out assessments. They received extensive training prior to inclusion by certified trainers for the PANNS, BNSS and GAF. The training was three-training videos for the PANSS, provided by AMC as part of their clinical training program and three training videos for BNSS provided by the original authors. Prior to assessment, all rates participated in a consensus training and fulfilled the criteria of interrater reliability >0.8. Ongoing weekly supervision was provided to ensure consensus across raters. | Face-to-face and both provided individually and in a group. |
| Pot-Kolder 2018 | Social Isolation | Virtual reality CBT vs TAU | Aiming to lead to more time spent with other people and a decrease in momentary paranoia, perceived social threat and anxiety during real-life social situations. | A lot of digital equipment was required as the four virtual social environments were created using a Vizard software (which was found in the appendix). Participants would need a Logitech F310 Gamepad and a Sony HMZ-T1/T2/T3 Head Mounted Display with a high-definition resolution of 1280x720 per eye, with 51.6 diagonal field of view and a 3DOF tracker for head rotation. The VR-CBT manual described a structured treatment plan for all 16 sessions and was used to train the VR-CBT therapists. | An individualised care formulation guided exposure to idiosyncratic social environmental cues that elicited fear, paranoid thoughts and safety behaviours. There are four virtual environments e.g. a street, bus, café and supermarket and the patient with the therapist communicate during the virtual-reality sessions to explore and challenge suspicious thoughts during social situations, drop safety behaviours during social situations and test harm expectancies. No homework exercises were given between sessions to test the effects of the in-virtuo exposure without the effects of structured in-vivo exposure. Therapists could vary the number of human avatars, the characteristics of the avatars and the avatars responses to the patient to match the paranoid fears of the patient. | VR-CBT therapists were psychologists with at least basic CBT training who received 2 days training in VR-CBT. The VR-CBT manual described a structured treatment plan for all 16 sessions. Therapists were supervised in a group for 4 hours every month. | Virtual and individual. |
| Priebe 2020 | Social isolation | Matched with a volunteer partner who had no history of psychiatric disabilities vs not matched with a volunteer partner | Reduce the frequent social isolation of patients with schizophrenia and thus improve health outcomes. The intervention was developed through mapping the policies and practices of existing programmes and through discussions with experts. | During training, volunteer-participants were given resources for supervision and support. In the intervention, an activity booklet was used. | Patient-participants allocated to the intervention were contacted after randomisation to arrange an initial meeting with the volunteer coordinator to establish their interests and preferences for a volunteer. The volunteer coordinator arranged and facilitated an initial 'matching' meeting with a volunteer selected on their basis of preferences and availability. During the meeting, both parties were given an activity booklet which had options for free or inexpensive activities in the local area. Following the meeting, the volunteer and the patient were asked to meet weekly for a year and encouraged to engage in joint activities. Patients and volunteers were invited to inform the volunteer coordinator within the first month if they wanted to be matched with someone different. Monthly social events included food/an activity and were organised by the programme to provide opportunities for different volunteers and patients to meet and interact. Volunteers reported the occurrence, length and content of meetings via text or a phone call to the coordinator. Both parties were regularly reminded of weekly meetings and could request supervision to problem-solve any emerging challenges in the relationship. | Volunteer-participants were given initial training for 2 full days, covering general information about the programme, symptoms of schizophrenia, responsibilities and boundaries in befriending and resources for supervision and support. The volunteer-participants were recruited from various sources including flyers in local community centres and universities. Eligible volunteers had to be 18 years or older and have sufficient command of English and was checked through an application form, interviews and a criminal record check. Exclusion criteria were the receipt of treatment from secondary mental health services, any unspent criminal conviction and a current professional role in mental health services. | Face-to-face and individually. |
| Rebergen 2009 | Employment | Guideline based care (an activating approach, time contingent process evaluation, and cognitive behavioural principles) vs TAU | Based on research that Ops having a more active role in patient return to work may make the process faster | The OPs were encouraged to use specific tools, such as symptom questionnaires, patient information leaflets on stress, and day structuring exercises. | The guideline is based on an activating approach, time contingent process evaluation, and cognitive behavioural principles. Cognitive behavioural principles include stress inoculation training and graded activity to enhance patient problem solving capacity. Proposal of work-related interventions such as gradual return to work, regular supervision and work accommodations. | Occupation physician: OPs participating in the study received a 3-day training course by experienced OPs and psychologists in delivering GBC. | face to face, individual |
| Reme 2019 | Employment | IPS vs TAU | Aims to increase the rate of employment in patients with moderate-to-severe mental illness | NA | Individual placement and support (IPS) is a model of vocational rehabilitation that aims to help people with severe mental illness obtain and maintain competitive employment in ordinary jobs. IPS follows a strict manual to secure integration of supported employment with psychological treatment. The following eight principles are incorporated in the approach to vocational rehabilitation:  (i) eligibility based on the patient’s own choice (ii) focus on competitive employment (iii) integration between mental health and employment services (iv) job support guided by the patient's preferences (v) financial counselling about social security benefits (vi) rapid job search initiation (vii) employment specialists’ engagement in systematic job development individualized and continuous job support | Does not say | Individualised |
| Rivera 2007 | Social Isolation | Peer-assisted case management vs Standard case management | Inclusion of mental health consumers has been associated with reduced hospitalisation and greater client satisfaction, and better social functioning. Having consumer case managers could lead to more face to face interactions. Peers could provide support that offers general friendship, reduces stigma, and builds hope. | NR | Peers engaged clients in social activities and developed supportive social networks among clients. They planned one on one and group social activities. Peers also contributed to treatment planning and provided valuable information during weekly team meetings. | Standard care provided by licensed clinical social workers who were supervised by program directors with extensive experience in providing psychiatric rehabilitation services. Staff received 40 training hours and competency testing before working with clients. Staff also received one hour of individual supervision, one hour of group supervision, and 1.5 hours of training weekly.  Consumer providers (peers): History of multiple hospitalizations. Peers participated in the same orientation and training as professional staff, with modifications to address their specific roles. They were supervised by the full-time social worker who met with them individually and in groups to solve problems with the engagement of clients to plan activities. | Face to face. Group and individual activities |
| Roberts 2014 | Social Isolation | Social cognition and interaction training vs TAU | seeks to improve social functioning by targeting the mental operations underlying social interaction, known as social cognition. This approach is promising because social cognition predicts social functioning in schizophrenia even more strongly than do traditional neurocognitive domains. | NA | SCIT uses a combination of psychoeducation, drill-and-repeat skill practice, strategy games, heuristic rehearsal, and homework assignments to remediate deficits and decrease biases in social cognition. Each SCIT group participant was encouraged to identify a ‘practice partner’, a family member or acquaintance who was willing to practice SCIT skills with the participant weekly in lieu of, or in addition to, traditional homework. All SCIT group members identified practice partners, and partners were provided with a set of handouts and phone check-ins to guide their participation. SCIT clinicians attempted to reach practice partners by phone each week to check-in and provide guidance in their efforts to support SCIT participants’ learning. | Two clinicians in each group - advanced doctoral students in clinical psychology trained in SCIT. | Group format, face-to-face |
| Rodriguez Pulido 2019 | Employment | IPS plus cognitive remediation vs IPS | One of the variables that hinder the social functioning of people with schizophrenia is the neurocognitive deficit. Aim is to determine whether a supported employment programme along with cognitive rehabilitation could improve employability and job outcomes in people with severe mental illness with more than two years in mental health units and previous job failure. Hypothesize that the IPS strategy combined with cognitive rehabilitation will improve cognition (verbal learning, memory, attention, psychomotor speed and executive performance) and the results of competitive work. That improvement could be attributed to the immediate opportunity to implement in real contexts the learning obtained and in the integrated follow-up of the employment teams with the community mental health teams. | The Cogpack web program was used for the cognitive rehabilitation element of the intervention. | The Cogpack tasks were selected individually based on the results obtained with the measurement of the battery of reference tests plus the results of the first 6 sessions that were taken as a general practice of cognitive exercises. If the participant in the rehabilitation group got a job, they would continue to receive cognitive intervention adapting it outside of the assigned work schedule. The employment specialists kept track of the entire search process and met with the participants to plan the job search and if they had got a job, the employment specialist would follow the evolution that he had within the company. Support and follow-up was made with the participant, his family and interlocutor in the company and if there was any work problem, the interlocutor in the company communicated it and the vocational team intervened. | There were 5 IPS team technicians provided this service to clients. | Face-to-face/phone/internet - individually. |
| Rogers 2006 | Employment | Psychiatric vocational rehabilitation vs enhanced state vocational rehabilitation | Outlines how practitioners diagnose, plan and intervene to help individuals with psychiatric disabilities develop the skills and supports required to be successful and satisfied in their employment environment | NA | The experimental intervention was based on the CGK process of psychiatric vocational rehabilitation, which outlines how practitioners diagnose, plan, and intervene to help individuals with psychiatric disabilities develop the skills and supports required to be successful and satisfied in their employment environment (Anthony, 1979; Anthony et al., 1990; Anthony et al., 2002). The diagnostic, planning, and intervention components used in this approach, named PVR for this study, were developed to clearly define practitioner skills as well as the knowledge to use the skills most effectively. These included assessing rehabilitation readiness, developing rehabilitation readiness, setting an overall rehabilitation goal, functional assessment, resource assessment, planning for resource development, direct skills teaching, skills programming, resource co-ordination, & resource modification. | trainer in PVH who was considered the "gold standard" expert in the intervention provided extensive guidance and supervision to the project trainers as a way of ensuring fidelity to the intervention | 3 times per week in the classroom instruction in career planning and individual meetings with a vocational specialist for job placement or further education and training |
| Rossler 2020 | Employment | IPS with 55h placement budget vs IPS with 25h placement budget | Successful employment appears to be more likely during the first few months, suggesting that lower placement budgets may lead to faster placement into competitive employment. | NR | In accordance with the IPS model, the job coaches assisted the participants in two ways: first by assisting with placement (engagement, assessment and finding a job that matched a client’s skills and interests), and second by providing support (help in maintaining competitive employment). The time restriction applied to assistance with the job search only. Independently of their assignment to different placement budgets, all participants received unlimited support with job maintenance once they successfully started competitive employment. | Job coaches trained in the IPS model by experienced job coaches. They had weekly meetings with supervision. | NR |
| Rowe 2007 | Offending | Group/peer support vs standard services | Based on a "theoretical framework of citizenship" - the strength of people’s connections to the rights, responsibilities, roles, and resources available to people through public and social institutions and through the informal, “associational” life of neighbourhoods and local communities. We used the citizenship framework with integrated peer support to develop an intervention for persons whose mental illnesses, alcohol and drug use, and criminality posed serious barriers for their community stability and participation | NA | Group classes on social participation and community integration, followed by projects to foster gained valued social roles alongside peer mentor support. Peer mentors encouraged participants to maintain their sobriety by offering examples of their own struggles and recovery work and providing social support and friendship to them. Peer mentors supported participants by helping them to identify goals and set priorities for achieving them, sharing their own perspectives and coping strategies as people who have “been there,” and advocating for participants’ access to social services, employment, education, and housing. A second eight-week valued-roles component encouraged participants to contribute to their communities by drawing on their life experiences and skills gained through the classes and contacts with community presenters. Participants designed and participated in education-focused projects, such as teaching police cadets about consumers’ encounters with police officers and conducting a fundraiser for a local non-profit organization. | Project directors led classes (no further details). Peer mentors: completed training on confidentiality, the client engagement process, cultural competence, and the distinctive roles of criminal justice and mental health treatment system personnel. | Face-to-face (either group or individual) |
| Russinova 2018 | Employment | Vocational empowerment photovoice vs wait-list | A peer-run photography-based group intervention. | Is a manualized intervention and includes a work-group for the clients and a corresponding leader's guide for the group peer-leaders. Is based on photovoice methodology which is a process in which individual need cameras. | Each session combines psychoeducational information, exercises, and group discussions relevant to pursuing employment services and opportunities. There are 10 weeks which focus on 10 different themes: introduction to VPE, my working life, writing my photovoice narrative, my vocational values, my vocational identity, costs and benefits of a working life (2 parts), vocational supports and services, setting vocational goals and my vocational journey. The VEP program integrates rehabilitation readiness technology specific to the development of one's vocational identity and vocational goals with photovoice methodology. Photovoice methodology involves a process in which individuals use cameras to photograph objects or events in their daily lives that are relevant to a given research topic and generate narratives for these pictures through group discussion. The following elements of methodology were embedded in the VEP curriculum such as overview of the process, guidelines about the use of camera and photography ethics etc. The VEP program involves two separate photovoice assignments: 1) Picturing work: participants take pictures and write narratives describing their thoughts about work and people working 2) My vocational identity -guides participants to portray their self-perceptions as workers and work aspirations. The discussion of pictures and writing of narratives is informed by the "SHOWED" guidelines which include prompts such as what do you see here, what is really happening here and how does this relate to our lives. Participants are supposed to create at least one piece for each photovoice assignment. | VEP was developed and standardized with input from individuals with a lived experience, peer leaders, providers of employment services and other experts and advisors. | Group sessions. |
| Sacks 2004 | Offending | Prison Modified Therapeutic Community vs Mental Health Treatment program | Personal Reflections, a modified TC residential treatment program, uses a cognitive–behavioural curriculum within a foundation of TC principles to change attitudes and lifestyles in three critical areas: substance abuse, mental illness, and criminal thinking and behaviour. | NR | Part 1 TC: The program includes psycho-educational classes, cognitive–behavioural protocols, medication, and therapeutic interventions. The cognitive–behavioural elements help the inmate to examine how he uses his interpretation (or misinterpretation) of events to influence his feelings and justify his criminal behaviour, and provide him with tools that enable him to recognize and modify distorted perceptions and inappropriate responses. Mental health status is monitored daily; the type and dosage of medication is evaluated weekly. The therapeutic interventions include core groups to discuss personal issues and modified encounter groups to address maladaptive behaviours and foster personal responsibility, with the peer group providing feedback and support. Part 2 Aftercare TC: Activities revolve around basic skills (including meal preparation, banking, use of community resources such as libraries), relapse prevention/triple trouble recovery (substance abuse, mental illness, and criminality), medication and symptom self-management, and emotional and behavioural coping. Mental health counselling, medication, and psychiatric services are provided by a local mental health centre; medication type and dosage are evaluated weekly. | NR | Part 1& 2: face to face residential setting, Individual and group activities |
| Sacks 2012 | Offending | Prison Modified Therapeutic Community vs Standard care | Focus on triple recovery (substance use, mental health) - in conjunction with the overall TC focus on whole person change, along with the promotion of prosocial values within healthy social networks, were the basis for the expectation that criminal behaviour would be reduced. | NA | Weekly group psycho-educational classes - address the interrelationship between mental disorders and substance abuse. Weekly group and individual counselling in relapse prevention/triple recovery, symptom self-management, emotional and behavioural coping, and basic skills training (e.g., budgeting, use of community resources). The group intervention format predominated, but each participant received individual counselling and case assistance from counselling staff at least weekly, or more frequently, if needed. Daily medication monitoring and weekly psychiatric services were provided on-site, and MH counselling was available | Most groups conducted by parole officers. No details on who delivered the psychiatric counselling services. No involvement by research staff. | Face-to-face (either group or individual) |
| Salzer 2016 | Rights | Peer-delivered core services of Centres for Independent Living (CILs) vs TAU | Emphasises the civil rights of individuals who experience disabilities and offers a paradigm shift in how supports are provided. | A standardised script was used to assess unmet develops and assist the peer specialist who had received a certification (CPS). The script included a list of 25 areas in which support could be provided like housing and transportation. | Participants were contacted by phone by the CPS on 3 different days at 3 different times to schedule the first session. If the CPS was unable to schedule a meeting, a letter was sent to the participant to reiterate the services they were eligible to receive at the CIL.  First session - an overview of the philosophy and history of Centres for Independent Living and the score services the CIL offers e.g. information and referral, independent living skills training, peer support and advocacy.  Second session - A standardised script was used to assess unmet needs and assist the CPS and participant in identifying where they would like support.  The list included 25 areas such as housing, transportation and education and was used as a tool throughout the intervention as the CPS would start each session by checking in on progress within each unmet need that was previously identified. | Individuals who received certification as a peer specialist (CPS) from a well-known CPS training organisation with a 75-hr training curriculum and performance standards that need to be achieved. The CPS received training and supervision in the CIL core services philosophy and model from peers with disabilities in the organisation. | Face-to-face and individually. |
| Sanches 2020 | Employment | Boston University approach to psychiatric rehabilitation vs active control condition | Designed to help individuals with SMI achieve and retain goals in four rehabilitation domains: housing, education, work and social contact. Example goals: working 1 day a week as a volunteer or wanting a certain number of hours of paid administrative work | NR | There are four phases: exploring rehabilitation goals, choosing them, getting them and keeping them. While the rehabilitation process is facilitated by a practitioner, the goal and the pace are directed by the person with SMI. No clearly predefined goal is required in order to start. | 28 trained social workers, nurses or employment specialists who had completed additional training in BPR. | Face-to-face and individually. |
| Schene 2007 | Employment | Adjuvant occupational therapy + TAU vs TAU | Rationale: for patients for whom depression is related to impairment in occupational functioning, recovery may stagnate if 'work' is not part of the treatment plan, therefore an OT intervention aiming to restore and improve occupational functioning may be of benefit. | NA | OT consisted of 3 manual-based phases: 1) Diagnostic phase (4 weeks) : 5 contacts with a detailed occupational history, video observation in a role-played work situation, contact with an occupational physician from the patient's employer and a plan for work re-integration. 2) Therapeutic phase (24 weeks) - 24 weekly group sessions (8-10 patients) and 12 individual sessions. | The additional OT intervention was undertaken by two skilled occupational therapists. |  |
| Segal 2010 | Rights | Self-help agencies and community mental health agency services, vs community mental health agency services only | Aims to promote and assist recovery for people with serious mental illness. | NR | SHA - open 5.3 days a week and served 43 clients a day which provided consumer-operated services guided by a self-help ideology. Common service elements include peer support groups, material resources, drop-in-socialization etc. Services included help in obtaining survival resources, money management, case management, counselling, peer counselling etc. All SHAs provided physical space for socialising and developing ongoing peer support networks. CMHAs - county mental health organisations providing outpatient mental health services for people with serious mental illness and included help such as assessment, medication review, individual and group therapy, case management and referral. | NR |  |
| Sheridan 2014 | Social Isolation | Supported socialisation vs control | The intention of the intervention, completed over a 9-month period, was to promote a ‘friendship’ between a community volunteer and mental health service user comparable with ordinary social friendship and existing outside the mental health care system. | NA | The intervention group (Group A) was matched with a volunteer partner and undertook social/leisure activities. Stipend of 20 euros a month. | Community volunteer (attended a one-day training program) | Face to face - one to one |
| Shern 2000 | Housing | Community outreach (Choices) vs Treatment as usual | 1) Outreach and engagement to develop relations between Choices staff and the homeless individual 2) in-community and on-site rehabilitation services to assist individuals in finding and maintaining community-based housing. | NR | 1. Outreach and engagement, designed to foster the development of rudimentary relationships between Choices staff and homeless individuals. 2. Invitation to attend and join the Choices Centre, a low-demand environment where desirable resources (e.g., showers, food) were available for only the experimental study participants from 7 AM to 7 PM daily. Participation in structured group activities was not required, but assistance was available to anyone requesting help in obtaining health, mental health, dental, and social services and in developing and implementing individual rehabilitation plans. Additionally, the centre provided an opportunity for members to meet new friends and socialize. 3. Respite housing in 10-bed, informal church-based shelters or in blocks of YMCA rooms rented by the program and overseen by program staff. 4. In-community and on-site rehabilitation services to assist individuals in finding and maintaining community-based housing. | 6 rehabilitation specialists: extensive training and ongoing supervision from Boston University personnel and respite staff, many who had themselves been homeless. A psychiatrist visited the program weekly for informal consultations A public health nurse 8 hours per week. | Face to face, format NR |
| Silverman 2014 | Social Isolation | Live music therapy and Education vs Education only | Live music provided by a music therapist using an educational approach to illness management and recovery may facilitate the development of trust and increase perceived social support, identified as important treatment outcomes for acute care psychiatric inpatients that may lead to enhanced compliance, coping, and recovery. | Lyric sheets to the song “Runaway train” by Soul Asylum | 1) ask participant to state name and favourite food with a 12 bar blues progression in the key of E played on an acoustic guitar. 2) sing "runaway train" by Soul Asylum using acoustic guitar for accompaniment and note any lyrics can relate to 3) Scripted educational lyric analysis session using song lyrics focused on social supports e.g. "line 1 reads call you up in the middle of the night-who might we call when overwhelmed by stress?" | Qualified music therapist with 12 years psychiatric clinical experience | Face to face, group meetings |
| Stergiopoluos 2015 | Housing | Housing first plus integrated case management vs treatment as usual | Housing First as defined in the Pathways to Housing and Street to Homes approaches creates a recovery-oriented culture that puts participant/tenant choice at the centre of all its considerations with respect to the provision of housing and support services. It operates on the principle that all homeless individuals with mental illness should be offered the opportunity to live in permanent housing of varying types that is otherwise available to people without psychiatric or other disabilities. Assertive in-reach and outreach identifies and engages potential participants avoiding any coercive tactics | NA | The intervention was scattered-site supportive housing with mobile, off-site ICM services,14 offering rapid, low-barrier permanent housing in independent units with supports fostering participant empowerment, choice, personalized goals, hope, and resilience. Participants paid up to 30% of their in- come toward rent, with a monthly rent supplement of CAD $375 to CAD $600 (dependent on study city; to convert to US dollars, multiply by 0.984) paid by the program directly to landlords. | The interventions are delivered by 12 existing service agencies who were the successful applicants from each of the five pre-selected cities to a request for proposals that was issued by the Mental Health Commission of Canada - Technical assistance and training on the Housing First intervention is provided on an ongoing basis by a centralised team of expert | NR |
| Susser 1997 | Housing | Critical time intervention vs usual services | Designed to prevent homelessness by enhancing the continuity of care for individuals being discharged from institutional to community living. CTI has two components. The first is to strengthen the individual's long-term ties to services, family, and friends. The second is to provide emotional and practical support during the critical time of transition. | NA | First component: Each man was assigned to a CTI worker to implement a plan of transfer of care and is focused on specific areas of potential discontinuity that were related to the risk of homelessness for that individual. CTI work entailed visiting the family home or community residence, being present at appointments, and locating patients and giving advice in times of crisis, give support and advice to patients and caregivers, mediate conflicts, help negotiate ground rules for relationships. Second component: during the first 2 weeks after discharge the CTI worker spent time with the client in the community and observed his physical and social surroundings and daily habits. Subsequent support was individually tailored. | The CTI worker did not need to have a professional degree (each worker was supervised by a psychiatrist or other mental health professional) but did need to have experience working with this population and enough "street smarts" to work with these men in the community. | Face to face, individually |
| Terzain 2013 | Social Isolation | social network intervention + TAU vs TAU only | Although many of the activities offered by day care services include socialization, by and large they are limited to interactions with other patients; nonetheless, it is a reasonable assumption that socializing with healthy people in a naturalistic setting may influence, to a greater extent, patients’ self-esteem, as well as their abilities in communication, empathy, and reciprocity than those taking place within an institutional setting, however informal. | NA | Staff identify possible areas of interest for the patient, and propose one or more social activities, specifically the activities had to take place outside the services' resources and with members of the community at large. | Community mental health service staff or family members, neighbours or volunteers: no detail on training given | Face to face, individual treatment, activities were in groups with people from the community |
| Tinland 2020 | Housing | Housing first + ACT vs treatment as usual | A recovery-oriented approach for people who use homeless with severe mental disorders improves hospital and emergency department use. | ACT model manual | Participants were offered scattered housing after their inclusion - they had some choice in the location and type of housing. They paid a maximum of 30% of their income as rent, depending on their resources, with the rest paid by the program. Individuals were firstly subtenants of their flat, becoming thereafter tenants through a lease transfer when they had enough resources. According to the HF model for a high level of needs, the MDT team followed an assertive community treatment model which is a recovery-oriented approach. | MDT team - social worker, nurse, doctor, psychiatrist, peer worker | 10:1 client-staff ratio |
| Tsemberis 2004 | Housing | Pathways housing first vs continuum of care | The program is based on the belief that housing is a basic right and on a theoretical foundation that includes psychiatric rehabilitation and values consumer choice. Pathways is designed to address the needs of consumers from the consumer’s perspective. | NR | Patients are offered housing without any prerequisites for psychiatric treatment or sobriety.  They are then offered through multidisciplinary ACT services 24-hour support. Participants can choose what support to make use of (e.g. they can refuse clinical services altogether). | Multidisciplinary mental health teams (includes social workers, nurses, psychiatrists, and vocational and substance abuse counsellors) | face to face. |
| Twamley 2019 | Employment | Compensatory cognitive training vs enhanced supported employment | Targeting cognitive functions e.g. memory, attention, learning/memory and executive functioning for people with SMI who are seeking work | Is a manualized intervention so there is a manual available. | CCT teaches skills and strategies for implementing the skills to compensate for cognitive difficulties commonly observed in SMI. There are four modules: prospective memory, conversational and task vigilance, learning and memory and cognitive flexibility and problem-solving. The CCT condition participants also received supported employment services as indicated by the need of the individual participant for the duration of the study. | CCT was delivered by a master's level employment specialist | Individually |
| van Beurden 2017 | Employment | Occupational physician’s intervention vs TAU | a peer-learning group which is based on enhancing occupational physicians' guideline adherence on sickness absence duration in workers with CMD | A detailed description of the intervention has been published elsewhere (can be found in the references). | According to the guideline, the guidance of a worker who is sick-listed with CMD starts with a problem orientation and an OP's diagnosis. Next, the OP evaluates the worker's recovery and RTW process by monitoring and enhancing the workers' problem-solving capacity according to the 3-phase model of Meichenbaum. If the recovery process stagnates, the OP uses CBT techniques to enhance the worker's problem-solving capacity. The workers in the intervention group received guidance from an OP who had received the intervention to enhance OPs' guideline adherence. The intervention focuses on barriers that hinder Ops from using specific recommendations in this guideline in practice. According to the model of Cabana et al, guideline adherence can be affected by 3 main clusters of barriers: lack of knowledge, negative attitudes and external barriers. The OPs exchanged ideas and solutions to overcome perceived barriers, drew up joint action plans on how to implement these solutions in their daily practice and tested the suggested solutions in daily practice. | The role of the OP is to monitor the process of sickness absence and RTW, to facilitate communication between workers and their employer and supervisor, to provide information and advice to the employer, supervisor, human resource management and co-workers on how to support the worker. | Peer-learning groups |
| Vauth 2005 | Employment | Computer assisted cognitive strategy training + Vocational rehabilitation vs vocational rehabilitation | Researchers have consistently found that people with schizophrenia score more poorly than others on a wide array of cognitive tasks. Even if atypical antipsychotic drugs as a group are superior to typical neuroleptics with regard to cognitive function, many of these impairments are not entirely normalised by the newer atypical neuroleptics. Cognitive remediation focuses on the improvement of these cognitive impairments by repetitive laboratory-based exercises directly related to the cognitive process being trained and the building of compensatory cognitive strategies. Although studies have shown increases in discrete cognitive functions, there are few studies looking at how such interventions may be helpful for improving real-world outcomes such as employment. | NR | Vocational rehabilitation consisted of graduated job placement in different training sites for up to 15 hours per week with increasing demands, detailed weekly work performance feedback and individual vocational counselling CAST focused on deficits in sustained and selective attention, seal memory, planning. CAST comprises three steps based on Anderson’s Adaptive Control of Thought Model. First, components of strategies are discussed. Then, there is repeated practice of the strategies on prototypical situations. Finally, participants are trained about how the strategy can be generalised to new situation. | NR | Group of 6 to 8 people |
| Vlasveld 2013 | Employment | Collaborative care for major depression vs TAU | Sickness absence and return to work are multifactorial and influenced by a range of different factors such as mental health condition, personal characteristics of the worker and environmental factors such as the workplace and the healthcare system. To achieve a more rapid return to work as well as a reduction in depressive symptoms in sick-listed patients with MDD, treatment needs to focus on both symptom reduction and the process of returning to work. | NR | The collaborative care intervention contained sessions of PST, which is a brief, structured psychological intervention aimed at teaching the worker problem solving skills. The intervention also featured a manual-guided self-help programme based on cognitive restructuring techniques and return to work and healthy lifestyle advice. The intervention also included a workplace intervention, and depending on patient preference, prescription of antidepressant medication. The workplace intervention consisted of a work-place assessment and work adjustment. In the workplace intervention, the employer and the worker both pointed out barriers for RTW, brainstormed possible solutions and made a plan for implementation of the solutions. | OP-CM (case manager). They received a 2-day training prior to the study, and supervision during the study, in order to be able to fulfil the role of CM. | Workplace intervention was delivered with the employer where the OP-CM acted as a mediator No details on other elements of the intervention |
| Volker 2015 | Employment | E-Health cognitive web intervention vs TAU | e-health module aims at changing cognitions of the employee with regards to return to work whilst a decision aid supports the occupational physician with advice regarding treatment and referral options e-Health is a highly available, low-threshold, low-cost intervention. | E-health module Return@work including 5 modules: 1) psychoeducation, 2) module aimed at cognitions with regard to RTW while having symptoms (based on CBT principles), 3) module aimed at increasing problem-solving skills with problem-solving treatment exercises, 4) a module for pain and fatigue management and for reactivation, and 5) a module for relapse prevention. | E-health module Return@work including 5 modules: 1) psychoeducation, 2) module aimed at cognitions with regard to RTW while having symptoms (based on CBT principles), 3) module aimed at increasing problem-solving skills with problem-solving treatment exercises, 4) a module for pain and fatigue management and for reactivation, and 5) a module for relapse prevention. The occupational physicians received automated email messages that were based on a decision aid with the principles of stepped collaborative care. The decision aid supported the occupational physicians in the sickness guidance of the employees, in the monitoring of symptoms, functioning, and RTW. | E learning modules (web-based) | web-based - employees worked though Return@work individually. As in usual sickness guidance, the occupational physician and employee met each other face-to-face on a regular basis. |
| Yamaguchi 2017 | Employment | Cognitive remediation + supported employment vs usual employment services | To improve occupational outcomes. | A computer and computer software were needed for the cognitive remediation program called "Thinking skills for work program" which was referenced. | The participants received cognitive remediation in psychiatric day-care or community employment service agencies during the first 3/4 months - it provides social skills, recreation and a daytime place to stay. Community employment service agencies in general provide a long-term group-based work readiness training for people with mental illness before they undergo a job search. The cognitive remediation programme was given called "Thinking Skills for work program" which was to be used using CogPack software and involved two CogPack sessions per week over 12 weeks and it included tasks like attention, concentration, psychomotor speed, learning, memory and executive function. After one of CogPack session each week, participants engaged in hour-long verbal group sessions during which they discussed the importance of cognitive skills, performing activities of daily living and the development of compensatory strategies for managing persistent cognitive problems. The verbal sessions would link the gap between the individual's learning outcomes in the computer-based cognitive remediation and the employment/social skills in their lives. Supported employment services were then provided to participants after completion of cognitive remediation and these services partly incorporated the IPS model e.g. place-then-train approach, employment specialists in treatment teams, services based on individual's preferences and time-unlimited support. The services particularly included strength-based assessment, outreach services for living and work support, rapid job-search, family support and support for job continuation. | Staff received 1-day training for five times to learn skills for cognitive remediation and supported employment | Digital, face-to-face, group and individual. |

Appendix X continued: Further characteristics

| Study ID | Domain | Treatment comparison | Intervention Setting | When and How Much | Tailoring | Modifications | How Well-Planned | How Well-Actual |
| --- | --- | --- | --- | --- | --- | --- | --- | --- |
| Aubry 2016 | Housing | Housing First + ACT vs TAU | Residential | Length of access to housing NR At least one weekly visit by ACT staff. | NR | NA | NA | Two assessments of program fidelity conducted during the study found good fidelity overall, with 78% of the 38 fidelity scale items rated higher than 3 on a 4-point scale on the second fidelity assessment, 24 to 29 months after the start of the programs |
| Bejerholm 2017 | Employment | Individual enabling and support vs TAU | Outpatient | Phases 1 and 2: 1-2 months. Phase 3: until employment is reached. Phase 4: remaining time. Phases 1, 2 and 3 are approximately 1-hour per week while phase 4 requires 20 minutes per week. | The extent of the counselling is individualised to fit the intervention need for the participants. | NR | Delivery was assessed at 6 and 12 months using the supported employment fidelity scale and 3 questions on motivational, cognitive and time-use support. | At 6 months, 109 points were reached on the supported employment fidelity scale and at 12 months, 106 points were reached -there was a good fidelity. The individual enabling and support fidelity increased from 12-14 points between assessments. |
| Bell 1993 | Employment | paid work vs unpaid work | Veterans affairs medical centre | 20 hours a week paid employment | Options for employment offered | NA | NA | NA |
| Bell 2003 | Employment | Paid work plus behavioural intervention vs paid work only | Veterans affairs medical centre | 60-minute weekly group feedback sessions the work placement was 20 hours a week for 26 weeks | Options for employment offered Feedback was personalised | NA | NA | NA |
| Bell 2005 | Employment | Work therapy + neurocognitive enhancement vs work therapy | Veterans affairs medical centre | 6-month intervention 15-20 hours a week paid work weekly group meetings weekly group social information processing meetings | Options for employment offered Feedback was personalised | NA | NA | NA |
| Bell 2008 | Employment | neurocognitive enhancement + vocational rehabilitation vs vocational rehabilitation only | Community | 12 Month intervention. Weekly group meetings up to 20 hours work a week up to 10h a week cognitive exercises on a computer | a guiding principle was to optimize the balance between challenge and frustration. For that reason, occasional alterations in the curriculum (such as changing tasks before completion) would be made in order to preserve that optimal balance. Staff was vigilant to participant's engagement in the activity. If a participant began to fall asleep or appeared to be responding randomly, staff would intervene. In that way, quality of the training was preserved | NA | Apart from the deliberate deviation of transitional funds, the vocational program was committed to adhering to the principles of the IPS model. This modified IPS program received a score of 62 out of 75 (83%; “Fair” rating) on the IPS fidelity scale when rated by three independent raters including Gary Bond Ph.D. (personal communication), who interviewed the program staff and vocational specialists. Adherence to these procedures was reinforced weekly, and written material from the meetings (such as WBI feedback graphs and participant presentation outlines) were checked for conformity to the principles and completeness | This modified IPS program received a score of 62 out of 75 (83%; “Fair” rating) on the IPS fidelity scale when rated by three independent raters including Gary Bond Ph.D. (personal communication), who interviewed the program staff and vocational specialists. |
| Bell 2018 | Employment | vocational rehabilitation + cognition remediation vs vocational rehabilitation + cognitive games | Cognitive training lab | 6-months. | NR | NR | The IPS programs at both institutions were independently evaluated for fidelity. | The fidelity for IPS programs was in the acceptable range. |
| Beutel 2005 | Employment | Occupational training integrated into psychodynamic treatment vs TAU | Inpatient | 2 sessions per week. | Work assignments are individualised according to the patient's capacities, skills and interests. | NR | NR | NR |
| Boevink 2016 | Social Isolation | TREE Recovery programme + TAU vs TAU | Community and residential | Self-help groups - two-hour meetings every fortnight for 52 weeks for late starters and 104 weeks for early starters | NR | N/A | NA | NA |
| Burnam 1996 | Housing | Social model residential treatment program vs no intervention | Community and residential | 24-hour, 7 day per week program | After the second three-month period (Phase 2), those who wished could continue to engage in program activities of their choosing. | NA | NR | NR |
| Burnam 1996 | Housing | Social model non-residential treatment program vs no intervention | Community and residential | 1pm-9pm 5 days a week | After the second three-month period (Phase 2), those who wished could continue to engage in program activities of their choosing. | NA | NR | NR |
| Castelein 2008 | Social Isolation | Guided peer support VS TAU | Community | 16 sessions of 90 min biweekly over 8 months. | NR | N/A | NA | NA |
| Chandler 2006 | Offending | Integrated dual diagnosis treatment post-custody vs usual post-custody services | Community | Time-unlimited (no other details) | NR | N/A | Items were rated two years into the program by the lead author, who was the project evaluator, and an independent psychiatrist well-versed in co-occurring disorders treatment. No strategies to maintain to improve fidelity mentioned. | Mean program fidelity was 4.1 and 4.0 out of 5 for the two raters. |
| Christensen 2019 | Employment | IPS with enhancements vs TAU | Outpatient | No details on IPS. Training: 24 group-based sessions + 12 sessions in coping strategies + six work-related social skills training sessions | NR | N/A | The IPS employment specialists will be evaluated by trained external reviewers who will use the IPS fidelity scale to ensure high fidelity and adherence to evidence-based practice [28]. The evaluation will take place 6 months after trial start and thereafter every sixth month until high fidelity is demonstrated. Subsequently, an annual evaluation will be performed. | All sites demonstrated good fidelity to the IPSE manual, with scores between 21 to 29 points on the 30-point scale. However, 57 of 238 participants (23.9%) in the IPSE group did not attend the sessions with cognitive remediation, and the mean (SD) number of cognitive training sessions attended was 9.6 (9.7) of 30 sessions. |
| Conoley 1985 | Social Isolation | Reframing vs Waitlist control | Community | Two 30-minute sessions one week apart | NR | N/A | NA | NA |
| Cook 2008 | Employment | Supported employment vs TAU | Community and residential | NR | NR | N/A | A supported employment fidelity assessment applicable across models was developed and administered independently by research staff at the point of each program’s maturity. As a manipulation of fidelity, the amounts and types of vocational services delivered were compared for the two-year study period across all sites. | All experimental conditions exhibited high levels of fidelity to the model Experimental group subjects received significantly greater amounts than controls of 10 different employment services, with no significant differences in clinical services received. |
| Cosden 2005 | Offending | Mental health treatment court vs TAU | Community | ACT case management had an 18-month time limit | NR | N/A | NA | NA |
| Cusack 2010 | Offending | Forensic ACT vs TAU | Community | NR | NR | n/a | NA | The program had high fidelity to the ACT model with Dartmouth Assertive Community Treatment Scale (Teague et al. 1998) scores of 4.5 and 4.6 (range 1–5) during its first and second years of operation. |
| Davidson 2004 | Social Isolation | Matched with a volunteer partner who had a personal history of psychiatric disability vs Matched with a volunteer partner who had no history of psychiatric disabilities vs Not matched with a volunteer partner | Community | 2-4 hours per week for a period of 9 months. | Decisions as activities, meeting times and other arrangements were left to the discretion of participants and their partners. | NR | NR | NR |
| Davis 2012 | Employment | IPS vs TAU | Community | NR | NR | N/A | The national consultant also conducted fidelity monitoring, which included reviewing the IPS specialist’s caseload, meeting with veterans from both IPS and VRP interventions, interviewing participants’ clinical providers, interviewing participants’ employers, and rating the adherence of the IPS intervention with the Supported Employment Fidelity Scale (26). The national consultant also conducted a Supported Employment Fidelity Scale assessment of VRP to ensure that the ratings were low—that is, that VRP remained different from the IPS intervention as was intended by the study design. | The IPS fidelity monitor scored the Supported Employment Fidelity Scale as 55 at the onset of the study, and this score steadily improved to within a range of 58–65 during the study, with a mean±SD score of 61.2±2.1. On this scale, 66–77 is regarded as good IPS implementation, 56–65 as fair implementation, and ≤55 as “not supported employment.” The VRP was consistently rated by the fidelity monitor as ≤40 during the study, which is appropriate for this intervention. |
| Davis 2015 | Employment | Mindfulness based stress reduction (Mirrors) vs Intensive support control | Outpatient | Twice weekly, 75-minute session for 16 weeks. | NR | N/A | The primary fidelity assessor was a graduate psychologist who had a personal mindfulness practice and completed an MBSR class and one of the three training programs required for teacher certification by the Centre for Mindfulness. She rated 1 randomly selected group session per month for a total of 17. Feedback was provided on a timely basis so that the first author could take corrective action. | fidelity ratings were found to exceed the minimum standard set at 2.5, with a mean of 2.62 (SD 􏰀 0.21) |
| Davis 2018 | Employment | IPS vs transitional work | Outpatient | NR | NR | N/A | The study’s IPS fidelity monitor (R.T.) performed a biannual, on- site IPS fidelity review using the Supported Employment Fidelity Scale. This scale is scored from 15 to 75, with higher scores indicating better implementation (score of >66 is good implementation, 56-65 is fair implementation, and <55 indicates not IPS) | The mean Supported Employment Fidelity Scale score was 55 within the first 3 months and subsequently ranged between 63 and 69. Most sites maintained good or higher implementation. Two sites had difficulties with IPS implementation however, corrective actions yielded improvement attaining fair to good implementation. The transitional work services were rated as not IPS (i.e., mean, 26-32 [range, 18-41]) on the Supported Employment Fidelity Scale, indicating that transitional work did not drift toward IPS. |
| de Waal 2019 | Victimisation | Self-wise, other-wise, street-wise (SOS) training + TAU vs TAU | Outpatient and inpatient | 12 twice-weekly 90-minute sessions including a 15-minute break, within a 6-week time frame. Sessions were delivered by any 2 of 14 trained therapists, with a maximum of 8 participants per session. | The treatment groups are semi-closed: at the first session of each module, new participants can join the group and start the training so the other in which participants follow the module varies. As the group compositions change, each module starts with becoming acquainted with new group members. | NR | Therapists were asked to rate how well they adhered to the treatment protocol on a scale of 0-10 after each session. | The therapist rating resulted in an average of 9.2 (standard deviation = 1.0). |
| de Weerd 2016 | Employment | Work-focused CBT with convergence dialogue training vs work-focused CBT only | Outpatient | Frequency of CBT sessions is high (6 sessions in 4 weeks) at the onset of therapy. The CDM was one meeting approximately 90 minutes. | NR | NR | NR | Dijkgraaf-Hartland et al., showed good protocol adherence by therapists with at least 80% of the procedures described in the protocol to be executed. |
| Elbogen 2016 | Money | $teps for Achieving Financial Empowerment ($AFE) (psychoeducational money management program) vs TAU | NR | Was a brief intervention of 1-3 hours. | NR | NR | NR | NR |
| Elison 2020 | Housing | Manualized treatment model for co-occurring mental illness and substance use disorders (MISSION-Vet) vs TAU | Community and residential | 1 hour each week for 40 sessions to occur over the course of 9-12 months | The original intervention was to achieve weekly visits but ultimately, veteran choice dictated how often and how long peers would visit. | NR | An as-treated analysis was conducted to examine the impact of the treatment when it was delivered by peer specialists who were able to follow the treatment protocol as intended. The GAMM analysis was performed to estimate treatment effect profiles for all control veterans compared to treated veterans who were assigned to 1 of the 3 peer specialists who more closely adhered to the service delivery protocol. The determination of the protocol adherent peers was based on (1) the peer had sufficient job tenure to deliver the intended 4 sessions (2) peer notes in the medical record and supervision records that indicated delivery of structured and non-structured sessions was adherent to the intervention protocol. | There were 3 peers according to the as-treated analysis who had sufficient job tenure to provide services until the veterans’ treatment period ended and who demonstrated greater adherence to the study protocol. |
| Erickson 2020 | Employment | IPS + TAU vs TAU | Outpatient | 1 year of employment support. | NR | NR | There were two fidelity audits at the end of years one and two of the study and the results of the audit were used to improve the quality of IPS services. The fidelity author was trained by the IPS centre in Dartmouth and has extensive experience with IPS fidelity reviews. He used the supported employment fidelity scale which has 25 items grouped into ratings of staffing, organisational features etc. There is a 1-5 Likert-type scale and total scores at or above 115 are considered exemplary, 100-114 are rated as good and 74-99 are considered fair. | The two fidelity checks conducted by a trained independent auditor suggested "good" implementation. |
| Fletcher 2008 | Housing | Integrated assertive community treatment vs standard care | Community | Briefly mentions up to 30 weeks. | NR | NR | Research staff assessed treatment fidelity of IACT condition using a revised and expanded version of the Dartmouth Assertive Community Treatment Scale (DACTS) at two points in time, 12 and 24 months after project initiation. The 35-item instrument contained 26 items that focused on fidelity to the ACT model and nine new items that focused on implementation of substance abuse treatments including motivational interviewing, skills approach to substance abuse counselling and comprehensive substance abuse assessment. | The IACT condition had moderate to high scores on fidelity to the original ACT model. |
| Fowler 2019 | Employment | Social recovery CBT + TAU vs TAU | Community | Participants received a mean of 12 sessions (SD=7) over 9 months. | NR | NR | Therapy in both centres was supervised by experienced CBT specialists. Adherence and competence were monitored using tape recordings and individual and group supervision. | NR |
| Gelpkopf 1994 | Social Isolation | Comedy films vs Variety of film genres | Inpatient | 3 months. Two different films were projected. On most occasions, twice daily (i.e., four presentations per day), 5 days a week. | NR | N/A | NA | NA |
| Glynn 2004 | Social Isolation | Skills training + generalization vs skills training only | Outpatient | Clinic-based skills training: twice weekly for 90 minutes each visit for the first 24 weeks, once weekly for 90 minutes for the next 12 weeks, then weekly for 60 minutes for 24 weeks (a total of 60 weeks of intervention). Additional in vivo amplified skills training: approximately 75 minutes weekly for generally the first 52 weeks of the study and then on a declining contact basis for the last 10 weeks. During engagement in treatment, when difficult skills were being taught, and in times of crisis (e.g., unexpected homelessness, family illness), in vivo amplified skills training sessions might be held more than once a week. The entry of new study subjects was timed so that patients could begin the study in cohorts of approximately six to eight patients, with three to four assigned to each condition. Participants saw their care manager/study coordinator weekly for a brief status check and to receive their study medication. Participants were seen by their study psychiatrist at least monthly for a review of symptoms and side effects | During engagement in treatment, when difficult skills were being taught, and in times of crisis (e.g., unexpected homelessness, family illness), in vivo amplified skills training sessions might be held more than once a week. | NA | Module trainers were supervised in weekly meetings with a master module trainer (K.B.). The supervisor attended one or two randomly selected clinic sessions each month and rated the sessions for adherence to the manual-based micro components of the module, utilizing the Module Fidelity Rating Scale (3). The in vivo amplified skills training supervisor (S.M.G.) met weekly with the trainer to review the patients’ progress and the trainer’s fidelity to the manual. She also went into the community with the trainer at least once with each subject. To assess protocol adherence, the proportion of the 60 scheduled in vivo amplified skills training activities completed was calculated for each participant | Overall, the data indicated that the interventions were conducted as prescribed by the manuals. Significant improvements in all eight medication and symptom management skills, as well as the two composite scores, were achieved from pre- to post training (all p<0.01) on the module tests. The supervisor’s monthly ratings of randomly selected medication management and symptom self-management skills clinic meetings revealed that the facilitators were generally adherent to the skill training manuals. Average scores on the introduction, goal setting, modelling, reaction to difficult situations, quality of instruction, reinforcement, and therapeutic alliance domains were all above 4 on a scale from 1, poor, to 5, excellent, indicating at least “good” skill in training. A review of the in vivo amplified skills training activities summary sheet revealed that across all assigned participants, a mean of 50.87 (SD=16.67, range=0–60) (85%) of the 60 manualized in vivo amplified skills training activities were completed. Among the 23 subjects who completed in vivo amplified skills training, the mean number of in vivo amplified skills training activities conducted rose to 56.96 (SD=5.27, range=53–60) (95%). |
| Glynn 2017 | Employment | IPS plus work skills training vs IPS only | Community | IPS: NR Skills training: twice weekly 90 minutes | To accommodate various hiring dates, the skills training was offered on a revolving basis, so the teaching of the last module was immediately followed by teaching of the first module so that participants could enter the program at any point | NA | In addition to participation, by condition, in weekly cross-site consultation phone calls, each site was evaluated yearly with the original 15-item IPS Fidelity Scale (19).  For WPFM, consultants reviewed written records for each client weekly. | Both sites scored above 65 (scale of 15 to 75 points) on all yearly reviews, indicating good implementation.  For skills training, Twenty-seven (10%) randomly selected WPFM session audiotapes were rated on the Therapist Fidelity Evaluation for Modules (20); 24 (89%) of the sessions had over 80% adherence on both of the subscales. |
| Goldfinger 1999 | Housing | Group housing vs independent housing | Community and residential | Case manager at least once a week. No details on how long the tenancy is | NR | N/A | NA | NA |
| Granholm 2005 | Social Isolation | Cognitive behavioural social skills training vs TAU | Community | 24 weekly 2-hour group psychotherapy sessions, | NR | N/A | NA | The Cognitive Therapy Rating Scale for Psychosis (40) was used to rate therapist competence from 30 videotaped sessions that were randomly selected but stratified according to module. The inter- rater reliability (interclass correlation) of the three raters was 0.85. The mean rating was 43 (SD=7). A score of 30 or more has been viewed as an adequate competence in cognitive behaviour therapy for psychosis (41). |
| Gutman 2009 | Employment | BRDGE supported education programme vs TAU | Community | 12 2-hour lectures followed by 1 hour one to one mentoring | Mentoring is personalised to the student | NR | NR | NR |
| Harris 2017 | Employment | Cognitive remediation and supported employment vs internet information | Outpatient | Participants were asked to log on twice a week for at least 10 hours over a 4-month period. | NR | NR | Adherence was monitored by the research team who provided regular reminders to participants to log on via email. | NR |
| Haslam 2019 | Social Isolation | Groups 4 Health social identity intervention vs TAU | Outpatient | The first four modules took place weekly and lasted between 60-90 minutes. The fifth final module took place one month later. | NR | NR | Facilitators were trained in program delivery and received weekly group supervision, which in addition to using the manualised program, contributed to treatment fidelity. Facilitators were also asked to complete a checklist at the end of each session to determine compliance to the protocol. | Facilitators rated the extent to which they covered key issues and activities in each session with ratings from 1-7 anonymously. The mean adherence ratings on session content indicated facilitators were able to engage in considerable discussion of key elements in each session so there was good adherence to program content. |
| Hasson-Oyayon 2014 | Social Isolation | Social cognition and interaction training + social mentoring vs social mentoring only | Community | 3 times a week 1-hour sessions for 6 months. (SCIT once a week, social mentoring twice a week) |  | NA | NR | NR |
| Hees 2013 | Employment | Adjuvant occupational therapy vs TAU | Outpatient and community | Variable intervention length. 3 problem clarification sessions, 8 group sessions, 4 individual sessions and a follow up session | Personalised to the specific problems that the participant faces in employment. This is established in the problem clarification stage | NR | NR | NR |
| Hellstrom 2017 | Employment | IPS modified for people with mood and anxiety disorders vs TAU | Community | During the first 6 months, the participant and mentor most often meet once a week for 1 to 1.5 hours on average. After 6 months the number of contacts varies and can be by telephone or email. The number and duration of contacts depend on the needs of the participant. | The whole intervention Is tailored to the needs of the participant | NA | To ensure implementation of the IPS-MA method, four fidelity measures were conducted by an independent investigator. Data were collected through multiple sources and focus was on core elements of the method.10 The fidelity scale is a 21-item scale (scores ranging: 0–5 points) with a possible maximum score of 105 points. An organisational index was also developed: a 6-item scale (scores ranging: 0–5 points) with a maximum score of 30 points. | Overall, fidelity results indicated that the method was well implemented, with fidelity scores of 100, 102, 103 and 103, respectively (maximum score is 105) and a general organisational index score of 30 (maximum score is 30) at all four measurements.10 A reason not to reach maximum fidelity score was that comments were made concerning the service of contact to employers (the workplace intervention). All participants were offered this service, but very few agreed to let their employer know about their mental illness; consequently, the workplace intervention was not practised sufficiently |
| Henderson 2013 | Employment | Use of a decision aid + TAU vs TAU | Community | NR | NR | NR | NR | NR |
| Herman 2011 | Housing | Critical time intervention + usual care vs usual care | Community | Nine-month intervention delivered in 3 phases where each one lasts approximately 3 months. | NR | NR | NR | NR |
| Himle 2014 | Employment | Work-related CBT + vocational services vs vocational services only | Community | 8 2-hour sessions held twice weekly over the course of 4 weeks. | NR | NR | Weekly supervisions took place. Also, trained independent evaluators with at least a master's degree and experience in CBT, rated WCBT fidelity using a modified version of the treatment adherence scale for SAD which measures protocol adherence and therapist competence. The schedule included specific modifications reference to vocational content and the ratings ranged from 1 (ineffective) to 5 (extremely effective). A rating of 4-5 is considered within protocol. | The WCBT treatment fidelity ratings yielded an average overall rating of 4.23 which is above thaw within-protocol threshold. |
| Hurlburt 1996 | Housing | Section 8 rent subsidy certificate vs no section 8 rent subsidy certificate | Community and residential | NR | NR | NR | NR | Although half of the participants had access to a certificate (N 181), only those who completed the application process actually received a housing certificate (N=159, or 87.8 percent of those who had access to a certificate). |
| Hurlburt 1996 | Housing | Comprehensive housing services vs traditional housing services | Community and residential | 24 hours a day 7 days a week | NR | NR | NR | NR |
| Kern 2018 | Employment | IPS + errorless learning vs IPS | Community | Participants who obtained jobs were followed for 12 months from the time of their job start date. | An individualized training plan was developed for each participant with the targeted behaviour broken down into its constituent elements. | NR | Meetings were held with the PI weekly or biweekly to document method type and time devoted to implementation. |  |
| Killackey 2019 | Employment | IPS vs TAU | Community | NR | The nature of support to employed participants varied and was largely dependent on participant preferences, needs and also the degree of disclosure about illness to workplaces that participants were comfortable with | NR | As a result of resource constraints, it was not possible to engage an independent evaluator to conduct a fidelity review of our IPS intervention. However, we conducted an audit of our intervention via self-administration of the Supported Employment Fidelity Scale. | This audit indicated that the intervention was in the range of good fidelity |
| Kingston 2018 | Offending | Reasoning and rehabilitation2 + TAU vs TAU | Prison and community | 14 90-minute sessions delivered twice per week. | NR | NR | Brief mention of the fact the original programme has been subjected to a number of positive outcome evaluations demonstrating its value in reducing recidivism. | NR |
| Korr & Joseph 1996 | Housing | Case management vs routine care | Community and housing | Housing length NR. Case management: During the first weeks of client involvement, aggressive outreach includes almost daily visits. | NR | N/A | NA | NA |
| Kukla 2018 | Employment | CBT + cognitive remediation vs vocational support | Outpatient | IVIP - 26 weekly group sessions and individual sessions. The hour-long weekly group sessions centre on a rotating manualized curriculum of 4 2-week modules (total of 8 sessions). Cognitive remediation - 4 different exercises performed for 15 minutes each. | NR | NR | Weekly supervisions took place where group session tapes were discussed, assessment of adherence to the CBT model and feedback on individual fidelity items. Level of adherence to the principles of CBT for individual sessions was assessed using the Revised Cognitive Therapy Scale (CTS-R) conducted by a trained clinical psychologist. Adequate fidelity was defined as a CTS-R score of 36 on the individual version and 21 on the group version with no individual items falling below a rating of 2. The score reflects an average rating above "competent" as compared with an average skilled therapist. | All IVIP therapists maintained at least adequate fidelity during the study period. |
| Lamberti 2017 | Offending | Forensic ACT vs Enhanced TAU | Outpatient | Weekly | NR | NR | NR | The FACT intervention's ACT team component received a baseline score of 4.75 on the Dartmouth Assertive Community Treatment scale indicating high fidelity. The FACTS was used approximately 6 and 15 months after study initiation to assess fidelity to the Rochester FACT model with scores indicating high fidelity (4.69 and 4.61). |
| Lecomte 2019 | Employment | CBT for supported employment vs supported employment | NR | A maximum of 8 1-hour group sessions, twice per week for one month. | NR | NR | Each therapy session was audio-recorded for supervision and quality assurance purposes. | NR |
| Lehman 1997 | Housing | ACT vs usual community services | Community | NR | Individually tailored interventions address needs for long-term housing, income, and daily structure. Chronic medical problems, substance abuse, family and interpersonal issues, and psychiatric problems are also addressed | NR | NR | After the first year of operation, a site visit by an ACT program expert from Wisconsin confirmed adherence to the ACT program model. |
| Lindenmayer 2008 | Employment | Cognitive remediation vs computerized control | Inpatient | Computer sessions were 45 minutes in duration. Each participant took part in 2 hours of computer practice a week and engaged in a once weekly discussion group involving 6-8 participants, with a duration of one hour. Participants received approximately 24 hours of computer-based cognitive exercises in total across the 12 weeks period. | NR | NA | Assessment of fidelity was not reported. | NA |
| Lipton 1988 | Housing | Residential treatment vs standard care | Housing | NR | NR | NA | NA | NA |
| Lloyd-evans 2020 | Social Isolation | Community navigator programme + routine care vs Routine care | Outpatient and community | 10 hourly-long meetings with a Community Navigator and access to up to three group sessions over a six-month period. | The intervention was personalised to each participant with each participant having different social connections and social activities. | NR | Programme delivery was assessed through session logs completed by community navigators and programme acceptability was explored through qualitative interviews with intervention group participants, their family and friends, programme providers and other involved staff. | The process records show the programme was delivered as intended (there was a median of 7 meetings with their community navigator (of a maximum ten) per treatment group. The qualitative interviews indicated good acceptability of the programme to stakeholders. |
| Lysaker 2005 | Employment | Vocational CBT program vs TAU | Outpatient and community | The CBT groups were delivered in eight sessions, with each of the four modules delivered over two weeks. Groups were held once weekly for one hour. The eight sessions were repeated at least three times over a six-month period. Individual CBT sessions were also held once weekly (duration of sessions was not reported) | NR | NA | The authors report that the IVIP has "measures of fidelity" but these are not reported and no details of how fidelity was maintained or improved are provided | NA |
| Marder 1996 | Social Isolation | Social skills training vs supportive group therapy | Veterans affairs medical centre | Twice weekly for 90 minutes for up to 2 years | NR | NR | NR | NR |
| McGurk 2007 | Employment | Supported employment + cognitive training vs supported employment | Community | 45-60 minutes, 2-3 times a week for 12 weeks of cognitive training | Job search planning is based on client’s vocational preferences | NA | NR | NR |
| Mcgurk 2015 | Employment | Enhanced supported employment + cognitive remediation (thinking skills for work) vs enhanced supported employment only | Community | Once to twice a week (24 sessions), no details on time period delivered for | NR | NA | NA | Total scores (mean=48) indicated high fidelity at both sites. Forty of the 57 participants in the Thinking Skills for Work group (70%) completed six or more computer cognitive training sessions and thus were categorized as “treatment exposed.” The treatment-exposed participants completed an average 21.95 (out of 24) computer training sessions, including 25.3 contacts with the cognitive specialist (mean=25.8 hours) over an average of 154.9 days. |
| McGurk 2016 | Employment | Enhanced vocational services + cognitive remediation (thinking skills for work) vs enhanced vocational services only | Community | 24 hours of computer-based cognitive exercises. Sessions required 45-60 minutes to complete with participants usually completing 1-2 sessions per week over a total duration of about 12 weeks. | Once referred, the cognitive specialist with the vocational specialist assessed participants cognitive strengths and weaknesses through a review of their job history and naturalistic observations in the community, collateral reports from the vocational team and employer and family reports. This led to the identification of cognitive enhancement strategies which were reviewed in meetings and these discussions highlighted strengths and motivations for work. | NR | Weekly supervision calls were conducted to review cognitive exercise worksheets to determine progress on the practice exercises and inform strategy coaching, to problem solve engagement or implementation issues, review self-management strategies and integration with vocational services. Site visits occurred every 3-6 months which included observation, feedback and measurement of fidelity to program implementation guidelines. Adherence to the guidelines of the intervention were monitored through a combination of reviewing curriculum of cognitive remediation exercises to ensure performance improvements were demonstrated, regular participation of cognitive specialist in vocational team meetings and through observation of cognitive training sessions done during site visits. |  |
| McHugo 2004 | Housing | Integrated housing vs Parallel housing | Community | NR | NR | NA | Model fidelity assessed using the Dartmouth ACT fidelity scale (DACTS) used by an independent rater with experience in mental health services and fidelity assessment. They made 1-day visits to the programs at 6-month intervals. Written reports made to the treatment teams | The intervention differed from the comparison intervention. Intervention differed in ways planned. |
| Mervis 2017 | Employment | Indianapolis vocational rehabilitation programme vs supportive therapy | Community | It was a 4-month program and each participant was also enrolled in a work placement for up to 10 hours a week. The clinician also facilitated a weekly 45-minute session to provide an opportunity for work feedback and a chance to practice and further develop work-related abilities. | NR | NR | Fidelity of treatment was maintained through weekly supervision meetings with a doctoral-level clinician on the research staff. | NR |
| Milligan-Saville 2017 | Employment | RESPECT manager mental health awareness training vs waitlist | Community | Single four-hour session | NA | NA | NA | NA |
| Morse 1992 | Housing | Continuous treatment team vs outpatient mental health services | Community | Unlimited time, no details of how much | NR | NA | NA | NA |
| Morse 1997 | Housing | Broker case management vs ACT | Community | NR | NR | NA | NA | NA |
| Morse 1997 | Housing | ACT with community workers vs ACT | Community | No time limits. | NR | NA | NA | NA |
| Morse 2006 | Housing | Integrated assertive community treatment vs standard care | Community | Briefly mentions up to 24 weeks. | NR | NR | Research staff also assed the treatment fidelity of the conditions to ideal standards of assertive community treatment, using a revised version of the Dartmouth Assertive Community Treatment Scale (DACTS) developed by Teague, Bond and Drake (1998). Based on monthly observations of team meetings and interviews with clinical staff, research staff rated the IACT program on the DACTS at two points in time (12 and 24 months after project initiation). The DACTS used in the study consisted of 26 items measuring the human resources, organisational characteristics and service operations of the IACT team and each item was rated on a 5-point scale where high scores indicated high fidelity with the ACT model. The research staff also evaluated the IACT program using a similar 5-point scale on 9 pilot items designed to assess the integrated treatment philosophy. | The average DACTS scores were moderately high but some treatment diffusion occurred. |
| Mueser 2005 | Employment | Skills training programme vs TAU | Outpatient | Group skills training sessions were held once weekly for 2 hours until program completion (3 - 4 months). Monthly booster sessions available after completing the program (no further details provided). | (One-to-one sessions provided to participants who missed group sessions) | NA | Measures of fidelity for the intervention are not reported. However, the authors report that although the fidelity of the Work Inc services (received by control and intervention groups) to evidence-based supported. employment models were not "formally assessed", it was judged through contact with staff, confirming that most essential supported employment elements were included e.g. individualized job searches and "time-unlimited" in-job support but there was no integration of mental health and vocational services support | NA |
| Noordik 2013 | Employment | Exposure based return to work intervention vs TAU | Community | NR | Tailored according to participants situation | NA | The OP in the RTW-E group received two days of training in the RTW-E program. Thereafter, we conducted three follow  up tutorial sessions during the inclusion period. During these sessions, difficulties with applying the RTW-E program were discussed with the session supervisor and other participating OP and ideas for practical solutions were exchanged for problems that had arisen. | NA |
| Nuechterlein 2019 | Employment | IPS + Workplace fundamentals module vs brokered vocational rehabilitation + social skills training | Outpatient | WFM - 75 minutes groups weekly for 6 months followed by booster groups of fading frequency over 6 months. 18-months overall treatment? |  | NR | NR | IPS fidelity was good (score 101 on IPS-25) with the major limitations being use of research-based exclusion criteria, an initial medication stabilization period and only one IPS specialist. |
| Okpaku 1997 | Employment | Employment-orientated case management vs TAU | Community | 4 months. The average amount of time the specialists spent on service provision with their clients during the 4-month intervention was 12.7 hours per client (SD=19.1). However, the range was .7 hours to 146.3 hours. | NR | NA | NA | NA |
| Overland 2018 | Employment | work directed CBT and job support programme (At work and Coping) vs TAU | NR | NR | NR | NR | NR | NR |
| Pos 2019 | Social Isolation | CBT for social activation vs TAU | Outpatient | A period of 3 months. Received 8 group sessions of 1/hour twice a week followed by 6 weekly individual sessions of 45 minutes. | During the individual sessions, the choice for a particular combination of cognitive techniques was based and dependent on the individual needs and goals. | NR | Therapists received extensive training by A.B.P. Staring and were supervised by C.J.Meijer and F.Schirmbeck throughout the study. Feasibility of the treatment and treatment manual proved to be good in the pilot study of Staring et al. To ensure adherence to the manual and coherence across sites, sessions were routinely monitored by videotapes and audiotapes. Treatment receipt and enactment was promoted by active participation during sessions, on-site exercises and homework assignments which were discussed at the beginning of the following session to ensure understanding and application and learned skills. | 7 individuals (14.3%) did not start with the group training and were lost pre-treatment. Another 11 of the remaining (26.2%) were defined as non-completers adhering to less than 65% of the training sessions. |
| Pot-Kolder 2018 | Social Isolation | Virtual reality CBT vs TAU | Outpatient | 16 sessions over 8-12 weeks with sessions lasting 1 hour, 40 minutes of which comprised virtual-reality exercises. The remaining 20 minutes were used to plan and reflect on exercises. | The stimuli in the virtual game are directly controlled by the therapist and so personalised treatment exercises are created for each patient. | NR | All therapy sessions were recorded on audiotapes. Experienced CBT psychologists anonymously rated a random selection of sessions (two per therapist) for treatment fidelity with the Cognitive Therapy Rating Scale. | 28 sessions were rated for treatment fidelity and therapists had "good" to "very good" adherence to the protocol and CBT quality (mean 4.5 (range 2.4-5.9). |
| Priebe 2020 | Social isolation | Matched with a volunteer partner who had no history of psychiatric disabilities vs not matched with a volunteer partner | Community | Weekly for a year. | It was personalised in the initial meeting depending on what volunteer would best suit the patient. | NR | NR | NR |
| Rebergen 2009 | Employment | Guideline based care (an activating approach, time contingent process evaluation, and cognitive behavioural principles) vs TAU | Outpatient | NR | Return to work interventions proposed if the cause of mental health problems were work related | NA | To mimic a realistic situation, no activities were undertaken to improve the implementation of the guideline by the OP. After follow-up, guideline adherence was examined in a process evaluation by auditing the medical files. | NR |
| Reme 2019 | Employment | IPS vs TAU | Outpatient | NR | Individualised and continuous job support' according to the IPS manual | NR | The IPS-25 Fidelity Scale was used to assess the quality of implementation (measures adherence to the IPS principles). Teams of trained evaluators follow detailed instructions, and the ratings are done based on interviews, team meeting observations, and document reviews. Total score ranges from 25-125, and critical cut-off for being recognised as IPS is >74. | The program had moderate to high fidelity to the Fidelity Scale, with all teams scoring fair, good or exemplary on fidelity on the second and third assessments |
| Rivera 2007 | Social Isolation | Peer-assisted case management vs Standard case management | Community | 12 Month intervention. Frequency of meetings NR | NR | NA | NR | NR |
| Roberts 2014 | Social Isolation | Social cognition and interaction training vs TAU | Outpatient | delivered in 20–24 weekly, hour-long sessions. The exact duration of the intervention varies based on the speed with which the group moves through the session content. | NR | NA | NA | A total of 73 sessions were rated for treatment fidelity by DRC using a standard scale. The average fidelity rating (of a maximum of 16) for the four SCIT cohorts was 14.7, 14.7, 14.1, and 14.8. The SCIT participants attended an average of 65% of treatment sessions (median = 71.4%). |
| Rodriguez Pulido 2019 | Employment | IPS plus cognitive remediation vs IPS | Community | The cognitive rehabilitation group attended sessions with the Cogpack program every week for 4 months (32 sessions) and the majority of participants performed two sessions of one hour per week. | The sessions with the participants were individualized adapting the levels according to the evaluation of obtained results, making changes and adding tasks according to the individual evolution. | NR | NR | NR |
| Rogers 2006 | Employment | Psychiatric vocational rehabilitation vs enhanced state vocational rehabilitation | NR | three times a week; no more details | Personalised with individual meetings with a vocational specialist | NR | NR | NA |
| Rossler 2020 | Employment | IPS with 55h placement budget vs IPS with 25h placement budget | Outpatient | 55 hours placement budget (vs 25 hours) | NR | NR | Fidelity to the principles of IPS was assessed for each job coach every 3 months with the Supported Employment Fidelity Scale throughout the whole trial. This 15-item scale is a well-researched tool that evaluates the compliance of the service to the IPS principles. High fidelity to the approach was indicated if a job coach reached a score between 66 and 75. | Showing an average of 68 points, job coaches as a group met criteria for good IPS treatment fidelity in this study |
| Rowe 2007 | Offending | Group/peer support vs standard services | Community | Twice-weekly two-hour classes for 16 weeks. | NR | NA | NR | Of the 73 participants, 33 attended 16 or more class- es, 21 attended one to 15 classes, and 19 did not attend any classes. Overall, the mean participant attendance at citizenship classes—including those whose repeat participation exceeded the 16 “standard” class count —was 10.6±8.5. |
| Russinova 2018 | Employment | Vocational empowerment photovoice vs wait-list | NR | 10-weeks delivered in 2-hour group sessions which is followed by 2 booster sessions delivered a month apart after the completion of the core curriculum. | NR | NR | The VEP content was refined through an iterative process involving multiple pilots of its components and their implementation and one final feasibility pilot. An instrument was developed to assess the content and process fidelity to the VEP manual with 4-6 specific content items per session and 13 process items assessed on a 4-point scale with mean scores ranging from 1 (low fidelity) to 4 (high fidelity). | The VEP program was delivered at high fidelity over the course of the study with an average score of 3.85 for content fidelity and an average score of 3.91 for process fidelity. |
| Sacks 2004 | Offending | Prison Modified Therapeutic Community vs Mental Health Treatment program | Prison and community | Part 1 TC: The typical inmate attends formal program activities 5 days per week for 4–5 hours each day. Planned program duration is 12 months. Part 2 aftercare TC: program activities are seven days a week from 8 AM to 8 PM. The average resident attends formal program activities from 3 to 7 days per week for 3–5 hours each day during his 6-month tenure. | Part 1 TC typically 12 months but varies depending on the offender’s progress in treatment, the time required for approval to be placed in a community corrections facility, and the space available in the designated program facility. | NA | NR | NA |
| Sacks 2012 | Offending | Prison Modified Therapeutic Community vs Standard care | Prison and community | Participants attend formal program activities from 3 to 7 days a week for 3 to 5 hours a day - up to 6 months | NR | NA | NR | NA |
| Salzer 2016 | Rights | Peer-delivered core services of Centres for Independent Living (CILs) vs TAU | Community | The amount and frequency of sessions was driven by the participant as they were encouraged to work with the CPS. | The list and plan with the unmet needs were driven by the participant and would be adjusted based on their desire to pursue other areas or if a goal was met in a previously identified area. The amount and the frequency of sessions was driven by the participant and so varied. |  | There is no fidelity standard for CIL services as they are highly individualised based on identified needs and abilities of the patient. | NR |
| Sanches 2020 | Employment | Boston University approach to psychiatric rehabilitation vs active control condition | Outpatient | Participants were offered at least one session every 2 weeks - there was no predetermined total number of sessions. | The goals and the pace of the rehabilitation process are directed by the person with SMI, even though facilitated by a practitioner. | NR | BPR treatment fidelity was assessed retrospectively by independent BPR experts using the Fidelity of Rehabilitation instrument (FiRe) on a scale from 1 (lowest level of model adherence) to 5 (highest level). Fidelity scores were calculated for a random selection of two-thirds of BPR. | Program fidelity was insufficient in one-third of the BPR process. |
| Schene 2007 | Employment | Adjuvant occupational therapy + TAU vs TAU | Outpatient | Five contacts in first 4 weeks. Then 24 weekly group sessions in the following 24 weeks (2 hours long,8-10 patients) and 12 individual sessions. Then 3 individual visits in the follow-up phase (20 weeks) | 12 individual one to one sessions - individual exploration of work problems and support and evaluation of work resumption. | NA | Adherence: number of sessions of occupational therapy was recorded and qualitative data on treatment adherence in OT and satisfaction with OT were gathered by a study-specific questionnaire | NR |
| Segal 2010 | Rights | Self-help agencies and community mental health agency services, vs community mental health agency services only | Community | NR | NR | NR | NR | NR |
| Sheridan 2014 | Social Isolation | Supported socialisation vs control | Community | approximately 2 hours each week over a 9-month period, | NR | NA | NA | NA |
| Shern 2000 | Housing | Community outreach (Choices) vs Treatment as usual | Community | NR | NR | NA | NR | NR |
| Silverman 2014 | Social Isolation | Live music therapy and Education vs Education only | Inpatient | single session | NR | NA | NR | NA |
| Stergiopoluos 2015 | Housing | Housing first plus integrated case management vs treatment as usual | Community and residential | Participants were required to have weekly contact with a case manager. | NR | NA | NA | Service teams at all sites underwent repeated fidelity assessments to ensure adherence to HF principles and standards |
| Susser 1997 | Housing | Critical time intervention vs usual services | Community | 9 months | Some clients needed only a few follow-up visits. At the other extreme, some clients needed frequent visits for emotional support over several months and help with practical matters such as obtaining a minimum of furniture and locating inexpensive stores. | NA | NA | NA |
| Terzain 2013 | Social Isolation | social network intervention + TAU vs TAU only | Community | 6-month intervention | Personalised intervention- based on patient's areas of interest | NA | NR | NR |
| Tinland 2020 | Housing | Housing first + ACT vs treatment as usual | NR | Participants were provided with at least one weekly visit at home or in the city at times convenient to them. | NR | NR | Compliance with the recommendations for implementing the HF model of the US authors was verified at each stage using the HF model fidelity scale. | NR |
| Tsemberis 2004 | Housing | Pathways housing first vs continuum of care | Housing | The only requirement is that participants meet with a staff member twice a month at a minimum, however they can take advantage of all the ACT services which are available 7 days a week 24 hours a day. | Tailored to what the participant feels they need. | NA | NR | NR |
| Twamley 2019 | Employment | Compensatory cognitive training vs enhanced supported employment | NR | 12-weeks and each of the 12 CCT sessions were approximately 1 hour each. Participants in the CCT condition completed a mean of 8.23 CCT sessions and a mean of 4.04 supported employment sessions during the first 12 weeks of the study. | NR | NR | CCT sessions were audio-recorded and a random 20% of the sessions were coded for fidelity each month. | Our fidelity criteria were met in that >80% of sessions were rated at >80% fidelity and most sessions were rated at 90-100% fidelity to the CCT manual. |
| van Beurden 2017 | Employment | Occupational physician’s intervention vs TAU | Outpatient | 8-session training which takes place over 12 months. Consultations with the worker take place every 3 weeks during the first 3 months and then every 6 weeks thereafter. The OP contacts the supervisor/employer once a month. | NR | NR | NR | NR |
| Vauth 2005 | Employment | Computer assisted cognitive strategy training + Vocational rehabilitation vs vocational rehabilitation | Inpatient | The CAST was provided twice weekly, for 90 minutes, during 8 weeks. | NR | NA | NR | NR |
| Vlasveld 2013 | Employment | Collaborative care for major depression vs TAU | Community | 6-12 sessions of PST. 1 workplace intervention meeting. No details on other elements of the intervention. | NR | NA | The collaborative care treatment was closely monitored by the OP-CM, using the PHQ-9 as monitoring instrument. In order to enhance adherence to the treatment model, ongoing supervision and psychiatric consultation was provided to the OP-CMs. Also, a web-based tracking system was developed to support the OP-CM in monitoring treatment outcomes and in adhering to the stepped care protocol. In case of questions regarding the treatment, prescription of antidepressants, or (lack of) progress of the worker, the OP-CM was prompted by the web-based tracking system to consult the psychiatrist. | NR |
| Volker 2015 | Employment | E-Health cognitive web intervention vs TAU | Online | Number of sessions varied between 6 and 17 for different employees | The content of Return@work was tailor-made to the individual employee, depending on the symptoms and cognitions about RTW of the employee. Individuals were free to discuss topics or assignments with the occupational physician | NA | Actual care utilization in both groups was assessed with Trimbos/ iMTA questionnaire for Costs associated with psychiatric illness. The number of log-ins per participant and the number of modules of the intervention that they started, and the number of times the psychiatrist was consulted by the occupational physicians were recorded to assess adherence to the intervention. | NR |
| Yamaguchi 2017 | Employment | Cognitive remediation + supported employment vs usual employment services | Community and inpatient | Variable intervention length. Cognitive remediation programme - Thinking skills for work program: two sessions over 12 weeks | NR | NR | NR | NR |
| NR: Not recorded. NA: Not applicable | | | | | | | | |
