# Supplementary material for: Interventions to improve social circumstances of people with mental health conditions: a rapid evidence synthesis
Source: BMC Psychiatry. 2022 Apr 28;22:302. doi: 10.1186/s12888-022-03864-9 (PMC9047264; doi:10.1186/s12888-022-03864-9)
Supplement: Supplementary file 5 — Additional file 5. Housing First Trial additional papers. A list of additional publications relating to the Housing First Trial conducted in Canada, not extracted in this review due to irrelevant outcomes and overlapping data. [file 12888_2022_3864_MOESM5_ESM.docx]

**Sample from all 5 Cities**

Aubry, T., Tsemberis, S., Adair, C. E., Veldhuizen, S., Streiner, D., Latimer, E., ... & Hume, C. (2015). One-year outcomes of a randomized controlled trial of housing first with ACT in five Canadian cities. *Psychiatric Services*, *66*(5), 463-469.

Aubry, T., Goering, P., Veldhuizen, S., Adair, C. E., Bourque, J., Distasio, J., ... & Tsemberis, S. (2016). A multiple-city RCT of housing first with assertive community treatment for homeless Canadians with serious mental illness. *Psychiatric Services*, *67*(3), 275-281.

Stergiopoulos, V., Hwang, S. W., Gozdzik, A., Nisenbaum, R., Latimer, E., Rabouin, D., ... & Katz, L. Y. (2015). Effect of scattered-site housing using rent supplements and intensive case management on housing stability among homeless adults with mental illness: a randomized trial. *Jama*, *313*(9), 905-915.

Latimer, E. A., Rabouin, D., Cao, Z., Ly, A., Powell, G., Adair, C. E., ... & Moodie, E. E. (2019). Cost-effectiveness of Housing First intervention with intensive case management compared with treatment as usual for homeless adults with mental illness: secondary analysis of a randomized clinical trial. *JAMA network open*, *2*(8), e199782-e199782.

Additional publications

Latimer, E. A., Rabouin, D., Cao, Z., Ly, A., Powell, G., Aubry, T., ... & Veldhuizen, S. (2017). Costs of services for homeless people with mental illness in 5 Canadian cities: a large prospective follow-up study. *CMAJ open*, *5*(3), E576.

Poremski, D., Stergiopoulos, V., Braithwaite, E., Distasio, J., Nisenbaum, R., & Latimer, E. (2016). Effects of Housing First on employment and income of homeless individuals: results of a randomized trial. *Psychiatric Services*, *67*(6), 603-609.

Volk, J. S., Aubry, T., Goering, P., Adair, C. E., Distasio, J., Jette, J., ... & Tsemberis, S. (2016). Tenants with additional needs: When Housing First does not solve homelessness. *Journal of Mental Health*, *25*(2), 169-175.

Urbanoski, K., Veldhuizen, S., Krausz, M., Schutz, C., Somers, J. M., Kirst, M., ... & Goering, P. (2018). Effects of comorbid substance use disorders on outcomes in a Housing First intervention for homeless people with mental illness. *Addiction*, *113*(1), 137-145.

Kerman, N., Sylvestre, J., Aubry, T., & Distasio, J. (2018). The effects of housing stability on service use among homeless adults with mental illness in a randomized controlled trial of housing first. *BMC health services research*, *18*(1), 190.

Veldhuizen, S., Adair, C. E., Methot, C., Kopp, B. C., O’Campo, P., Bourque, J., ... & Goering, P. N. (2015). Patterns and predictors of attrition in a trial of a housing intervention for homeless people with mental illness. *Social psychiatry and psychiatric epidemiology*, *50*(2), 195-202.

Chung, T. E., Gozdzik, A., Palma Lazgare, L. I., To, M. J., Aubry, T., Frankish, J., ... & Stergiopoulos, V. (2018). Housing first for older homeless adults with mental illness: a subgroup analysis of the at home/Chez Soi randomized controlled trial. *International Journal of Geriatric Psychiatry*, *33*(1), 85-95.

O’Campo, P., Hwang, S. W., Gozdzik, A., Schuler, A., Kaufman-Shriqui, V., Poremski, D., ... & Addorisio, S. (2017). Food security among individuals experiencing homelessness and mental illness in the At Home/Chez Soi Trial. *Public health nutrition*, *20*(11), 2023-2033.

Adair, C. E., Streiner, D. L., Barnhart, R., Kopp, B., Veldhuizen, S., Patterson, M., ... & Goering, P. (2017). Outcome trajectories among homeless individuals with mental disorders in a multisite randomised controlled trial of housing first. *The Canadian Journal of Psychiatry*, *62*(1), 30-39.

Aquin, J. P., Roos, L. E., Distasio, J., Katz, L. Y., Bourque, J., Bolton, J. M., ... & Enns, M. W. (2017). Effect of Housing First on suicidal behaviour: a randomised controlled trial of homeless adults with mental disorders. *The Canadian Journal of Psychiatry*, *62*(7), 473-481.

**Sample from the Torronto Site only**

Stergiopoulos, V., Gozdzik, A., Misir, V., Skosireva, A., Connelly, J., Sarang, A., ... & McKenzie, K. (2015). Effectiveness of housing first with intensive case management in an ethnically diverse sample of homeless adults with mental illness: A randomized controlled trial. *PLoS One*, *10*(7), e0130281.

O'Campo, P., Stergiopoulos, V., Nir, P., Levy, M., Misir, V., Chum, A., ... & Hwang, S. W. (2016). How did a Housing First intervention improve health and social outcomes among homeless adults with mental illness in Toronto? Two-year outcomes from a randomised trial. *BMJ open*, *6*(9).

Additional publications

Stergiopoulos, V., Gozdzik, A., Misir, V., Skosireva, A., Sarang, A., Connelly, J., ... & McKenzie, K. (2016). The effectiveness of a Housing First adaptation for ethnic minority groups: findings of a pragmatic randomized controlled trial. *BMC Public Health*, *16*(1), 1110.

Stergiopoulos, V., Mejia-Lancheros, C., Nisenbaum, R., Wang, R., Lachaud, J., O'Campo, P., & Hwang, S. W. (2019). Long-term effects of rent supplements and mental health support services on housing and health outcomes of homeless adults with mental illness: extension study of the At Home/Chez Soi randomised controlled trial. *The Lancet Psychiatry*, *6*(11), 915-925.

**Sample from the Vancouver site only**

Somers, J. M., Moniruzzaman, A., Patterson, M., Currie, L., Rezansoff, S. N., Palepu, A., & Fryer, K. (2017). A randomized trial examining housing first in congregate and scattered site formats. *PloS one*, *12*(1), e0168745.

Somers, J. M., Moniruzzaman, A., & Palepu, A. (2015). Changes in daily substance use among people experiencing homelessness and mental illness: 24‐month outcomes following randomization to Housing First or usual care. *Addiction*, *110*(10), 1605-1614.

Patterson, M., Moniruzzaman, A., Palepu, A., Zabkiewicz, D., Frankish, C. J., Krausz, M., & Somers, J. M. (2013). Housing First improves subjective quality of life among homeless adults with mental illness: 12-month findings from a randomized controlled trial in Vancouver, British Columbia. *Social psychiatry and psychiatric epidemiology*, *48*(8), 1245-1259.

Additional publications

Palepu, A., Patterson, M. L., Moniruzzaman, A., Frankish, C. J., & Somers, J. (2013). Housing first improves residential stability in homeless adults with concurrent substance dependence and mental disorders. *American Journal of Public Health*, *103*(S2), e30-e36.

Somers, J. M., Patterson, M. L., Moniruzzaman, A., Currie, L., Rezansoff, S. N., Palepu, A., & Fryer, K. (2013). Vancouver At Home: pragmatic randomized trials investigating Housing First for homeless and mentally ill adults. *Trials*, *14*(1), 365.

Russolillo, A., Patterson, M., McCandless, L., Moniruzzaman, A., & Somers, J. (2014). Emergency department utilisation among formerly homeless adults with mental disorders after one year of Housing First interventions: a randomised controlled trial. *International Journal of Housing Policy*, *14*(1), 79-97.

Palepu, A., Patterson, M., Strehlau, V., Moniruzzamen, A., de Bibiana, J. T., Frankish, J., ... & Somers, J. (2013). Daily substance use and mental health symptoms among a cohort of homeless adults in Vancouver, British Columbia. *Journal of Urban Health*, *90*(4), 740-746.

Cheung, A., Somers, J. M., Moniruzzaman, A., Patterson, M., Frankish, C. J., Krausz, M., & Palepu, A. (2015). Emergency department use and hospitalizations among homeless adults with substance dependence and mental disorders. *Addiction science & clinical practice*, *10*(1), 17.

Parpouchi, M., Moniruzzaman, A., Russolillo, A., & Somers, J. M. (2016). Food insecurity among homeless adults with mental illness. *PLoS One*, *11*(7), e0159334.

Rezansoff, S. N., Moniruzzaman, A., Fazel, S., McCandless, L., Procyshyn, R., & Somers, J. M. (2017). Housing first improves adherence to antipsychotic medication among formerly homeless adults with schizophrenia: results of a randomized controlled trial. *Schizophrenia bulletin*, *43*(4), 852-861.

Noël, F., Moniruzzaman, A., Somers, J., Frankish, J., Strehlau, V., Schütz, C., & Krausz, M. (2016). A longitudinal study of suicidal ideation among homeless, mentally ill individuals. *Social psychiatry and psychiatric epidemiology*, *51*(1), 107-114.
