# Supplementary material for: Interventions to improve social circumstances of people with mental health conditions: a rapid evidence synthesis
Source: BMC Psychiatry. 2022 Apr 28;22:302. doi: 10.1186/s12888-022-03864-9 (PMC9047264; doi:10.1186/s12888-022-03864-9)
Supplement: Supplementary file 6 — Additional file 6. Mental health outcomes. Additional information on mental health outcomes of the interventions. [file 12888_2022_3864_MOESM6_ESM.docx]

Appendix 6: Mental health outcomes

| **Social Domain** | **Mental health diagnoses** | **Intervention strategies** | **Author** | **Intervention vs Control** | **Outcomes** |
| --- | --- | --- | --- | --- | --- |
| *Mental health disorder symptoms* | | | | | |
| Social Isolation | CMD | Changing cognitions | Conoley 1985 | Reframing vs Waitlist control | While there was no treatment effect at 1 month follow up (F(2, 108)=1.99), there was a significant time effect indicating that both groups improved in depressive symptoms on the Beck Depression Inventory (F(2, 108)=22.68, p<.01) and a treatment by time interaction (F(4, 108)=2.56, P<.05), with participants in the reframing group experiencing a more significant reduction in symptoms compared to the control group. |
|  |  | Psychoeducation | Haslam 2019 | Groups 4 Health social identity intevention vs TAU | Depression symptoms decreased significantly in the G4H condition, t(96.8) = -5.10, p < .001, and also in the TAU condition, t(91.7) = -2.65, p = .035. This corresponded to an average decrease of 7.29 points in the G4H condition (SE = 1.43; d = -0.67) and an average of 3.94 points in the TAU condition (SE = 1.49, d = -0.35). After the 2-month intervention, Reliable improvement was significantly more likely in the G4H group (45.2%) than the TAU group (25%; OR=2.38, 95% CI: 1.09, 5.18). |
|  |  | Supported socialisation | Lloyd-evans 2020 | Community navigator programme + routine care vs Routine care | [feasibility trial]  Mean depression scores on the PHQ-9 measure fell from 21.6 to 16.4 during the intervention period for the participants in the treatment group, compared to a fall from 21.1 to 18.8 in the control group. The change in depression score from baseline to follow up in the treatment group exceeds the established five-point threshold for clinically meaningful change on PHQ-9, however, the difference in mean scores between the intervention and control groups at 6 months (end of intervention) was not significant (Hedges g=0.37, 95% CI: -0.37, 1.11). |
|  | SMI | Changing cognitions | Roberts 2014 | Social cognition and interaction training vs TAU | Neither the Treatment group X Time interaction nor the main effect of treatment group was significant for PANSS Total symptoms (Hedges g at 6 months = 0.02, 95% CI: -0.49, 0.52) |
|  |  |  | Pos 2019 | CBT for social activation vs TAU | The CBT and control groups both improved significantly over time, however there was no difference between groups at either 3 months (end of treatment; Hedges g=-0.33. 95% CI: -0.06, 0.73) or 9 months from baseline (Hedges g=-0.05, 95% CI: -0.45, 0.34) in symptoms on the Global assessment of functioning. |
|  |  |  | Pot-Kolder 2018 | Virtual reality CBT vs TAU | At 3 months (End of intervention), paranoid thoughts were similar between the two groups (Hedges g=0.34, 95% CI: -0.02, 0.71), however, at 6 months post baseline, the virtual reality CBT group showed significantly lower levels of paranoid thoughts on the Green et al paranoid thought scale (Hedges g=0.50, 95% CI: 0.13, 0.87). |
|  |  | Supported socialisation | Davidson 2004 | Matched with a volunteer partner who had a personal history of psychiatric disability vs Matched with a volunteer partner who had no history of psychiatric disabilities vs Not matched with a volunteer partner | There was a main effect for Time in psychiatric symptom severity improvement, F (382)= 4.65, p= .01. Both groups experienced an improvement in symptoms, however, at 9 months (end of treatment), groups did not differ in BPRS scores compared to the control condition (Matched with partner with personal history of psychiatric disability: Hedges g=-0.27, 95% CI: -0.66, 0.12; matched with partner with no history of psychiatric disability: Hedges g=-0.13, -0.52, 0.26). |
|  |  |  | Priebe 2020 | Matched with a volunteer partner who had no history of psychiatric disabilities vs not matched with a volunteer partner | Psychotic symptoms (PANSS) were not significantly different between groups at 12 months (Hedges g=0.24, 95% CI: -0.17, 0.66). |
|  |  |  | Sheridan 2015 | Supported socialisation vs control | The treatment and control groups did not differ in BDI symptoms of depression at 4 months (Hedges g=0.22, 95% CI: -0.26, 0.69) and 10 months post baseline (Hedges g=-0.02, 95% CI: -0.39, 0.45), however, both groups showed significant reductions in symptoms over time (F=14.48, P=0.001) |
|  |  |  | Terzian 2013 | TAU plus social network intervention vs TAU only | There was no difference between the Social network intervention and the TAU group in the number of patients experiencing a reduction of more than 3 points on the brief psychiatric rating scale at both 12 months (OR=1.27, 95% CI: 0.84, 1.95) and 24 months from baseline (OR=1.34, 95% CI: 0.88, 2.05). |
|  |  | Changing cognitions Social skills training | Granholm 2005 | Cognitive behavioural social skills training vs TAU | At six months there was no significant difference in total psychotic symptoms measured on the PANSS between the social skills training and TAU groups (Hedges g=0.26, 95% CI: -0.19, 0.71) |
|  |  | Supported socialisation psychoeducation | Boevink 2016 | TREE Recovery programme + TAU vs TAU | At 12 months, there was no significant difference between the TREE program participants and the TAU participants in psychotic symptom severity measured on the Community Assessment of Psychic Experiences (CAPE): Hedges g=-0.05, 95% CI: -0.38, 0.29. |
|  | Mixed mental health conditions | Supported socialisation | Rivera 2007 | Peer-assisted case management vs Standard case management | Depressive symptoms on the Brief symptom inventory (BSI) were not significantly different between groups at either 6 months (Hedges g=-0.36, 95% CI: -0.71, -0.01) nor 12 months (Hedges g=-0.28, 95% CI: -0.63, 0.06) |
| Housing | SMI | Badged as Housing First Independent tenancy MDT mental health support | Aubry 2016 | Housing First + ACT vs TAU | There was no difference between groups in psychotic symptoms on the Colorado symptom index at 6 months (Hedges g= 0.11, 95% CI: -0.02, 0.24), 12 months (Hedges g=0.10, 95% CI: -0.02, 0.23) or 24 months (Hedges g=-0.09, 95% CI: -0.22, 0.04). However, both groups did report decreases in severity of psychiatric symptoms over time. |
|  |  |  | Stergiopaulos 2015 | Housing first plus integrated case management vs treatment as usual | Mean change from baseline did not differ significantly between the intervention and usual care groups at 24 months for severity of psychotic symptoms measured on the Colorado symptom index (0.57, 95% CI: -0.88, 2.01) adjusted for city and ethno-racial group. |
|  |  |  | Tinland 2020 | housing first vs treatment as usual | Overall, there was no statistically significant changes within the Housing first and TAU group's modified Colorado symptom index scores, with both groups improving scores from baseline to 24 months and the difference between them being non-significant at this point (Hedges g=0.08, 95% CI: -0.07, 0.23). |
|  |  |  | Tsemberis 2004 | Pathways housing first vs continuum of care | There was no group x time interaction for psychiatric symptom severity measured on the Colorado symptom index (F(4, 137)=0.35, P=0.85). |
|  |  | Staff on site (Residential only) MDT mental health support | Burnham 1996 | Social model residential treatment program vs control Social model non-residential treatment program vs control | at 9 months (end of treatment), the non-residential group showed significant reductions in psychotic symptom severity (mean difference=5.6), however the residential program showed an improvement which was not signfiicant (mean difference=4.0). Exposure to treatment vs control was not a signficiant predictor of psychotic symptom severity at end of treatment. |
|  |  | Specified psychological therapy | Elison 2020 | Manualized treatment model for co-occurring mental illness and substance use disorders (MISSION-Vet) vs TAU | Neither the ITT analysis nor the protocol adherent peer specialist analysis detected statistically significant effects of the intervention for any of the mental health measures. (no further detail) |
|  |  | MDT mental health support Specified psychological therapy | Fletcher 2008 | Integrated assertive community treatment vs standard care | Although on average, participants reduced both their psychiatric symptoms over time during the 30-month intervention, there was no difference in psychiatric symptoms (Brief psychiatric rating scale) at the end of the study (Hedges g=0, 95% CI: -0.35, 0.35) |
|  |  | Independent tenancy MDT mental health support | Goldfinger 1999 | Group housing vs independent housing | There was no significant difference in mental health symptoms (reported on the SF-36) at 18 months. (No additional information). |
|  |  | MDT mental health support | Lehman 1997 | ACT vs usual community services | Repeated-measures ANCOVA (controlling for race) for the Colorado symptom index total score revealed significant group (F=5.14, df= 1,122, P<.001) and time (F=9.16, df=3,363, P<.001) effects but no significant group X time effect. Unadjusted values showed that the ACT group showed significantly fewer symptoms at both 6 months (Hedges g=0.64, 95% CI: 0.31, 0.96) and 12 months (Hedges g=0.40, 95% CI: 0.04, 0.75). |
|  |  | MDT mental health support | Lipton 1988 | Residential treatment vs standard care | There was no significant difference in Colorado symptom index scores at follow-up. (no further detail) |
|  |  | Staff on site/supported housing MDT mental health support | McHugo 2004 | Integrated housing vs Parallel housing | At 6 months post baseline, there remained no differences in psychiatric symptoms on the Colorado symptom index between the two housing groups (Hedges g=0.32, 95% CI: -0.04, 0.68), however, at both 12 and 18 months post baseline, the integrated housing participants demonstrated significantly lower levels of psychiatric symptom severity (Hedges g=0.59, 95% CI: 0.23, 0.95 and Hedges g=0.36, 95% CI: 0.00, 0.72, respectively). |
|  |  | MDT mental health support | Morse 1992 | Continuous treatment team vs outpatient mental health services | There was no significant treatment by time interaction (F=0.45, p=.640) in psychiatric symptoms (global severity index), however a significant time interaction (F=54.83, p=.001) shows that symptoms reduced in both treatment and control group a 12 month follow up. |
|  |  | MDT mental health support | Morse 2006 | Integrated assertive community treatment vs standard care | at 24 months, there was no significant effect of treatment condition on the BPRS scale, F(2, 139) = 2.34, p = .10. There was a main effect of time, F(3, 438) = 41.72, p < .001, indicating that client symptoms improved over time regardless of treatment condition. |
|  |  | Staff on site/support housing housing support worker practical support | Shern 2000 | Community outreach (Choices) vs Treatment as usual | Psychotic symptoms on the Colorado symptom index were significantly lower at 24 months in the community outreach program compared to the control group (Hedges g=0.42, 95% CI: 0.12, 0.73). |
|  | Mixed mental health conditions | MDT mental health support | Morse 1997 | Broker case management vs ACT ACT with community workers vs ACT | At 18 months, significant treatment group effects were noted for two scales of the BPRS-thought disorder (F 3.91, df=2,123, p<.023) and activity level (F3.61, df2,123, p’ez.O3). A marginally significant treatment group effect (p< .065) was also noted on the withdrawal-elevated mood scale. Post hoc analyses indicated that clients in both assertive community treatment conditions had fewer symptoms in the areas of thought disorder and unusual activity than clients in the broker case management condition. No significant treatment group differences were found on the anxiety-depression scale, the hostility-suspicion scale, or the self-esteem scale. |
| Offending | SMI | Court-ordered treatment MDT mental health support Specified drug or alcohol programme offered | Cosden 2003/2005 | Mental health treatment court vs TAU | A repeated measures analysis found a significant time x condition interaction during the first 12 months F(2, 147) 3.39, p < 0.05, as well as a significant main effect of time. Clients in both groups had higher GAF scores of functioning at their 6-month follow-up than they had at intake, but the Mental health treatment court group improved more than the TAU group over this time. Both groups maintained this higher level of functioning at their 12-month follow-up. At 24 months there was a significant main effect of time, F(4, 132) = 17.76, p < 0.001), but no treatment effect, indicating that the two groups did not differ in improvement in symptoms. |
|  | Mixed mental health conditions | Specified psychological therapy | Kingston 2018 | Reasoning and rehabilitation2 + TAU vs TAU | At end of treatment, the reasoning and rehabilitation group had significantly lower measures of psychiatric symptoms (Hedges g=0.41, 95% CI: 0.02, 0.81) |
| Employment-sickness absence | CMD | Individual assessment | de Weerd 2016 | Work-focused CBT with convergence dialogue training vs work-focused CBT only | There was no significant difference between the intervention and control group in mental health outcome. Linear regression analysis showed that employees in the intervention group improved 5 points less on the Symptom checklist-90 compared with the employees in the control group when gender was adjusted for, although this difference was not statistically significant (B = 4.896, p = 0.706, 95% CI. –20.962–30.754) |
|  |  | Employer-run Individual assessment | Hees 2013 | Adjuvant occupational therapy vs TAU | Over 18 months follow up, participants in TAU+OT showed greater improvement in depression symptoms on the Hamilton Rating Scale for Depression (HAMD) than those in TAU. Though groups did not differ in symptoms at 6 or 12-months post baseline (Hedges g=0.17, 95% CI: -0.22, 0.55, Hedges g=0.35, 95% CI: -0.04, 0.74, respectively) at 18 months, the intervention group had significantly lower levels of depression symptoms (Hedges g=0.63, 95% CI: 0.24, 1.02). |
|  |  | Self management programme or psychological therapy Graded return to work | Noordik 2013 | Exposure based return to work intervention vs TAU | The overall differences between groups on the mean depression scores were not statistically significant at 6 months (Hedges g=-0.27, 95% CI: 0.62, 0.09) or 12 months (Hedges g=0.17, 95% CI: -0.20, 0.53). However, within both groups’ depression scores decreased significantly over time. |
|  |  | Employer run Self management programme or psychological therapy | Vlasveld 2013 | Collaborative care for major depression vs TAU | Depressive symptoms on the PHQ9 did not differ between groups at either 6-months (Hedges g=0.11, 95% CI: -0.30, 0.53) or 12 months from baseline (Hedges g=-0.26, 95% CI: -0.72, 0.20, P=1.329) |
| Employment-gaining and retaining | CMD | Supported employment (High fidelity IPS) | Davis 2018 | IPS vs transitional work | At 18 months post baseline, the IPS group PTSD symptoms on the PCL-5 had decreased (mean reduction from baseline = -3.66, vs -0.82 in the transitional work control), however there was no significant difference in between the groups in PTSD symptom improvement (Longitudinal analysis least squares mean difference=-1.9, 95% CI: -3.91, 0.12, P=.07). |
|  |  | Supported employment (low fidelity/not IPS) | Hellstrom 2017 | IPS modified for people with mood and anxiety disorders vs TAU | No significant difference was seen between groups in depressive symptoms (HAMD) at 12 months (Hedges g=0.04, 95% CI: -0.18, 0.26) and 24 months (Hedges g=-0.13, 95% CI: -0.34, 0.09). |
|  |  | Prevocational training (Job related skills training) | Schene 2007 | Adjuvant occupational therapy + TAU vs TAU | the BDI score decreased between baseline and 48 months from 23.6 to 14.0 for TAU and from 27.1 to 12.3 for TAU+OT, a significant time x condition interaction (p=0.015), which did not show during the first 12 months (p=0.950), but emerged between months 13 and 42 (p=0.032). |
|  | SMI | Prevocational training with cognitive therapy | Fowler 2019 | Social recovery CBT + TAU vs TAU | There was no significant difference between groups in psychotic symptoms (PANSS) at 9 months from baseline (end of treatment) (Hedges g=-0.01, 95% CI: -0.48, 0.46) and 24 months from baseline (Hedges g=-0.18, 95% CI: -0.67, 0.31). |
|  |  | Prevocational training (cognitive skills training) | Lindenmayer 2008 | Cognitive remediation vs computerized control | Mixed-effects linear analyses indicated significant improvements over time on the PANSS positive subscale (F=4.95, df=1 and 267, p=.027). The group-by-time interaction was not significant, suggesting similar changes over time for the two treatment groups. |
|  |  | Prevocational training (job related skills training) | Rogers 2006 | Psychiatric vocational rehabilitation vs enhanced state vocational rehabilitation | There was no significant difference between groups in symptoms measured on the brief psychiatric rating scale at 9 months from baseline (Hedges g=0.16, 95% CI: -0.18, 0.50) and 24 months from baseline (Hedges g=-0.08, 95% CI: -0.42, 0.25). |
|  |  | Transitional employment + Cognitive skills training | Lysaker 2005 | Vocational CBT program vs TAU | There was no significant difference between groups on PANSS total psychotic symptoms at 5 months (Hedges g=0.48, 95% CI: -0.10, 1.07) |
|  |  |  | McGurk 2016 | Enhanced vocational services cognitive remediation (thinking skills for work) vs enhanced vocational services only | There was no significant difference between groups in psychotic symptoms (PANSS) at either 3-months (end of intervention; Hedges g=0.35, 95% CI: -0.20, 0.91) or 18 months post baseline (Hedges g=0.18, 95% CI: -0.49, 0.84). |
|  |  |  | Vauth 2005 | Computer assisted cognitive strategy training + Vocational rehabilitation vs vocational rehabilitation | There was no significant difference in post treatment negative symptom scores between CAST and vocational rehabilitation (Hedges g=-0.06, 95% CI: -0.55, 0.43). |
|  |  | Augmented supported employment (SE + cognitive therapy) | Lecomte 2019 | CBT for supported employment vs supported employment | Both groups remained fairly stable over time regarding their overall symptoms and their positive symptoms of psychosis. At 7 months follow up, There was no significant difference between the CBT and control group (Hedges g=-0.02, 95% CI: -0.33, 0.28). |
|  |  | Augmented supported employment (SE + cognitive skills training) | McGurk 2007 | Supported employment + cognitive training vs supported employment | At 3 months (end of intervention), there was no significant difference in PANSS positive symptoms between the cognitive training group and the control group (Hedges g=-0.05, 95% CI: -0.64, 0.54). |
|  |  |  | McGurk 2015 | Enhanced supported employment + cognitive remediation (thinking skills for work) vs enhanced supported employment only | There was no significant difference between groups in psychotic symptoms at 24 months post baseline (No additional detail). |
|  |  |  | Twamley 2019 | Compensatory cognitive training vs enhanced supported employment | At 3 months (end of treatment) there was no significant difference between the groups in psychiatric symptoms (PANSS) (Hedges g=0.16, 95% CI: -0.16, 0.48). |
|  | Mixed mental health conditions | Prevocational training (Cognitive training) | Himle 2014 | Work-related CBT + vocational services vs vocational services only | [pilot trial]  Social anxiety symptoms (Liebowitz social anxiety scale) were significantly reduced in the intervention group compared to the TAU only group at both 1 month (end of treatment; Hedges g=0.71, 95% CI: 0.18, 1.24) and 4 months post baseline (Hedges g=0.73, 95% CI: 0.20, 1.27). |
|  |  | Supported employment (High fidelity IPS) | Reme 2019 | IPS vs TAU | The IPS group did not differ significantly from the control group at 6 months in anxiety and depressive symptoms (HADS total; Hedges g=-0.03, 95% CI: -0.23, 0.16), however, at 12 months follow up they showed significantly reduced symptoms compared to TAU (Hedges g=0.28, 95% CI: 0.08, 0.47). |
|  |  | Augmented supported employment (SE + cognitive skills training) | Christensen 2019 | IPS with enhancements vs TAU | There were no differences in improvements in positive psychotic symptoms at the 18-month follow up (Success rate difference= -0.037, 95% CI: 10.174, 0.102). |
|  |  |  | Yamaguchi 2017 | Cognitive remediation + supported employment vs usual employment services | There was no significant difference between groups in PANSS psychotic symptoms between the cognitive remediation group and the control group at 12 months (Hedges g=0.15, -0.30, 0.60). |
| Rights inclusion and citizenship | SMD | Advice and support services | Salzer 2016 | Peer-delivered core services of Centres for Independent Living (CILs) vs TAU | The intervention group recorded significantly higher scores on the recovery assessment scale at both 6 months (Hedges g=0.94, 95% CI: 0.53, 1.36) and 12 months (Hedges g=2.62, 95% CI: 2.08, 3.15). |
|  |  |  | Segal 2010 | Self-help agencies and community mental health agency services, vs community mental health agency services only | BPRS symptoms (F= 4.49, df=3 and 491, p<.004) dissipated more quickly and to a greater extent in the combined services condition than in the CMHA only condition. |
| Victimisation | SMI | Manualized group training | de Waal 2019 | Self-wise, other-wise, street-wise (SOS) training + TAU vs TAU | The SOS training group did not have significantly improved psychiatric symptoms compared to TAU at either 8-months (Hedges g=-0.14, 95% CI: -0.39, 0.10) or 14 months from baseline (Hedges g=-0.11, 95% CI: -0.36, 0.14). |
| *Service use* | | | | | |
| Social Isolation | SMI | Changing cognitions Social skills training | Granholm 2005 | Cognitive behavioural social skills training vs TAU | Both groups were equally as likely to hospitalised with 2 out of 39 TAU participants and 2 out of 37 intervention participants hospitalised (Odds of not going to hospital=0.95, 95% CI: 0.13, 7.09). |
| Housing | SMI | MDT mental health support | Lehman 1997 | ACT vs usual community services | ACT participants spent significantly fewer days in psychiatric hospitals (35.4 vs 66,9, Hedges g= 3.56, 95% CI: 3.00, 4.12). |
|  |  | MDT mental health support | Lipton 1988 | Residential treatment vs standard care | No difference was found in the mean number or length of re-admissions to psychiatric hospitals. However, There was significant difference in the proportion of the study year spent in a psychiatric hospital. 55 nights (15% of study year) in the intervention group and 168 nights (46% of study year) in the control group, Hedges g=1.27, 95% CI: 0.52, 2.02). |
|  |  | Badged as Housing First Independent tenancy MDT mental health support | Stergiopaulos 2015 | Housing first plus integrated case management vs treatment as usual | The proportion of participants reporting at least 1 hospitalization during the 24-month follow-up was 28.9% for the intervention group and 25.6% for the usual care group, a non-significant difference (OR=0.81, 95% CI: 0.63, 1.05). |
|  |  | Badged as Housing First Independent tenancy MDT mental health support | Tinland 2020 | Housing first vs treatment as usual | There were no significant differences between groups in the number of hospital admissions or emergency department visits between groups, however, the housing first group spent significantly less days in hospital (6 months Hedges g=3.72, 95% CI: 3.47, 3.96; 12 months Hedges g=2.37, 95% CI: 2.18, 2.56; 24 months Hedges g=1.96, 95% CI: 1.78, 2.14). |
| Offending | SMI | MDT mental health support Specified drug and alcohol programme | Chandler 2006 | Integrated dual diagnosis treatment post-custody vs usual post-custody services | A marginally significant difference in the study period (up to 30 months) favouring the experimental group regarding whether participants had any hospital days (Likelihood ratio chi-square = 5.20, df = 2, p < .074) was found (no additional information). |
|  |  | Court-ordered treatment MDT mental health support | Lamberti 2017 | Forensic ACT vs Enhanced TAU | At the end of the intervention, there was no difference between groups in the mean number of days spent in hospital (Hedges g=0.41, 95% CI: -0.06, 0.88). |
| Employment | SMI | Augmented supported employment (SE + cognitive skills training) | Christensen 2019 | IPS with enhancements vs TAU | There was no significant difference in hospitalisations at 18 months between the two groups (Hedges g=0.12, 95% CI: -0.06, 0.30). |

*Note. N: number of participants. SMI: Severe mental illness. TAU: treatment as usual. CBT: cognitive behavioural training ACT: Assertive Community Treatment OR: Odds ratio. CI: Confidence interval. Y: Yes. N: No*
