# Supplementary material for: Interventions to improve social circumstances of people with mental health conditions: a rapid evidence synthesis
Source: BMC Psychiatry. 2022 Apr 28;22:302. doi: 10.1186/s12888-022-03864-9 (PMC9047264; doi:10.1186/s12888-022-03864-9)
Supplement: Supplementary file 7 — Additional file 7. Quality of life outcomes. Additional information on quality of life outcomes of the interventions. [file 12888_2022_3864_MOESM7_ESM.docx]

Appendix 7 Quality-of-life outcomes

| **Social Domain** | **Mental health diagnoses** | **Intervention strategies** | **Author** | **Intervention vs Control** | **Outcomes** |
| --- | --- | --- | --- | --- | --- |
| *Quality of life* | | | | | |
| Social Isolation | CMI | Supported socialisation | Lloyd-evans 2020 | Community navigator programme + routine care vs Routine care | Mean quality of life on the EuroQoL measure improved more (0.283 to 0.472) in the intervention group compared to an improvement of 0.4 to 0.453 in the control group. However, differences at 6 months (end of intervention) were not significant (Hedges g=0.06, 95% CI: -0.67, 0.79). |
|  | SMI | Psychoeducation Supported socialisation | Boevink 2016 | TREE Recovery programme + TAU vs TAU | 12 months from baseline, the TREE recovery programme group did not show any significantly improved quality of life scores on the Lancashire quality of life Profile compared to the TAU group (Hedges g=0.11, 95% CI: -0.22, 0.45) |
|  |  |  | Castelein 2008 | Guided peer support VS TAU | While there appeared to be a significant difference in favour of the Guided peer support group in quality of life scores on the WHO quality of life scale at end of treatment (Hedges g=0.40, 95% CI: 0.01, 0.78), this difference did not remain significant when adjusting for baseline values (P=0.87). |
|  |  | Social skills training | Glynn 2004 | Skills training + generalization vs skills training only | Groups did not show significant differences at 12 months on the Quality of life scale (adjusting for baseline scores; Hedges g=0.14, 95% CI: -0.44, 0.72). |
|  |  | Supported socialisation | Priebe 2020 | Matched with a volunteer partner who had no history of psychiatric disabilities vs ot matched with a volunteer partner | There were no significant differences in scores on the Quality of life scale at 12 months (end of intervention; Hedges g=0.15, 95% CI: -0.26, 0.57). |
| Housing | SMI | Badged as Housing First Independent tenancy MDT mental health support | Aubry 2016 | Housing First + ACT vs TAU | At 12 months, both the housing first and TAU groups reported significant gains in quality of life. However, the absolute gain was significantly greater for the housing first participants (z=.16, p<.001). Group differences, adjusted for baseline score, sex, race and site were 5.02 (95% CI: 2.66, 7.38) at 6 months and 7.27 (95% CI: 3.84, 10.69) at 12 months. The adjusted SMI of QOLI-20 scores between groups at 12 months was 0.31 (95% CI: 0.16–0.46). At 24 months the housing first participants continued to have a higher average score on the QOLI-20 (adjusted SMI=0.15, p<.01, 95% CI=0.04, 0.24). |
|  |  |  | Stergiopaulos 2015 | Housing first plus integrated case management vs treatment as usual | There was no significant difference between groups in change in EQ-5D quality of life scores at 6 months (2.11, 985% CI: -1.00, 5.23, P=0.18), 12 months (0.91, 95% CI: -2.18, 4.00, P=0.56) or 24 months (0.10, 95% CI: -2.92, 3.13, P=0.95) when adjusting for study city and ethno-racial group. |
|  |  |  | Tinland 2020 | Housing first vs treatment as usual | The housing first group showed significantly higher total quality of life scores at 6 months (Hedges g=0.17, 95% CI: 0.02, 0.32) and 24 months (Hedges g=0.36, 95% CI: 0.21, 0.51). At 12 months The housing first group also showed higher quality of life scores, however, this difference did not reach significance. (Hedges g=0.14, 95% CI: -0.01, 0.29) |
|  |  | Staff on site/support housing housing support worker practical support | Shern 2000 | Community outreach (Choices) vs Treatment as usual | Individuals in the intervention group reported larger improvement in overall life satisfaction at 24 months (t=-4.21, p<.001) |
| Offending | SMI | Court-ordered treatment MDT mental health support Specified drug or alcohol programme offered | Cosden 2005 | Mental health treatment court vs TAU | There was no significant difference between groups at 6 months (Hedges g=0.01, 95% CI: -0.25, 0.27) and 12 months (Hedges g=0.06, 95% CI: -0.20, 0.32), though both groups demonstrated improvement over time. At 24 months there was a significant treatment by time interaction such that while both groups continued to improve in ratings of quality of life, the mental health treatment court participants’ scores improved more over time. |
| Employment- gain and retain | CMI | Supported employment (Low fidelity/not IPS) | Hellstrom 2017 | IPS modified for people with mood and anxiety disorders vs TAU | The IPS groups showed no difference in quality of life scores on the WHO-5 compared to TAU at 12 months (Hedges g=0.28, 95% CI: -0.04, 0.59) or 24 months (Hedges g=0.13, 95% CI: -0.22, 0.48). |
|  | SMI | Prevocational training (Job related skills training) | Bell 2003 | Paid work plus behavioural intervention vs paid work only | There was no significant difference between the two groups on the quality of life scale interpersonal function subscale at 6 months (Hedges g=0.26, 95% CI: -0.23, 0.76). |
|  |  |  | Bell 2018 | Vocational rehabilitation + cognition remediation vs vocational rehabilitation + cognitive games | A time by condition interaction favoured the vocational rehabilitation + cognitive remediation group on total quality of life over 12 months (p<.02). Quality of life measures remained stable over time in this group (p<.05) but declined significantly for vocational rehabilitation + cognitive games group (P<.01). However, There were no significant differences between groups at 6 months (Hedges g=-0.02, 95% CI: -0.53, 0.49) or 12 months (Hedges g=0.24, 95% CI: -0.29, 0.76). |
|  |  |  | Rogers 2006 | Psychiatric vocational rehabilitation vs enhanced state vocational rehabilitation | Psychiatric vocational rehabilitation did not provide a benefit to quality of life compared to enhanced state vocational rehabilitation at either 9-months (Hedges g=-0.08, 95% CI: -0.42, 0.25), or 24 months (Hedges g=-0.11, 95% CI: -0.45, 0.23). |
|  |  | Augmented supported employment (SE + cognitive skills training) | McGurk 2015 | Enhanced supported employment + cognitive remediation (thinking skills for work) vs enhanced supported employment only | There was no significant difference between groups at 24 months in quality of life. (no further detail). |
|  |  |  | Twamley 2019 | Compensatory cognitive training vs enhanced supported employment | ANCOVAS controlling for age, education, sex, minority status, and number of intervention sessions showed that at end of intervention (3 months), the compensatory cognitive training had significant improvements in quality of life compared to the enhanced supported employment control group (F=9.67, partial η2 = 0.11, P=0.003). |
|  | Mixed mental health conditions | Supported employment (High fidelity IPS) | Reme 2019 | IPS vs TAU | While differences in health-related quality of life did not emerge at 6 months (Hedges g=0.11, 95% CI: -0.08, 0.31), at 12 months follow up the IPS groups showed a small significant improvement compared to TAU (Hedges g=0.26, 95% CI: 0.06, 0.45). |
| Rights, inclusion and citizenship | SMI | Advice and support services | Sanches 2020 | Boston University approach to psychiatric rehabilitation vs active control condition | The rate of improvement did not differ between conditions for quality of life, though for both groups it improved over the study period (t-ratio=3.32, df=237, P=0.001). |
|  | Mixed mental health conditions |  | Salzer 2016 | Peer-delivered core services of Centres for Independent Living (CILs) vs TAU | Time X Group interactions in repeated measures ANOVA showed no significant differences between the CIL and control condition over time on quality of life (F(2, 172)=1.86, P=0.16). However, post-hoc analyses of least squared means showed that CIL participants experienced a small increase over time on QoL between baseline and 12 months (d=0.38, SE=0.12, t=3.21, P=0.01) while control participants did not. |
| *Life satisfaction* | | | | | |
| Social Isolation | Mixed mental health conditions | Supported socialisation | Rivera 2007 | Peer-assisted case management vs Standard case management | There was no significant effect of the intervention on life satisfaction at 6 months (Hedges g=-0.36, 95% CI: -0.70, 0.00) or 12 months (Hedges g=-0.21, 95% CI: -0.55, 0.14). |
| Housing | SMI | MDT mental health support | Lehman 1997 | ACT vs usual community services | When adjusting for race, ACT patients expressed greater life satisfaction than the comparison patients (MANCOVA adjusting for race F=2.93, df=8, 139, P=0.005). At 12 months there were no significant differences between groups (no further detail). |
|  |  | Staff on site/supported housing MDT mental health support | McHugo 2004 | Integrated housing vs Parallel housing | While both programs resulted in improved measures of life satisfaction after 18 months (F=7.30, P<.05), The integrated housing group showed larger improvements (F=6.35, P<.05). While at 6 months and 12 months there was no difference in Lehman quality of life scale measures between groups (6 months: Hedges g=0.27, 95% CI: -0.08, 0.63, P=0.134; 12 months: Hedges g=0.33, 95% CI: -0.03, 0.69), by 18 months life satisfaction ratings were slightly higher in the integrated group (Hedges g=0.38, 95% CI: 0.02, 0.74). |
| *Wellbeing* | | | | | |
| Social Isolation | SMI | Supported socialisation | Davidson 2004 | Matched with a volunteer partner who had a personal history of psychiatric disability vs Matched with a volunteer partner who had no history of psychiatric disabilities vs Not matched with a volunteer partner | There were no statistically significant effects of time or group on wellbeing at 9 months (end of treatment). |

*Note. N: number of participants. SMI: Severe mental illness. TAU: treatment as usual. CBT: cognitive behavioural training ACT: Assertive Community Treatment OR: Odds ratio. CI: Confidence interval. Y: Yes. N: No*
