# Supplementary material for: Interventions to improve social circumstances of people with mental health conditions: a rapid evidence synthesis
Source: BMC Psychiatry. 2022 Apr 28;22:302. doi: 10.1186/s12888-022-03864-9 (PMC9047264; doi:10.1186/s12888-022-03864-9)
Supplement: Supplementary file 8 — Additional file 8. Costs. Additional information on cost analyses reported within studies. [file 12888_2022_3864_MOESM8_ESM.docx]

Sixteen studies were identified with sufficient information to enable them to be classified as economic evaluations (Table N). We have defined an evaluation broadly to include cost comparisons (n=6) as well as studies which have combined costs and outcomes either directly in the form of a ratio (n=6) or return on investment (n=2) or indirectly where cost and outcomes are reported alongside each other (n=2).

Six studies evaluated interventions to address issues of housing or homelessness in people with severe mental illness (SMI). Two from Canada (Aubry et al, 2016; Latimer et al, 2019) were linked and found a housing support service to result in more stable housing days and at a cost that was largely offset by reduced costs elsewhere. Another evaluation of this intervention from France (Tinland et al, 2020) showed modest cost savings. In the USA, Dickey et al (1997) compared costs of support for independent living with residential care and found the former to be substantially lower. Another study revealed that early support for people with SMI and experiencing homelessness can achieve better outcomes at similar costs compared to usual care (Jones et al, 2003). Elsewhere, it was found that integrated assertive treatment resulted in substantial cost savings compared to usual care and satisfaction with care was greater (Morse et al, 2006). Interestingly, these authors also found that assertive treatment on its own, while reducing costs, did not result in greater satisfaction.

Two studies, both from the USA, of interventions for people with SMI and experience of using criminal justice services show inconclusive results. They reveal that costs are higher than for usual care but without outcomes clearly reported or cost-offsets demonstrated (Cusack et al, 2010; Chandler & Spicer, 2006).

There were six studies that evaluated vocational interventions. In the Netherlands, two studies both demonstrated that interventions to reduce work loss for people with common mental disorder were relatively cost-effective (Schene et al, 2006; Lokman et al, 2017), while similar results were found for an intervention to train managers in mental health issues (Milligan-Saville et al, 2017). In Japan, cognitive remediation with employment support for people with SMI was also found to have a high likelihood of being cost-effective (Yamaguchi et al, 2017). In the USA, Okapku et al (1997) found the costs of employment-orientated case management unlikely to be offset by reduced work loss costs but this study did not extend to looking at other effects. In another evaluation, a CBT focussed vocational intervention applied to police officers in the Netherlands was found to reduce costs and produce similar outcomes compared to usual care but there was extensive uncertainty around the findings (Rebergen et al, 2009).

Finally, two studies from the Netherlands evaluated specific interventions for people with SMI. One of these focussed on guided peer support (Castelein et al, 2008). Costs were not presented in detail and no clear differences from usual care were identified. The other study compared virtual reality-based CBT with usual care (Pot-Kolder et al, 2020). The intervention resulted in increased costs, better social participation outcomes, and more quality-adjusted life years (QALYs). The incremental cost of one more person with improved social participation was €10,069 and the incremental cost per QALY was €48,868.

Overall, the evidence is reasonably strong in favour of social interventions, particularly when these focussed on housing and employment. Only a small number of studies measured outcomes using quality adjusted life years (QALYs). Use of QALYs can help decision makers to compare across different areas of health, but they focus on functioning and health status rather the achievement of specific social outcomes. As such it was not unexpected to see them rarely used in the evaluations reviewed here, and indeed their use may not have been appropriate. While many studies measured costs relatively comprehensively, recognising the multifaceted nature of mental health problems, a few did take a particularly narrow perspective and so conclusions from these need to be tempered with caution. We also need to be careful in placing too much emphasis on evaluations that only compare costs and that was the case for many of those reviewed.

**Appendix 8. Summary of evidence from economic evaluations.**

| **Study details** | **Comparison** | **Costs included** | **Mean costs per group** | **Main economic result** |
| --- | --- | --- | --- | --- |
| Aubry et al (2016)  Canada  SMI & housing issues  24-month follow-up | Housing First + assertive community treatment (HF+ACT, n=469)  TAU (n=481) | Intervention  Mental health services  Other health services  Social care  Criminal justice | Price year: not stated  Intervention: C$22,257 annually HF+ACT  Net cost-offset of C$21,367 annually. | 96% of intervention cost was offset by reduced cost of other services. |
| Latimer et al (2019)  Canada  SMI & housing issues  24-month follow-up | Housing First (HF) intervention help with housing costs and to provide case management (n=689)  TAU (n=509) | Intervention  Health services  Income  Welfare benefits  Criminal justice | Price year: 2011  Total: C$48716 HF, C$40,849 TAU | HF resulted in more stable housing days. Cost per extra day was C$56. Very likely to be cost-effective at most thresholds. |
| Tinland et al (2020)  France  SMI & housing issues  24-month follow-up | Housing First plus ACT (n=353)  TAU (353) | Intervention  Health services  Housing costs  Criminal justice  Welfare benefits | Price year: 2016  Health: €29454 HF, €47570 TAU; Total: €76808 HF, €76825 TAU | HF cost on average €217 less than TAU |
| Dickey et al (1997)  USA  SMI & housing issues  18-month follow-up | Evolving Consumer Housing (ECH, n=61)  Independent living (IL, n=51) | Intervention  Housing  Mental health services  Jail | Price year: 1994  Total health and housing: $56434 ECH, $29838 IL | Costs substantially lower for those who can be supported to live independently. |
| Jones et al (2003)  USA  SMI & homelessness  18-month follow-up | Critical time intervention (CTI, n=48)  TAU (n=48) | Intervention  Other mental health services  Non-mental health services  Housing  Criminal justice  Welfare benefits | Price year: 1992  Total: $52374 CTI, $51649 TAU | CTI resulted in 60 fewer days homeless. CTI cost-effective at thresholds above $152 per homeless day avoided. |
| Morse et al (2006)  USA  SMI & housing issues & substance abuse  24-month follow-up | Integrated assertive community treatment (IACT, n=46)  Assertive community treatment only (ACTO, n=54)  TAU (n=49) | Intervention  Mental health services  Other health services  Shelters | Price year: Not stated  Total services: $48764 IACT; $71211 ACTO; $41726 TAU | Not reported but satisfaction with IACT and ACTO greater than TAU and so IACT appears most cost-effective |
| Castelein et al (2008)  Netherlands  SMI  Eight-month follow-up | Guided peer support (n=56)  TAU (n=50) | Intervention  Health services | Price year: 2003/4  Intervention: up to €250 per patient  Total: approximately €5750 in each group | No clear differences in costs between groups. |
| Chandler & Spicer (2006)  USA  SMI & forensic history & substance abuse  18-month average follow-up | Integrated dual diagnosis treatment (IDDT) post-custody (n=103)  TAU (n=79) | Mental health services  Jail | Price year: not stated  Mental health: $9176 IDDT, TAU $6318 TAU per year  Total: $14809 IDDT, $11069 TAU | Costs higher for intervention but differences at baseline make interpretation difficult. |
| Cusack et al (2010)  USA  SMI & forensic history  12 and 24 month follow-up | Forensic ACT (FACT, n=72)  TAU (N=62) | Mental health services  Jail | Price year: not stated  0-12 months  Inpatient: $5530 FACT, $8827 TAU  Outpatient: $13481 FACT, $5118 TAU  Jail: $1848 FACT, $3530 TAU  13-24 months  Inpatient: $4296 FACT, $7141 TAU  Outpatient: $7836 FACT, $4249 TAU  Jail: $2050 FACT, $3046 TAU | Increased outpatient costs for intervention were offset to some extent by reduced costs elsewhere. |
| Schene et al (2006)  Netherlands  Depression & work loss | Adjuvant occupational therapy (n=30)  TAU (n=32) | Intervention  Mental health services  Other health services  Patient | Price year: Not stated  Total services: $3149 AOT, $1891 TAU | At a value of $36.88 per work day the intervention had a 75.5% likelihood of being cost-effective |
| Okapku et al (1997)  USA  SMI/CMD & benefit recipients  4-month follow-up | Employment-orientated case management (EOCM, n=73)  TAU (n=73) | Intervention  Work time | Price year: Not stated  Intervention: $807 | Value of employment for EOCM was $615 more per participant than for TAU. Intervention deemed not be cost-beneficial but other effects not included. |
| Rebergen et al (2009)  Netherlands  CMD in the police force  12-month follow-up | Guideline Based Care (GBC) – employment support with CBT elements (n=125)  Usual employment support (UC, n=115) | Health services  Lost production | Price year: 2003  Health services: €2145 GBC, €2664 UC  Productivity loss: €18801/€14114/€11691 GBC depending on method, €18192/€14202/€11503 UC | GBC reduced costs and produced similar outcomes. Low probability of cost-effectiveness due to uncertainty. |
| Yamaguchi et al (2017)  Japan  SMI  12-month follow-up | Cognitive remediation and supported employment (CRT, n=57)  Traditional vocational services (TVS, n=54) | Intervention  Health services  Social care | Price year: 2012  Total: $9823 CRT, $11063 TVS | Cost difference in favour of CRT but not significant. Very high probability of intervention being cost-effective using vocational outcomes |
| Lokman et al (2017)  Netherlands  CMD & employment  12-month follow-up | Web-based tool to aid return to work (n=131)  TAU (n=89) | Intervention  Health  Travel  Absenteeism  Presenteeism | Price year: 2011  Lost production: €20205 ECO, €23691 TAU  Total: €25383 ECO, €29893 TAU | From the employer’s perspective the ROI is €10.60  From societal perspective the ROI is €14.00 |
| Milligan-Saville et al (2017)  Australia  CMD & employment | Mental health training for managers (n=65)  TAU (n=63) | Intervention | Price year: Not stated  Intervention: £626 | Intervention resulted in lost work costs that were £6244 less than TAU. ROI was £9.98 |
| Pot-Kolder et al (2020)  Netherlands  SMI  6-month follow-up | Virtual reality-based CBT (VR-CBT, n= 58)  TAU (n=58) | Intervention  Health  Travel  Productivity | Price year: 2015  Health services: €3917 VR-CBT, €1686 TAU  Societal: €4393 VR-CBT, €2050 TAU | Incremental cost per person with positive social participation outcome was €10069  Incremental cost per QALY was €48868 |

*Note. N: number of participants. SMI: Severe mental illness. TAU: treatment as usual. CBT: cognitive behavioural training ACT: Assertive Community Treatment OR: Odds ratio. CI: Confidence interval. Y: Yes. N: No*
